# Dicationic styryl dyes for colorimetric and fluorescent detection of nucleic acids

**DOI:** 10.1038/s41598-022-18460-w

**Published:** 2022-08-22

**Authors:** Kotchakorn Supabowornsathit, Kriangsak Faikhruea, Boonsong Ditmangklo, Theeranuch Jaroenchuensiri, Sutthida Wongsuwan, Sirikarn Junpra-ob, Ilada Choopara, Tanapat Palaga, Chanat Aonbangkhen, Naraporn Somboonna, Jaru Taechalertpaisarn, Tirayut Vilaivan

**Affiliations:** 1 Organic Synthesis Research Unit, Department of Chemistry, Faculty of Science, Chulalongkorn University, Phayathai Road, Pathumwan, Bangkok, 10330 Thailand; 2 Department of Chemistry, Faculty of Science, Center of Excellence in Natural Products Chemistry (CENP), Chulalongkorn University, Phayathai Road, Pathumwan, Bangkok, 10330 Thailand; 3 Department of Microbiology, Faculty of Science, Chulalongkorn University, Phayathai Road, Pathumwan, Bangkok, 10330 Thailand; 4 Microbiome Research Unit for Probiotics in Food and Cosmetics, Chulalongkorn University, Bangkok, 10330 Thailand; 5 Department of Chemistry and Biochemistry, University of California, Santa Cruz, CA 95064 USA

**Keywords:** DNA, Nucleic acids, Chemical synthesis, Fluorescent labelling

## Abstract

Nucleic acid staining dyes are important tools for the analysis and visualizing of DNA/RNA in vitro and in the cells. Nevertheless, the range of commercially accessible dyes is still rather limited, and they are often very costly. As a result, finding nontoxic, easily accessible dyes, with desirable optical characteristics remains important. Styryl dyes have recently gained popularity as potential biological staining agents with many appealing properties, including a straightforward synthesis procedure, excellent photostability, tunable fluorescence, and high fluorescence quantum yield in the presence of nucleic acid targets with low background fluorescence signals. In addition to fluorescence, styryl dyes are strongly colored and exhibit solvatochromic properties which make them useful as colorimetric stains for low-cost and rapid testing of nucleic acids. In this work, novel dicationic styryl dyes bearing quaternary ammonium groups are designed to improve binding strength and optical response with target nucleic acids which contain a negatively charged phosphate backbone. Optical properties of the newly synthesized styryl dyes have been studied in the presence and absence of nucleic acid targets with the aim to find new dyes that can sensitively and specifically change fluorescence and/or color in the presence of nucleic acid targets. The binding interaction and optical response of the dicationic styryl dyes with nucleic acid were superior to the corresponding monocationic styryl dyes. Applications of the developed dyes for colorimetric detection of DNA in vitro and imaging of cellular nucleic acids are also demonstrated.

## Introduction

Optical detection of nucleic acids is important because nucleic acids carry genetic information and play indispensable roles in the production of proteins that are essential for the structure and functions of all organisms. However, since nucleic acid molecules themselves have no intrinsic properties that allow direct optical detection with sufficient sensitivity, a signaling label is often required to improve the sensitivity of the assays. The label may be attached to the nucleic acid molecule either through a covalent bond^1^ to form a probe, or by noncovalent binding to a specific sequence or structure of nucleic acid targets^2^. The latter is more convenient as no complicated modification and purification steps are required although the specificity may not be as good as the probe-based assay. For a label that exhibits permanent signals, a mechanism to distinguish between positive and negative events must be incorporated. This is typically performed in a heterogeneous assay format whereby the bound and unbound labels (or labeled probes) are partitioned in separated phases, followed by washing to eliminate the unbound labels or probes. Alternatively, the use of smart labels or probes that can exhibit the signal in response to the nucleic acid structures or sequences of interest allows the assay to be performed in a homogeneous format that is more convenient and can be applied for the detection of nucleic acid targets in living cells.

In recent years, fluorescent nucleic acid stains have become an essential tool for qualitative and quantitative analysis of nucleic acids both in vitro and in the cells. These dyes are typically conjugated organic molecules that can bind non-covalently to nucleic acids in one or more of several possible modes including intercalation, groove binding, aggregation, and electrostatic interaction^3^. Ethidium bromide is a classic example of a DNA intercalator that became highly fluorescent upon binding to double-stranded DNA (dsDNA) thus it used to be a popular staining agent for visualizing nucleic acids following gel electrophoresis^4–6^. Nevertheless, ethidium bromide is well-known for its toxicity and mutagenic properties^7^. There have been several commercially available alternative nucleic acid staining dyes—some of which can be used for imaging cellular nucleic acids, but they are costly and suffers several other limitations. Consequently, the development of alternative dyes which are non-toxic, readily accessible, with tunable optical properties and target binding affinity/selectivity is still desirable and challenging^8^.

Styryl dyes, as a class of conjugated organic dyes, consisted of an electron-rich aromatic ring system connecting to an electron-deficient heteroaromatic ring system through one or more conjugated double bonds^9^. The intramolecular charge transfer (ICT) properties of such dyes make their optical properties highly sensitive to environment^10^ like solvent polarity, viscosity, or pH, leading to visible optical response in terms of color and/or fluorescence change. Also, styryl dyes are easy to synthesize and show high photostability as well as high fluorescence quantum yields (*Φ*_F_). These dyes have found widespread uses in various applications such as sensitizers and additives in photographic industries^11,12^, molecular photovoltaic cells^13^, optical molecular systems^14^, and artificial photosynthesis^15^. In the context of fluorescent probes, styryl dyes have been utilized for chemo/biosensing of peroxidase activities^16^, selected ions^17^, or biomolecules such as proteins and nucleic acids^18^. More importantly, styryl dyes have recently emerged as an attractive candidate for tissue and cellular staining agents^19–21^. In addition, styryl dyes may potentially serve as colorimetric nucleic acid stains due to their solvatochromic properties, which make it useful for the development of low cost and rapid assay of nucleic acids^22^. Apart from the widely used gold nanoparticles^23–25^, only a few colorimetric dyes for DNA are known. These include merocyanine^26–28^ and crystal violets^29,30^. No colorimetric detection of nucleic acids based on styryl dyes has been reported, although the change of absorption maxima up to a few tens of nanometers has been previously noted in some styryl dyes upon binding to nucleic acids^31^.

**Figure 1:**
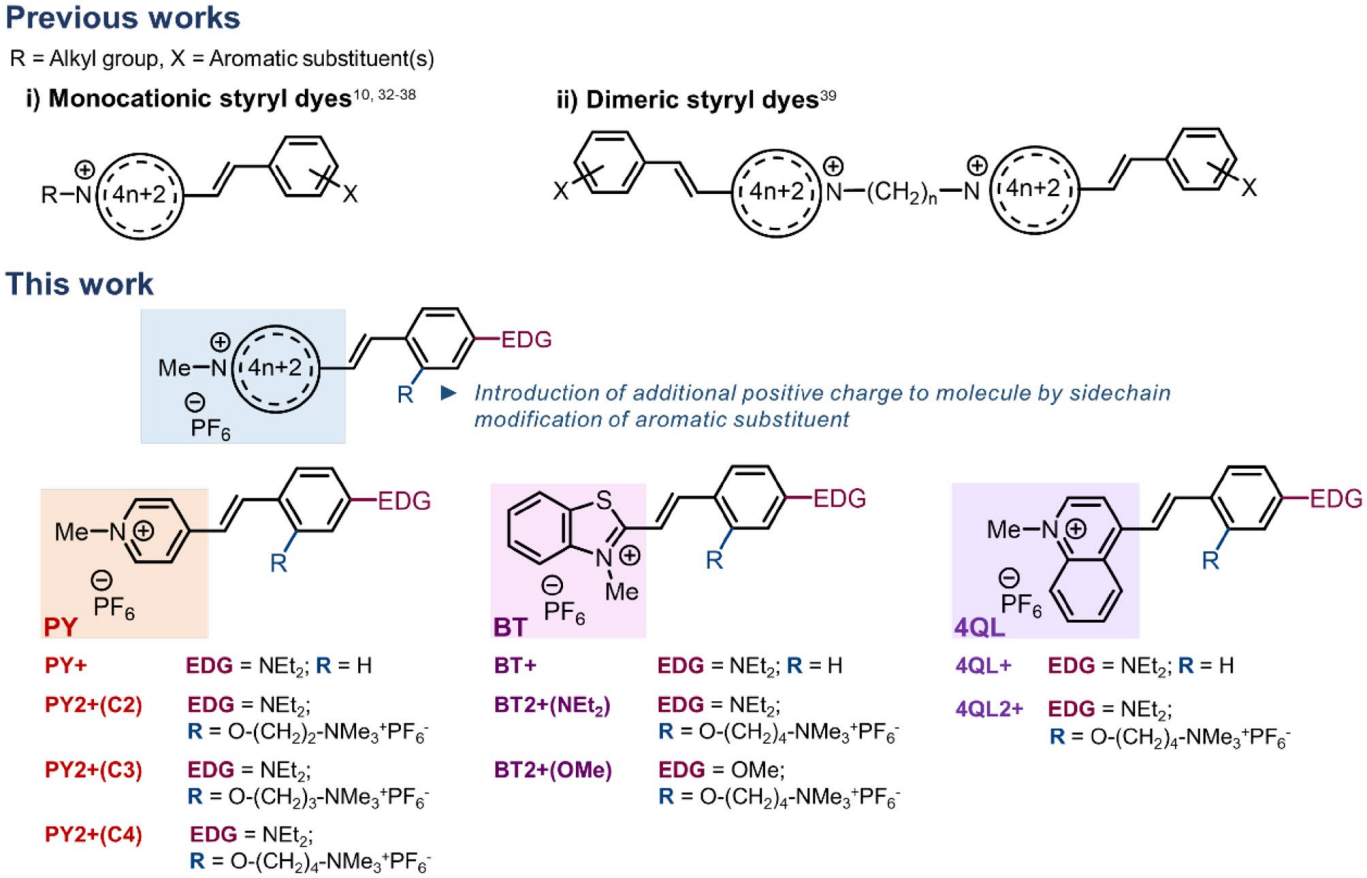
The designs of cationic styryl dyes previously reported and in the present study.

While the binding of styryl dyes to nucleic acids leading to fluorescence enhancement is well known in the literature, most of the work focused on monocationic or dimeric styryl dyes^10,32–39^. In this work, novel dicationic styryl dyes were designed by incorporating additional positively charged sidechain to improve the binding strength with target nucleic acids through the electrostatic interaction with the negatively charged phosphate backbone (Fig. 1). The optical properties of the cationic styryl dyes in the presence and absence of nucleic acid targets were investigated with the aim to find new dyes that can change fluorescence or color in response to various types of nucleic acid targets. Moreover, the structural-properties relationship and binding interaction of dyes and nucleic acids were determined to gain a better understanding of the dye properties and designing of dyes with more desirable characteristics. Applications of the developed styryl dye in a point-of-care platform for colorimetric detection of amplified bacterial genes in food products as well as in cellular imaging were also demonstrated.

## Results and discussion

### Synthesis of cationic styryl dyes

The cationic styryl dye structures were designed to carry a quaternized heteroaromatic core connected to an electron-rich *para*-diethylamino-substituted aromatic ring via a vinylene (-CH=CH-) bridge (Fig. 1). Three heteroaromatic core structures comprising pyridine (PY), benzothiazole (BT) and quinoline (QL) were included in this study. A quaternary ammonium group was attached at the *ortho*-position of the electron-rich aromatic ring *via* a flexible alkoxy linker to provide an additional positive charge that may contribute to the binding to the negatively charged DNA backbone. The dicationic dyes in the PY series [**PY2+(C2)**, **PY2+(C3)** and **PY2+(C4)**] were designed to carry an extra positive charge connected via a linker with different lengths to investigate the effect of the linker length. For comparison of different heteroaromatic cores, the dyes **4QL2+** and **BT2+(NEt**_**2**_**)** were designed to carry the same C4 linker as in **PY2+(C4)**. Furthermore, the dye **BT2+(OMe)** was included to compare the effect of different electron-donating aromatic substituents (-NEt_2_ and -OMe, which are strong and weak electron donors, respectively). The corresponding monocationic dye **PY+**, **4QL+**, and **BT+** without the quaternary ammonium group were also synthesized as reference compounds to evaluate the effect of the additional positive charge on the optical properties of the dyes.

The synthesis of the cationic styryl dyes is outlined in Fig. S1. This required two building blocks: an *N*-methylated heterocyclic ring system carrying an acidic methyl group (*N*-methyl-4-picolinium, *N*-methyl-4-methylquinolinium, or *N*-methyl-2-methylbenzothiazolium iodides) and a substituted benzaldehyde derivative bearing an electron-donating substituent and a positively charged modifier. The starting quaternary ammonium-modified benzaldehydes were prepared by alkylation of the hydroxy groups of 4-(diethylamino)salicylaldehyde and 4-methoxysalicylaldehyde with excess of α,ω-dibromoalkanes (C2, C3, and C4) to afford the corresponding bromoalkylated benzaldehyde derivatives in 18–84% yield. This was followed by another nucleophilic substitution with trimethylamine in THF to obtain the desired quaternized benzaldehyde substrates in good yields (67–100%). The *N*-methylated heteroaromatic compounds were separately obtained by quaternization of the nitrogen atom on the heteroaromatic substrates (4-methylpyridine, 4-methylquinoline, and 2-methylbenzothiazole) with iodomethane by simple heating under neat conditions. These salts were next condensed with the quaternary ammonium benzaldehyde via an aldol-type reaction in refluxing ethanol. This approach provided a simple synthetic route to provide the cationic styryl dyes ranging from low to quantitative yields. The poor yield of the dyes **4QL2+** was likely due to the high pK_a_ of the quinolinium methyl group^40^, leading to the difficult deprotonation. For comparison, the corresponding monocationic dyes (**PY+** and **BT+**) were synthesized according to literature methods^33,41^. The synthesis of the dye **4QL+** required heating the starting materials in acetic anhydride^42^. To facilitate the isolation of the products, the counterions in all cationic dyes were exchanged to hexafluorophosphate (PF_6_^−^) by treatment of the crude product with excess NH_4_PF_6_ in ethanol and the precipitated salts were isolated by filtration. The presence of PF_6_^−^ as the counterion in all dyes was confirmed by ^19^F NMR which showed a doublet signal at − 70 ppm with ^1^*J*_FP_ of 711 Hz, and the MALDI-TOF MS signal at 144.9642 in negative ion mode.

### Optical properties of the synthesized cationic styryl dyes

**Figure 2:**
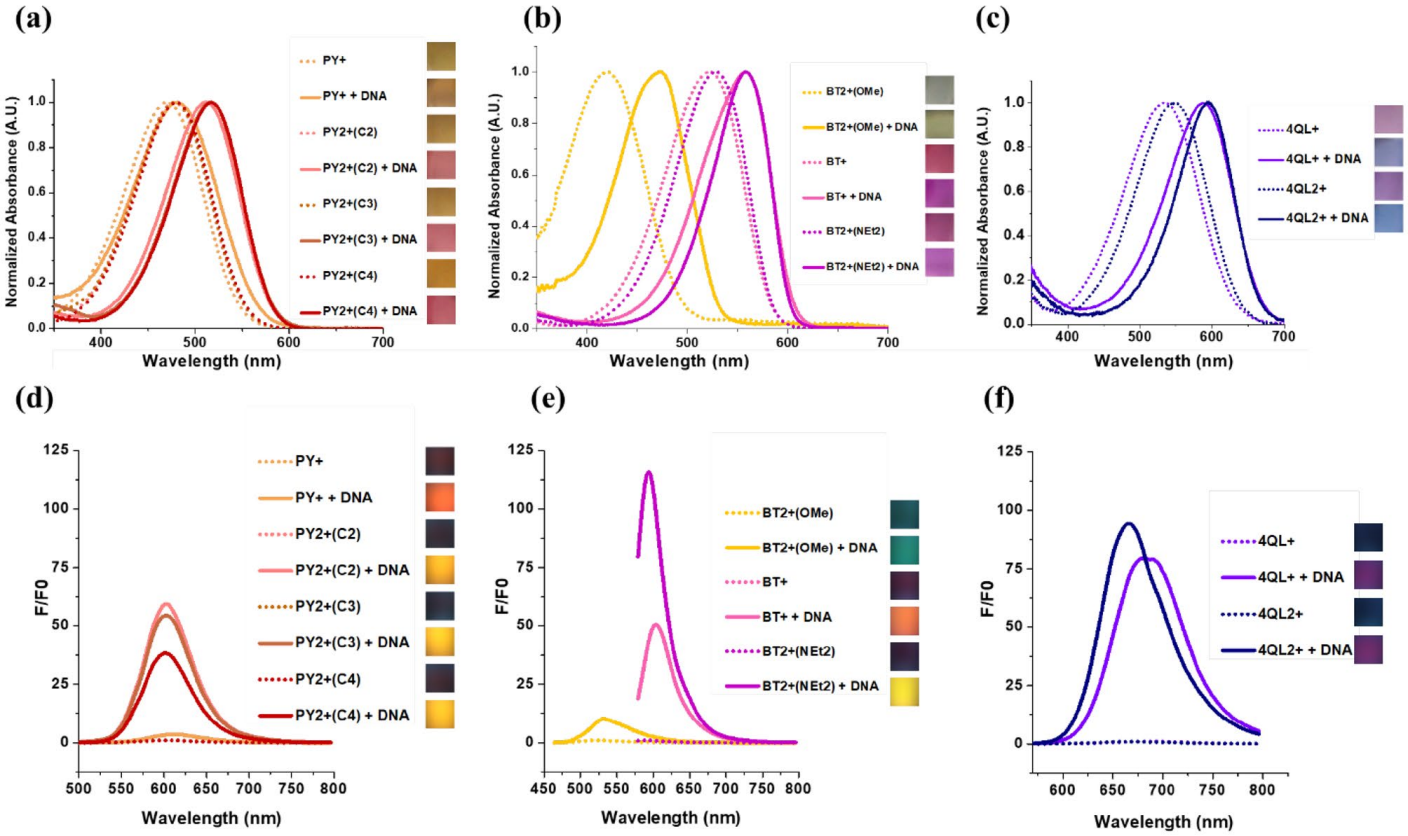
Normalized absorption (**a**–**c**) and fluorescence spectra (**d**–**f**) of cationic styryl dyes in the PY series (**a**,**d**), BT series (b,e), and 4QL series (**c**,**f**) in the absence and presence of DNA. Conditions: [Dye] = 10 μM (or 1 μM for fluorescence studies of **BT + **and **BT2 + (NEt**_**2**_**)**), [DNA(in bp)] = 450 μM (or 45 μM for fluorescence studies of **BT + **and **BT2 + (NEt**_**2**_**)**). Inset: colors of the dyes in daylight (absorption) and under UV illumination (365 nm) in the absence and presence of DNA. Conditions: [Dye] = 50 μM, [DNA (in bp)] = 2,250 μM. All experiments were performed in 10 mM sodium phosphate buffer pH 7.0. Excitation wavelengths **BT2+(OMe)** λ_ex_ = 450 nm, **PY+**, **PY2+(C2)**, **PY2+(C3)**, **PY2+(C4)** λ_ex_ = 480 nm, **BT+ **, **BT2+(NEt**_**2**_**)** λ_ex_ = 565 nm , **4QL+**, **4QL2+ **λ_ex_ = 548 nm.

Due to the extensive conjugation in the structure of the cationic styryl dyes, they strongly absorb in the visible region (470 to 550 nm). Other things being equal, the heteroaromatic part exerts significant effects on the absorption. As the conjugated system gets larger, the absorption maxima shifted toward the longer wavelength (PY < BT < QL). As shown in Fig. 2a–c, the maximum absorption wavelengths of the -NEt_2_ substituted dyes in the absence of DNA were in the range of 471–480, 523–528, and 533–547 nm for PY, BT, and QL series, respectively. The nature of the substituent on the aromatic ring exerts influence on the absorption wavelength, as shown by the substantially blue-shifted absorption maxima of the **BT2+(OMe)** carrying the weakly electron-donating -OMe substituent (OMe) when compared to the **BT2+(NEt**_**2**_**)** carrying the strong electron-donating −NEt_2_ substituent (420 vs 528 nm). The stronger electron-donating substituent facilitates the n → π* transition in the styryl dye, resulting in longer wavelength absorption. The presence of an additional positive charge due to the quaternary ammonium group attached to the ring via the alkoxy liner does not much affect the absorption characteristic of the dye, and generally only slightly red-shifting (less than 10 nm) of the absorption spectra was observed.

In the presence of the double-stranded DNA oligonucleotide, the absorption spectra of all cationic styryl dyes shifted towards the longer wavelength by additional 10–50 nm (Fig. 2a–c). The red-shifting in the absorption maxima of similar dyes relating to the solvent polarity has been well-recognized^43^. Due to the most pronounced absorption change of **4QL2+** in the presence of DNA (up to 48 nm red-shifting), this dye was used as a model for further studies of the color-changing behavior. The absorption and fluorescence spectra of **4QL2+** were measured in various solvents including water, ethyl acetate, acetone, dimethyl sulfoxide, ethanol, methanol, dichloromethane, acetonitrile, and glycerol. It was clearly observed that the λ_max_ changed according to the solvent polarities—being more red-shifted in less polar solvents (Fig. S2a). It should be noted that the shape and λ_max_ of the absorption spectra of the **4QL2+** in aqueous solution at various concentrations remained constant over the concentration range of 1–50 μM (Fig. S2c). The results indicated that the color change was not associated with the change in the dye aggregation states under the measurement conditions.

From the relationship between absorption energies and E_T_(30) of each solvent (Fig. S2b), the trend showed that absorption energies were increase along the increasing E_T_(30) of the solvent. This trend is consistent with the ground state of the styryl dyes being more polar than the excited state^44^. Due to the extensive delocalization as a result of an intramolecular charge transfer, the dye molecule in the ground state was better stabilized in more polar solvents, leading to a larger energy gap between the ground state and the excited state. Thus, the electronic transition occurred at shorter wavelength, resulting in the observed hypsochromic shift with increasing solvent polarities (Fig. S5). The results observed are consistent with typical behavior of donor–acceptor dyes^45^. Accordingly, the red-shifting of the absorption maxima of **4QL2+** was attributed to the change in the local polarity of the free dye (polar environment) and DNA-bound dye (nonpolar environment). Other dyes in this study also exhibited the same trend in the absorption spectra change, although the changes are less pronounced (Δ λ_abs_ = 11–37 nm for PY series and 30–35 nm for BT series).

According to the fluorescence spectra (Fig. 2d–f), the free styryl dyes showed low fluorescence quantum yields (*Φ*_F_ = 0.001–0.004). The fluorescence quantum yields increased between 3.5 and 126 folds in the presence of excess DNA targets (Table 1, Fig. 2d–f). In all cases, the dicationic dyes consistently showed much larger enhanced fluorescence when compared to the monocationic dyes in the same series. The substantial increase in the fluorescence quantum yields of similar benzothiazole-based^36^ and pyridine-based styryl dyes^46^ have been explained in terms of the restricted motion of the dye molecules in the excited state, thus inhibiting the radiationless relaxation pathways^47^. To determine whether the same effect is in operation with the quinoline dye family, the fluorescence spectra of the **4QL2+** dye were measured in aqueous solution containing glycerol both in the absence and presence of DNA (Fig. S3a–b). The results showed that fluorescence intensities of the dye increased in the presence of glycerol due to the increased viscosity of the solution. Moreover, the emission maxima and the fluorescence intensity of the dye in glycerol and the DNA-bound dye in water were quite similar. In addition, the dye in glycerol showed no significant change in fluorescence spectra in the presence of DNA. Absorption spectra of **4QL2+** in glycerol in the absence and presence of DNA (Fig. S3c) also almost identical spectra with the same maximum absorption wavelength (580 nm). The results confirm that the restriction of the conformational freedom of the **4QL2+** dye upon binding to DNA is responsible for the fluorescence increase in the presence of DNA similar to other styryl dyes reported in the literature^48^. To further confirm the effect of molecular motion to the fluorescence intensity, the fluorescence of the **4QL2+** dye in aqueous glycerol solution was further measured at different temperatures. The sample was equilibrated at 0 °C followed by heating to 30, 60 and 90 °C before cooling down to 0 °C again with the same temperature program and the fluorescence spectra were recorded at each temperature. The strong fluorescence emission at low temperature decreases as the temperature increases in a reversible manner (Fig. S3d). The results are fully consistent with the mechanism of fluorescence enhancement based on decrease molecular motion as proposed above.

**Table 1:** Optical properties of cationic styryl dyes in the absence and presence of dsDNA: absorption maxima λ_max_(abs), extinction coefficients (ε), emission maxima λ_max_(em), fluorescence quantum yield (*Φ*_F_) and fluorescence enhancement ratio (*F*/*F*_0_).

Dye	ε^a^ (× 10^4^ M^−1^ cm^−1^)	λ_max_ (nm)				*F*/*F*_0_^b^		*Φ*_F_^c^		Brightness^d^ (× 10^3^)		*Φ*_F (DNA)/_*Φ*_F (dye)_
		abs (dye)	abs (dye + DNA)	em (dye)	em (dye + DNA)	Value	Wavelength (nm)	Dye	+ DNA	Dye	+ DNA	
**PY+**	5.0	471	482	612	612	3.5	600	0.002	0.008	0.1	0.4	3
**PY2+(C2)**	5.2	478	511	605	603	60.5	600	0.002	0.114	0.1	5.9	66
**PY2+(C3)**	4.3	479	516	599	603	54.8	600	0.002	0.133	0.09	5.7	71
**PY2+(C4)**	5.4	480	517	603	600	38.5	600	0.002	0.127	0.1	6.8	72
**BT+**	3.3	523	558	595	605	56.6	600	0.004	0.053	0.1	1.7	14
**BT2+(NEt**_**2**_**)**	2.8	528	558	591	595	126	600	0.004	0.371	0.1	1.0	88
**BT2+(OMe)**	1.6	420	473	521	531	10.9	530	0.004	0.014	0.06	0.2	3
**4QL+**	2.2	533	587	680	682	80.1	670	0.001	0.038	0.2	0.8	36
**4QL2+**	2.5	547	595	665	670	98.4	670	0.001	0.057	0.2	1.4	86

Conditions: [Dye] = 2 μM, [DNA] = 1 μM; [DNA(in bp)] : [Dye] = 15 : 1; all measurements were performed in 10 mM sodium phosphate buffer pH 7.0. dsDNA = 5′-CGCGGCGTACAGTGATCTACCATGCCCTGG-3′ + 3′-GCGCCGCATGTCACTAGATGGTACGGGACC-5′.

^a^In MeOH ([Dye] = 0–10 μM).

^b^Fluorescence intensity with dsDNA (*F*) and without dsDNA (*F*_0_).

^c^The following reference dyes were used: Rhodamine 6G (*Φ*_F_ = 0.95, λ_ex_ = 470–510 nm) for PY series, **BT+ **and **BT2+ (NEt**_**2**_**)**; Fluorescein (*Φ*_F_ = 0.95, λ_ex_ = 470–490 nm) for **BT2+(OMe)**; cresyl violet (*Φ*_F_ = 0.54, λ_ex_ = 540–590 nm) for **4QL+ **and **4QL2+.**

^d^Brightness = ε × *Φ*_F_.

The fluorescence spectra of **4QL2+** as the representative dye was also studied in various organic solvents. Similar to the absorbance, the Stokes shifts of the dye **4QL2+** showed a strong dependency to solvent polarity. The Lippert-Mataga plot^49^ revealed a positive relationship between the Stokes shift and orientation polarizability of the solvents surrounding the dye molecules (Fig. S4c), which suggests that solvents affect molecular dipole moment change between ground and excited state of dye. Furthermore, the results showed that in addition to the solvent viscosity, polarity also affect the fluorescence properties of **4QL2+** (Fig. S4a) both in terms of fluorescence emission maxima and intensities. This trend can be also utilized to describe optical properties of other dyes in this study with regard to their fluorescence enhancement mechanism.

As noted earlier, the dyes **BT2+(OMe)** and **BT2+(NEt**_**2**_**)** which shared the same benzothiazolium core structure but carried different electron donating substituents on the aromatic ring (-OMe and -NEt_2_, respectively) showed quite different optical characteristics (Table 1). Since the -OMe group is a weaker electron donating substituent, the absorption occurred at a shorter wavelength. In the presence of DNA, the maximum absorption was shifted from near UV (420 nm) to visible region (473 nm). In terms of fluorescence behavior, relatively poor response towards DNA was observed (10.9 folds intensity enhancement). For comparison, the **BT2+(NEt**_**2**_**)** showed 125.7 folds fluorescent increase in the presence of DNA.

According to the results from this study, both the heteroaromatic moieties (PY, BT, and QL) and the substituents on electron-rich aromatic part (− NEt_2_ and − OMe) were found to significantly affect dye’s absorption and fluorescence characteristics. The results suggest that the optical properties of the styryl dye is conveniently tunable by changing the heteroaromatic core and the substituent on the aromatic ring. Meanwhile, the additional quaternary ammonium group attached via a non-conjugated alkoxy linker showed small effect on the dyes’ optical properties while greatly enhancing the responsiveness of the dyes towards DNA. It should be noted that the dyes exhibit high selectivity towards DNA. No significant change in the absorption spectra with small fluorescence enhancement was observed in the presence of bovine serum albumin (BSA) as shown in Fig. S6. On the other hand, some small optical responses were observed with sodium polystyrylsulfonate (PSS) which contains negative charge and aromatic moiety similar to DNA.

### Comparison of DNA binding affinity between monocationic dyes and dicationic dyes

The optical properties of the dicationic dyes **PY2+(C4)**, **4QL2+**, and **BT2+(NEt**_**2**_**)** in the absence and presence of DNA were compared to those of monocationic dyes **PY+**, **4QL+**, and **BT+** to examine the effect of the additional positive charges in the dye molecules in enhancing the DNA binding and photophysical properties. As shown in Fig. S16, UV and fluorescence spectra revealed that the dicationic styryl dyes was more responsive to DNA in terms of both absorption and fluorescence changes when compared to the monocationic dyes in the same series. The dyes **PY+** and **PY2+(C4)** pair underwent the most significant changes, followed by the **BT+** and **BT2+(NEt**_**2**_**)** pair. The dyes **4QL+** and **4QL2+** showed comparatively small but still observable changes. When compared in the presence and absence of DNA, the dye **BT2+(NEt**_**2**_**)** showed the largest fluorescence response (125.7 folds), while the dye **4QL2+** showed the largest absorption change (48 nm). The study indicated that introduction of the additional positive charges to the dye molecules improves the dye’s response to DNA. Therefore, these dicationic dyes should be more suitable for DNA sensing than the corresponding monocationic dyes that have already been extensively used as DNA stains^33,50,51^.

The binding constant (*K*_b_) is a useful parameter to describe the binding affinity between DNA and their ligands. The binding constants were determined from the fluorescence titration curves in the linear ranges determining from Fig. S16 by modified McGhee and von Hippel equation (Eq. 5) (Fig. S17^52^). Intercalators that bind to DNA usually have binding constants (*K*_b_) of less than 10^7^ M^−1^^52^. Acridine orange (AO), thiazole orange (TO)^53^, and ethidium bromide^54^, have the *K*_b_ values of 5.0 × 10^4^, 1 × 10^6^, and 1.5 × 10^5^ M^−1^, respectively. Another parameter obtained from the equation is binding site (*n*), which describes base pair(s) required for ligand molecule to occupy. From the results in Table 2, the small *n* values of **BT+**, **BT2+(NEt**_**2**_**)**, **4QL+** and **4QL2+** indicate that the dye is more likely to interact with DNA via intercalation. The larger *n* values of **PY+** (4.4), **PY2+(C4)** (4.0) are more consistent with the groove-binding mode in which at least 3 base pairs would be required for the dye to occupy^52^. Consistent with the expectation, the larger binding constant of **BT2+(NEt**_**2**_**)** than **BT+** by an order of magnitude also confirms that the introduction of an additional positive charge improves the binding affinity of the dye towards DNA targets as proposed. The same conclusion is also true for the **4QL+**/**4QL2+** and **PY+**/**PY2+** pairs as well. Thus, the experiments clearly support the original proposal that the introduction of the positive charge to the styryl dye could improve its DNA binding, which was reflected in the larger binding constants and better responsiveness of the dicationic dyes compared to the monocationic dyes. The acquired binding parameters of **BT+** (*K*_b_ = 3.5 × 10^4^ M^−1^, n = 2.5) were in accordance with anther related dye that carry -NMe_2_ group as the aromatic substituent in which the *K*_b_ = 1.8 × 10^4^ M^−1^ and n = 3.4 were reported^52^.

**Table 2:** Binding constant (*K*_b_) and the number of dsDNA base pairs occupied by one bound dye molecule (*n*) values of the synthesized styryl dyes.

Dye	*K*_b_ (× 10^4^ M^−1^)	*n*
**PY+**	1.0	4.4
**PY2+(C4)**	14.1	4.0
**BT+**	3.5	2.5
**BT2+(NEt**_**2**_**)**	12.1	2.5
**4QL+**	7.0	2.8
**4QL2+**	24.5	2.1

Conditions: [Dye] = 2 μM, [DNA (in bp)] = 0.3–3 μM; all measurements were performed in 10 mM sodium phosphate buffer pH 7.0.

The modes of binding of selected dyes towards double-stranded DNA were further confirmed by their ability/inability to displace EtBr and DAPI from DNA duplexes. Ethidium bromide (EtBr) is a well-known DNA intercalator which was widely used as a DNA fluorescent staining agent. The displacement of EtBr from DNA by a small ligand indicates that the ligand can act as an intercalator^55^. On the other hand, 4’,6-diamidino-2-phenylindole (DAPI) is a common minor groove binder and other groove binder molecules are able to displace DAPI from the DAPI-DNA complex^2^. The DNA models used in this experiment consist of d(AT)_10_ and d(GC)_10_ to ensure a homogeneous binding mode. In general, EtBr can intercalate to both AT-rich and GC-rich DNAs^56^, while DAPI could only bind to minor grooves of AT-rich DNA^57^. As shown in Fig. S25, both EtBr and DAPI molecules were displaced from DNA strands by **4QL2+**, which suggest that this dye can act as both an intercalator and a groove binder. Likewise, the results in Figs. S19–S24 suggested that the dyes **4QL+**, **BT+**, **BT2+(NEt**_**2**_**)** and **BT2+(OMe)** can act as both intercalators and groove binders. These results are consistent with reported literature which proposed that benzothiazolium-based styryl dyes have both intercalating and groove binding characters based on binding parameters obtained from the McGhee and von Hippel equation^52^. On the other hand, the pyridinium-based dyes **PY+** and **PY2+(C4)** can only act as groove binders, which is also in agreement with the literature^58^.

### Ionic strength dependence of the dye-DNA binding

**Figure 3:**
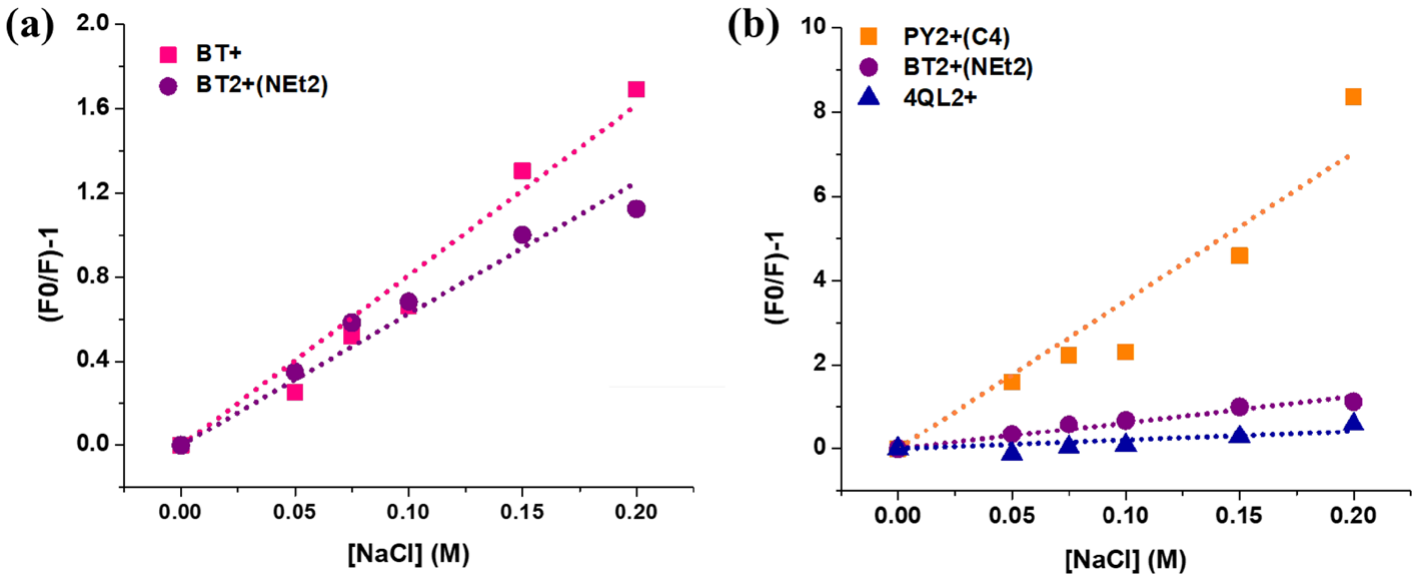
Quenching of the complexes of **BT + **and **BT2 + (NEt**_**2**_**)** (**a**), **PY2 + (C4)**, **BT2 + (NEt**_**2**_**)**, and **4QL2 + **(**b**) (1 µM) with DNA (15 µM, in bp) in the presence of NaCl (50–200 mM).

As the cationic styryl dyes carries the positive charge(s) and DNA has a negatively charged phosphate backbone, the electrostatic interaction between the dyes and the DNA backbone should play a crucial role in the binding interaction. The ionic strength dependence of dye-DNA binding was determined in the presence of NaCl as a strong electrolyte. The increase in ionic strength leads to a decrease in the repulsive electrostatic interactions between backbone phosphate groups, which in turn results in a stabilization DNA duplexes^59,60^. However, the increase in the salt concentration interferes with the binding of the positively charged dye with the DNA double helix^2^. Due to the most pronounced fluorescence change in the presence of DNA, the dyes **BT+** and **BT2+(NEt**_**2**_**)** were selected to investigate the effect of ionic strength on the interaction of mono- and dicationic dyes with DNA in this study. The fluorescent intensity data obtained from the microplate reader was analyzed following the Stern-Volmer equation (Eq. 4)^58^. The observed quenching constants (*K*_SV_) were 8.09 and 6.26 M^−1^ for the dye **BT+** and **BT2+(NEt**_**2**_**)**, respectively (Fig. 3a). The results revealed that the introduction of an additional positive charge to the dye molecules enhances the binding affinity with the DNA, leading to a more stable dye-DNA complex. The effect of ionic strength towards the DNA binding of the dye **BT2+(NEt**_**2**_**)** as well as two other dicationic dyes, namely **PY2+(C4)** and **4QL2+**, were also investigated at a higher NaCl concentrations. In the presence of NaCl (50–200 mM), the observed quenching constants (*K*_SV_) were 35.3, 6.3 and 2.0 M^−1^ for the dyes **PY2+(C4)**, **BT2+(NEt**_**2**_**)**, and **4QL2+**, respectively (Fig. 3b). The results can give some insights into the mode of binding of the dye-DNA complexes that are in good agreement with the fluorescent indicator displacement assay results. Since the intercalation of the dye molecules between the DNA base stacks effectively protects the entrapped dye molecules, the fluorescence of the intercalated dye is not much affected^58^. In contrast, the dye **PY2+(C4)** showed an order of magnitude larger *K*_SV_ value which is consistent with the fact that this dye only bind to the groove thus making it more susceptible to the displacement and quenching. The findings are in line with previous literature^58^, which showed that a pyridinium-based styryl dye related to **PY+** called DASPMI is a DNA groove binder with *K*_SV _= 16.8 M^−1^ at an NaCl concentration range of 10–300 mM.

### Effect of the length of the linker joining the positive charge and the aromatic ring

The fluorescence and absorption properties of the monocationic dye **PY+** and the dicationic dyes bearing different linkers **PY2+(C2)**, **PY2+(C3)**, and **PY2+(C4)** were studied to investigate the effect of the length of the linker joining the positively charged quaternary ammonium group and the aromatic ring (Fig. S18b). In the presence of dsDNA, the **PY2+(C2)** and **PY2+(C3)** dyes exhibited the largest fluorescence response. The **PY2+(C4)** dye showed a somewhat lower fluorescence response, but it was still significantly larger than the control **PY+** dye. The findings reveal that the introduction of an additional positive charge improves the dye’s fluorescence response to DNA, and that the shorter linker length appeared to improve the fluorescence response better. Nevertheless, the effect of the linker length was much less pronounced when compared to the effect of the introduction of additional positive charge.

The results from the UV spectra in Fig. S18a did not precisely follow the same trend from the fluorescence spectra. In the presence of dsDNA, the dyes **PY2+(C3)** and **PY2+(C4)** showed the most red-shifted absorption spectra, followed by the dye **PY2+(C2)**. However, all dicationic dyes showed a much more distinct absorption change compared to the monocationic dye **PY+**. Consequently, the linker length did not significantly affect the dyes’ response towards DNA for both fluorescence and colorimetric assays.

### Docking simulation for DNA-dye interaction study

To further investigate the binding affinity improvement of dicationic styryl dyes toward DNA targets and their mode of binding, molecular dockings of four styryl dyes bearing -NEt_2_ substituent (**BT+**, **BT2+(NEt**_**2**_**)**, **PY2+(C4)** and **4QL2+**) were performed with AutoDock 4.2.6^61^. Due to the increase of fluorescence intensity upon binding on DNA, the dihedral angle between electron-rich aromatic and heteroaromatic moieties was constrained to 0° to maximize their ICT via donor-acceptor conjugation.

**Figure 4:**
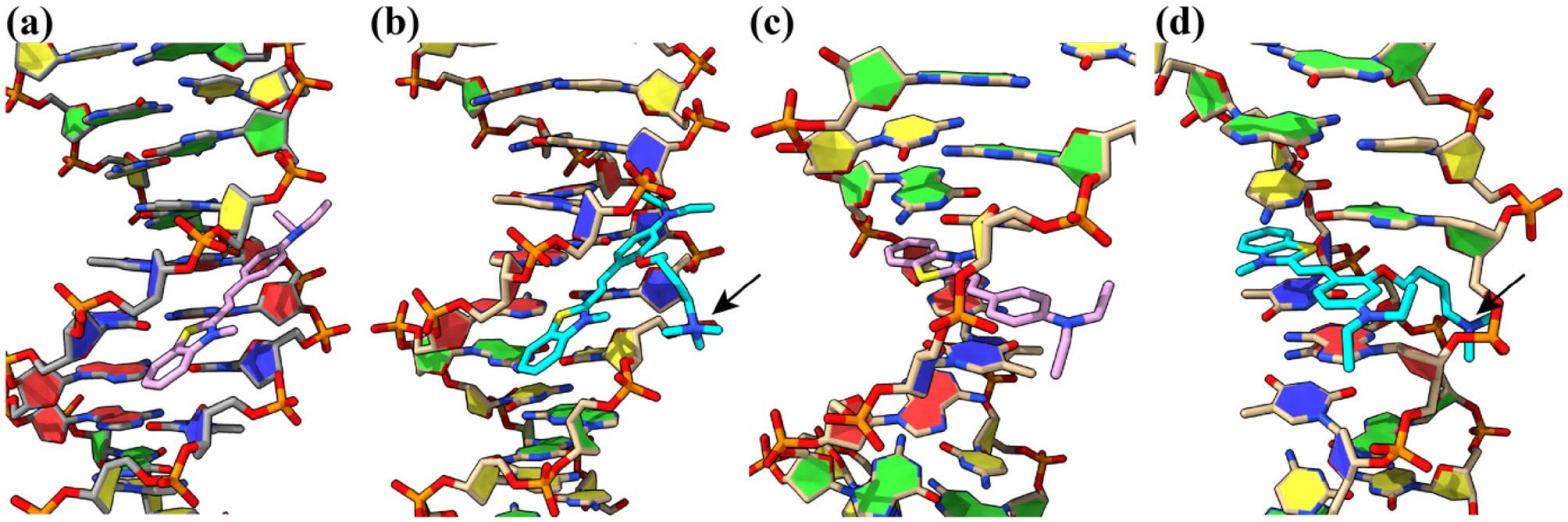
Molecular docking of (**a**) **BT + **, (**b**) **BT2 + (NEt**_**2**_**)** on a minor-groove DNA (4C64) and (**c**) **BT + **, (**d**) **BT2 + (NEt**_**2**_**)** at an intercalative site of DNA (108D). Black arrows indicate the electrostatic interaction of quaternary ammonium side chain to the phosphate backbone.

In case of minor groove interaction, a crystal structure of the Drew-Dickerson DNA duplex (4C64)^62^ was used as a representative model. Comparison of binding energies between monocationic (**BT+**; − 9.9 kcal/mol) and dicationic dyes (**BT2+(NEt**_**2**_**)**; − 11.4 kcal/mol) supported the important electrostatic interaction of quaternary amine side chain to the phosphate backbone (Fig. 4a,b). Among the dicationic dyes, **BT2+(NEt**_2_**)** and **PY2+(C4)** preferably bound at AT nucleobases, whereas the binding location of **4QL2+** slightly shifted toward CG to accommodate the large quinoline ring (Fig. S26). Also, the binding energy of **BT2+(NEt**_**2**_**)** (− 11.4 kcal/mol) was slightly more negative than **4QL2+** (− 11.3 kcal/mol) and **PY2+** (− 10.3 kcal/mol) possibly due to the appropriate position of the positively-charged benzothiazole to the phosphate backbone. In all cases, the positively-charged side chain can interact the phosphate backbone which increase the binding energies of these dicationic dyes.

For intercalative binding mode, an average NMR solution structure of a DNA complex with a dimeric intercalator dye TOTO (108D) was selected because of the close structural similarity between styryl dyes and thiazole orange^63^. Similar to the minor groove interaction, binding energy of **BT2+(NEt**_**2**_**)** was more negative than **BT+** by approximately 1.6 kcal/mol (− 9.0 vs − 7.4 kcal/mol; Fig. 4c,d). All three dicationic dyes fit into the intercalating site and their charged side chains interacted with phosphate backbone similar to the minor-groove model (Fig. S27). The dye **4QL2+** (− 9.5 kcal/mol) had a slightly better binding energy than **BT2+(NEt**_**2**_**)** (− 9.0 kcal/mol) and **PY2+(C4)** (− 8.4 kcal/mol) possibly due to the favorable π–π stacking of quinoline to the nucleobases.

Overall, styryl dyes have more favorable to the minor-groove interaction by ~ 2 kcal/mol, which support the previous experimental data that **PY2+(C4)** acts as a groove binder. However, high binding energies of **BT2+(NEt**_**2**_**)** and **4QL2+** in both minor groove and intercalative models also suggest that these dyes can be both groove binders and intercalators. The more negative binding energies of dicationic styryl dyes consistent with the previous experiments that the addition of quaternary ammonium side chain enhances the binding affinity toward DNA duplex.

### Applications in colorimetric DNA detection and cellular nucleic acids imaging

The excellent colorimetric and fluorescence responses of the dicationic styryl dyes suggest its obvious application in nucleic acid detection. To evaluate the performance of the cationic styryl dyes for this purpose, their responsiveness towards dsDNA in the low concentration ranges was next investigated. For the fluorescence method, the maximum fluorescence intensities of the dye at a fixed concentration and at least five DNA concentrations were used to construct the calibration curve. For the absorption method, the A_f_/A_i_ ratios, which expressed relative change of maximum absorption as determined from the absorption spectra of the dye in the presence of excess DNA, at a fixed dye concentration and various concentrations of DNA were used. Based on the (3 × SD)/slope of the calibration curve, the limits of detection (LODs) were determined as shown in Table 3. All dicationic dyes showed lower LODs than the corresponding monocationic styryl dyes with the same heteroaromatic core structures. When compared to the existing commercially DNA fluorescent staining dyes^21^, the most responsive dicationic styryl dye **BT2+(NEt**_**2**_**)** exhibited higher sensitivity than ethidium bromide (10–33 ng/mL)^64,65^, Hoechst 33258 (10 ng/mL)^66^, and 4-Di-1-ASP (5 ng/mL)^21^. Moreover, the sensitivity of **BT2+(NEt**_**2**_**)** was comparable with the bis-cyanine dyes including TOTO and YOYO (0.5 ng/mL), which revealed the equivalent potential of our developed dye with dimeric molecules. An application of **BT2+(NMe**_**2**_**)** for the fluorescence detection of amplified bacterial DNA have been recently demonstrated by our group^67^.

**Table 3:** Comparison of the limit of detection of cationic styryl dyes for the determination of dsDNA by fluorescence and UV–visible spectrophotometry.

Dyes	Detection method			
	Fluorescence		Absorption	
	λ_ex_ (nm)/λ_em_ (nm)	LOD (ng/mL)	A_f_ (nm)/A_i_ (nm)	LOD (ng/mL)
**PY+** **PY2+(C4)**	480/600 480/600	276 (15 nM) 35 (1.9 nM)	520/480 520/480	5918 (321 nM) 583 (32 nM)
**BT+** **BT2+(NEt**_**2**_**)**	565/600 565/600	4.1 (0.22 nM) 1.2 (0.07 nM)	560/530 560/530	3213 (174 nM) 973 (53 nM)
**4QL+** **4QL2+**	548/670 548/670	57 (3.1 nM) 50 (2.7 nM)	600/550 600/550	1666 (90 nM) 433 (23.5 nM)

Conditions: [Dye] = 2 μM, [DNA (in bp)] = 0.3–3 μM; all measurements were performed in 10 mM sodium phosphate buffer pH 7.0.

In terms of colorimetric detection of dsDNA, much higher LODs (lower sensitivity) than the fluorescence detection for all cationic styryl dyes studied (Table 3). However, the simplicity of the colorimetric assay may offer alternative choices for the point-of-care DNA detection that are more convenient to perform than the fluorescence assay. To date, there are only limited collection of commercially available colorimetric dyes for DNA assays. When compared to the leuco-triphenylmethane dyes (LCV) that has recently been introduced as a colorimetric dye for the detection of DNA with a relatively high LOD of 7100 ng/mL^29^, the dicationic **PY2+(C4)**, **BT2+(NEt**_**2**_**)**, and **4QL2+** showed far better LODs thus offering more sensitive colorimetric DNA detection (LODs in the range of 400–1000 ng/mL). Moreover, the colorimetric DNA detection limits of the dicationic dyes were better than the corresponding monocationic dye and were comparable with unmodified gold nanoparticle-PNA platform (50 nM)^24^. The dye **4QL2+** which showed the most pronounced spectral shift in the presence of dsDNA was the most sensitive dye for the dsDNA assay, giving the LOD of 23.5 nM. These results suggest that the introduction of the additional positive charge to the dye molecule improved the DNA detection sensitivity in both the fluorescence and colorimetric assays. Thus, these new dicationic styryl dyes are suitable for the fluorescence and colorimetric detection of nucleic acid targets in various applications.

**Figure 5:**
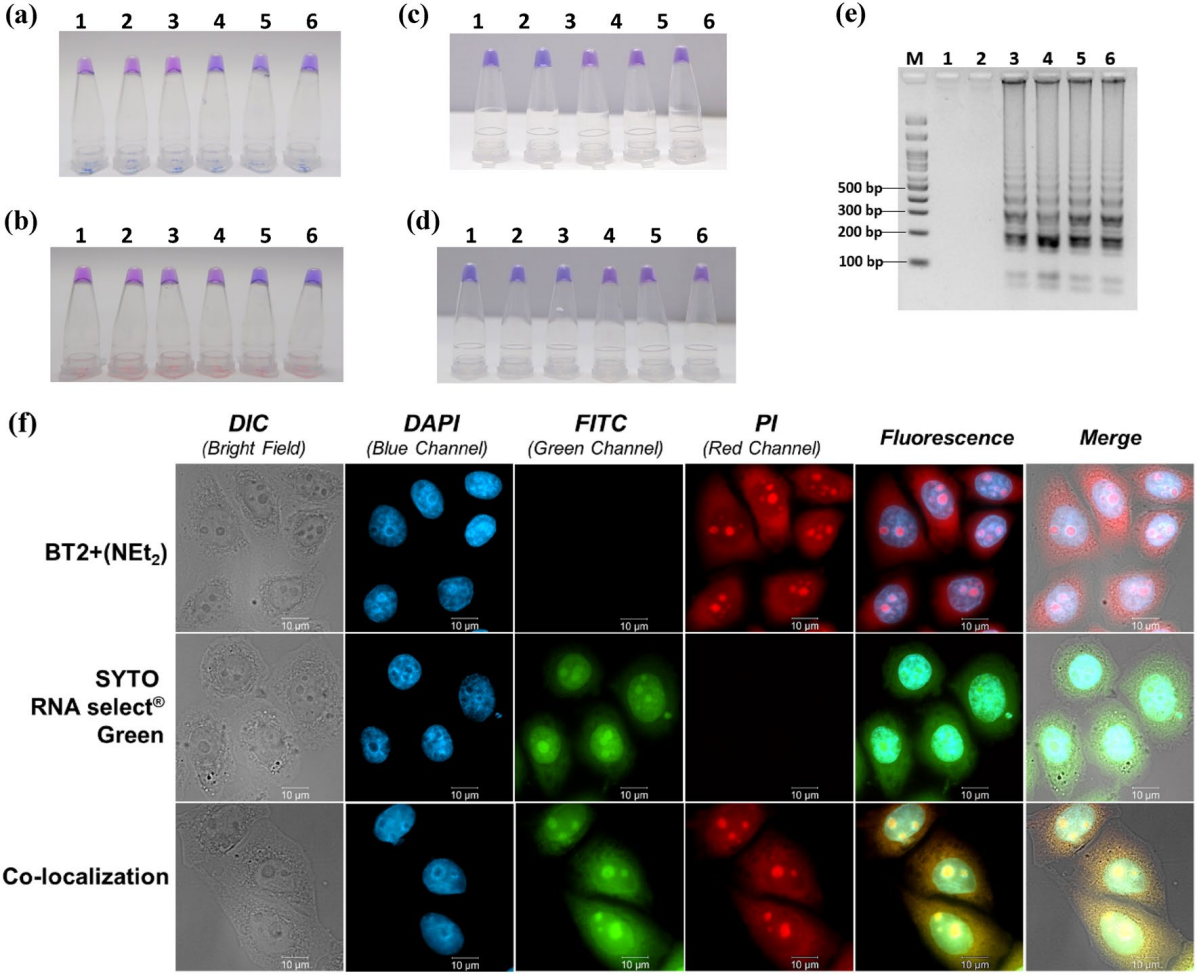
Applications of cationic styryl dyes for DNA detection in vitro (**a**–**e**) and for cellular nucleic acid detection (**f**). Sensitivity (**a**,**b**) and specificity (**c**,**d**) test of *E. coli* (**a**,**c**) and *S. aureus* (**b**,**d**) detection based on LAMP-**4QL2+ **assay, and gel electrophoresis results from the *E. coli* sensitivity assay (**e**). Lanes in (**a**), (**e**) **1**: negative control, **2**: 3.39 × 10^−1^, **3**: 3.39 × 10^0^, **4**: 3.39 × 10^1^, **5**: 3.39 × 10^2^, **6**: 3.39 × 10^3^ copies of DNA/reaction; (**b**) **1**: negative control, **2**: 3.3 × 10^−2^, **3**: 3.3 × 10^−1^, **4**: 3.3 × 10^0^, **5**: 3.3 × 10^1^, **6**: 3.3 × 10^2^ copies of DNA/reaction; (**c**) **1**: *E. coli* ATCC 25922, **2**: *E. coli* MCCU0349, **3**: *E. cloacae*, **4**: *S. aureus* ATCC 25923, **5**: *S. epidermidis* ATCC 12228, d) **1**: *S. aureus* ATCC 25923, **2**: *S. aureus* MCCU0357, **3**: *S. aureus* MCCU0370 sample; **4**: *S. epidermidis* ATCC 12228, **5**: *S. saprophyticus* ATCC 15305, **6**: *E. coli* ATCC 25922. (**f**) Fluorescence images of HeLa cells stained with **BT2+(NEt**_**2**_**)** and SYTO RNA select® Green (as individual dyes or co-stained). The cells were co-stained with DAPI in all cases (blue channel). Scale bars = 10 µm.

Next, the use of dicationic styryl dye **4QL2+** which provided pronounced colorimetric change from pink to blue in the presence of dsDNA for the colorimetric detection of bacterial DNA (*E. coli* and *S. aureus*) in vitro was demonstrated. The bacterial DNA was first amplified by the loop-mediated isothermal amplification (LAMP)^68^, which offers a simple, specific, and highly efficient nucleic acids amplification without the need for complicated instruments, followed by addition of the dye. Two sets of LAMP primers were designed for the amplification of *E. coli* and *S. aureus* DNA. The results in Fig. 5a–d showed that the LAMP-**4QL2+** assay provided positive results (blue color) only in the samples that contain the correct bacterial DNA samples. The lowest amounts of DNA targets that were successfully detected by naked eyes were approximately 33–34 copies of DNA for both species. This was an order of magnitude higher than gel electrophoresis which could detect down to 3.4 copies of DNA (Fig. 5e). However, the level of sensitivity offered by the LAMP-**4QL2+** was comparable to LAMP-hydroxynaphthol blue (HNB)^69^ and standard PCR methods, which were widely used for this purpose. Thus, the LAMP-**4QL2+** platform offers highly specific naked-eyes detection method for LAMP products detection.

Finally, to demonstrate the applicability of the dicationic styryl dyes for cellular imaging, HeLa cells were stained with the dye **BT2+(NEt**_**2**_**)** which showed outstanding fluorescent response towards nucleic acids *in vitro*, and because it exhibited less cytotoxicity compared to its monocationic counterpart, **BT+**. As confirmed by MTT assay, the cell viability was not affected following treatments with the dye at a concentration up to 25 µM for 24 h (Fig. S28). Co-staining with DAPI, which is commonly used to selectively stain cellular nuclei^70^, was performed in all cell imaging experiments (Fig. 5f, second column). The **BT2+(NEt**_**2**_**)** dye stained several parts of the whole cell, resulting in a high fluorescent signal all over the cells, but the brightest area appeared in the nucleoli regions (Fig. 5f, top row), which serve as the site for ribosome synthesis and RNA assembly^71^. The ability to stain nucleoli and chromosomes of the monocationic dye **BT+** has recently been reported^33^. Based on the structural similarity between **BT+** and **BT2+(NEt**_**2**_**)**, we reasoned that **BT2+(NEt**_**2**_**)** might bind to the same target as the **BT+** dye. To verify this hypothesis, a colocalization experiment with SYTO RNA select^®^ Green—an RNA selective dye which displays significantly higher fluorescence intensity when complexed with RNA compared to DNA—was performed for comparison. The results in Fig. 5f showed similar cell staining patterns between SYTO RNA select^®^ Green and **BT2+(NEt**_**2**_**)**, as confirmed by fluorescence image analysis (Fig. S29). The Pearson’s coefficient of **BT2+(NEt**_**2**_**)** and SYTO RNA select^®^ Green as 0.638 is also showed good co-localization. Furthermore, **BT2+(NEt**_**2**_**)** staining showed higher fluorescence intensity in the nucleoli than that in other parts of nuclei. Although the RNA binding behavior of **BT2+(NEt**_**2**_**)** is currently under investigation, these results suggested that the dye may bind to secondary structures of rRNA that are partly double-stranded. Other parts of the nuclei were not stained by this dye. Moreover, the ability of **BT2+(NEt**_**2**_**)** to stain other locations in the cells suggests that it may also bind to other kinds of cellular nucleic acids including mitochondrial DNA, ribosomal RNA, other cytoplasmic nucleic acids, and nucleic acids in the nucleus. In a more recent study on a series of dyes related to **4QL+**, it was reported that the histones forming the nucleosomes on cellular DNA hindered the dye binding, thus resulting in a lower fluorescent response^10^. Collectively, these results indicated that **BT2+(NEt**_**2**_**)** can stain several cellular nucleic acids, especially for RNA in the nucleoli in live cells. This could potentially be applied to study sub-cellular compartments within the cell.

In summary, this study introduces new dicationic styryl dyes with the pyridinium, benzothiazolium, and quinolinium heteroaromatic cores with the aim to improve the binding interaction with the negatively charged backbone of nucleic acids. The additional positive charge was introduced to the dye molecules via a quaternary ammonium group attached to the electron-rich aromatic ring via a flexible alkoxy linker. The optical properties and DNA binding interaction of the dicationic dyes were compared with the corresponding monocationic styryl dyes. In the presence of dsDNA, a bathochromic shift of the absorption spectra (up to 48 nm for **4QL2+**), and fluorescence enhancement (up to 126 folds for **BT2+(NEt**_**2**_**)**), with high selectivity for DNA. The results revealed that the dicationic dyes consistently provided higher affinity towards dsDNA and more pronounced fluorescence and colorimetric responses than the monocationic dyes. The linker length joining the positive charge to aromatic ring exerted relatively small effects to the responsiveness of the dyes towards DNA when compared to the extra positive charge. The small binding site (*n*) and displacement titration suggest that the benzothiazolium- and quinolinium-based styryl dyes may bind to DNA as groove binders and intercalators, while the pyridinium-based styryl dyes only act as a groove binder. These dicationic dyes showed an improved sensitivity in the detection of DNA, with detection limits (LODs) in the range of 1.2–50 ng/mL in fluorescence mode, and 400–1000 ng/mL in colorimetric mode. These values compare favorably to the existing commercial dyes. The applications of the dicationic styryl dyes for the detection of bacterial DNA in vitro as a prototype for a point-of-care platform for foodborne pathogen detection and cellular nucleic acids imaging were also satisfactorily demonstrated.

## Methods

### General

All reagent grade chemicals and solvents were purchased from standard suppliers and were used as received without further purification. Oligonucleotides were purchased from BioDesign or Pacific Science (Thailand). MilliQ water was obtained from an ultrapure water system fitted with a Millipak® 40 filter unit (0.22 μ) was used in all experiments. ^1^H and ^13^C NMR spectra were recorded in a suitable deuterated solvent on JEOL JNM-ECZ500R/S1 operating at 500 MHz (^1^H) and 126 MHz (^13^C). ^19^F NMR spectra was recorded on Bruker Avance 400 NMR Spectrometer operating at 376 MHz (^19^F). High-resolution mass spectroscopy was performed on JEOL SpiralTOF JMS-S3000 MALDI Imaging-TOF/TOF Mass Spectrometer.

### General procedure for the synthesis of dicationic styryl dyes

The starting materials for the synthesis of the dicationic styryl dyes were prepared as follows. First, the nitrogen atom in a heterocyclic ring bearing a methyl group, including 4-picoline, 4-methylquinoline, and 2-methylbenzothiazole (5 mmol) was *N*-methylated by heating with iodomethane (10 mmol) under the neat condition at 90 °C. The aromatic aldehydes with a positively charged side-chain were synthesized from 4-diethylaminosalicylaldehyde or 4-methoxysalicylaldehyde (2.5 mmol) and the α,ω-dibromoalkane (12.5 mmol) in anhydrous DMF (2 mL) in the presence of K_2_CO_3_ (3 mmol) at room temperature with overnight stirring. The DMF was removed from the reaction mixture by water-dichloromethane extraction. The bromoalkylated salicylaldehyde product was purified by silica gel column chromatography [hexanes:EtOAc (4:1); R_f_ = 0.3] and further treated with excess trimethylamine (2mL) in THF (2 mL) at room temperature followed by overnight stirring. Solvent evaporation followed by the addition of diethyl ether provided the charge-modified aromatic aldehyde as a white precipitate.

### 2-(5-(Diethylamino)-2-formylphenoxy)-*N,N,N*-trimethylethan-1-aminium bromide

Yield 67%, ^1^H NMR (500 MHz, DMSO-*d*_*6*_) δ (ppm): 10.00 (s, 1H), 7.53 (d, *J* = 8.9 Hz, 1H), 6.40 (d, *J* = 8.9 Hz, 1H), 6.20 (s, 1H), 4.58 (s, 2H), 3.89–3.82 (m, 2H), 3.47 (q, *J* = 7.0 Hz, 4H), 3.21 (s, 9H), 1.14 (t, *J* = 7.0 Hz, 6H).

### 3-(5-(Diethylamino)-2-formylphenoxy)-*N,N,N*-trimethylpropan-1-aminium bromide

Yield 97%, ^1^H NMR (500 MHz, DMSO-*d*_*6*_) δ (ppm): 10.07 (s, 1H), 7.52 (d, *J* = 8.9 Hz, 1H), 6.37 (d, *J* = 9.0 Hz, 1H), 6.14 (d, *J* = 2.0 Hz, 1H), 4.18 (t, *J* = 5.7 Hz, 2H), 3.59–3.51 (m, 2H), 3.45 (q, *J* = 7.0 Hz, 4H), 3.13 (s, 9H), 2.23 (td, *J* = 11.4, 5.6 Hz, 2H), 1.13 (t, *J* = 7.0 Hz, 6H).

### 4-(5-(Diethylamino)-2-formylphenoxy)-*N,N,N*-trimethylbutan-1-aminium bromide

Yield 91%, ^1^H NMR (500 MHz, DMSO-*d*_*6*_) δ (ppm):10.03 (s, 1H), 7.51 (d, *J* = 8.9 Hz, 1H), 6.35 (d, *J* = 8.9 Hz, 1H), 6.14 (s, 1H), 4.14 (t, *J* = 5.8 Hz, 2H), 3.44 (dd, *J* = 14.2, 7.1 Hz, 6H), 3.07 (s, 9H), 1.95–1.84 (m, 2H), 1.83–1.74 (m, 2H), 1.12 (t, *J* = 7.0 Hz, 6H).

### 4-(2-Formyl-5-methoxyphenoxy)-*N,N,N*-trimethylbutan-1-aminium bromide

Yield 100%, ^1^H NMR (500 MHz, DMSO-*d*_*6*_) δ (ppm): 10.23 (s, 1H), 7.68 (d, *J* = 8.4 Hz, 1H), 6.69 (s, 2H), 4.17 (s, 2H), 3.86 (s, 3H), 3.45–3.36 (m, 2H), 3.06 (s, 9H), 1.96–1.85 (m, 2H), 1.81 (dd, *J* = 11.7, 5.6 Hz, 2H).

Next, the aldol-type condensation was performed by heating the *N*-methylated heterocycles (0.5 mmol) and the charge-modified aromatic aldehyde (0.5 mmol) in refluxing ethanol (2 mL) without any added catalyst for 8 hours for monocationic dyes **PY+** and **BT+**, and 12 hours for dicationic dyes **PY2+(C2)**, **PY2+(C3)**, **PY2+(C4)**, **BT2+(NEt**_**2**_**)**, **BT2+(OMe)** and **4QL2+**. For **4QL+**, the reaction was inefficient and heating in acetic anhydride (2 mL) at 130 °C to allow the acid catalyzed pathway was necessary. The products were isolated by simple filtration and the counterion was replaced with PF_6_^−^ by treatment of the initially obtained bromide or iodide salts with ammonium hexafluorophosphate (1 mmol) in ethanol (2 mL). The products precipitated from the solution as crystalline solids in the range of 11–78%yield (overall yield). The dyes **PY+**^72^, **BT+**^33^, **4QL+** (with the −NMe_2_ substituent instead of −NEt_2_)^10^, and **BT2+(NEt**_**2**_**)**^67^ have been previously reported in the literature, but the rest are new compounds.

### (*E*)-4-(4-(Diethylamino)styryl)-1-methylpyridin-1-ium hexafluorophosphate (PY+)

Red solid, Yield 42%; m.p. = 207–209 °C; ^1^H NMR (500 MHz, DMSO-*d*_6_) δ (ppm): 8.64 (d, *J* = 6.9 Hz, 2H), 8.01 (d, *J* = 6.9 Hz, 2H), 7.87 (d, *J* = 16.1 Hz, 1H), 7.56 (d, *J* = 9.0 Hz, 1H), 7.10 (d, *J* = 16.1 Hz, 1H), 6.74 (d, *J* = 9.0 Hz, 1H), 4.15 (s, 3H), 3.40–3.44 (m, 4H), 1.12 (t, *J* = 7.0 Hz, 6H); ^13^C NMR (126 MHz, DMSO-*d*_6_) δ 153.5, 149.5, 144.3, 142.0, 130.6, 122.0, 121.7, 116.5, 111.4, 46.3, 43.9, 12.6; ^19^F NMR (376 MHz, DMSO-*d*_6_) δ -70.1 (d, ^1^*J*_FP_ = 711.4 Hz); HRMS (MALDI-TOF): (+ve) *m*/*z* calcd for C_18_H_23_N_2_^+^: 267.1856 [*M*]^+^ found: 267.1851; (-ve) *m*/*z* calcd for PF_6_^−^: 144.9642 [*M*]^−^ found: 144.9642.

### (*E*)-4-(4-(Diethylamino)-2-(2-(trimethylammonio)ethoxy)styryl)-1-methylpyridin-1-ium hexafluorophosphate (PY2+(C2))

Red solid, Yield 12%; m.p. = 236–239 °C; ^1^H NMR (500 MHz, DMSO-*d*_6_) δ (ppm): 8.61 (d, *J* = 4.9 Hz, 2H), 7.96 (m, 3H), 7.62 (d, *J* = 8.5 Hz, 1H), 7.12 (d, *J* = 16.0 Hz, 1H), 6.44 (d, *J* = 8.1 Hz, 1H), 6.25 (s, 1H), 4.58 (t, 2H), 4.16 (s, 3H), 3.93 (t, 2H), 3.45 (m, 4H), 3.22 (s, 9H), 1.15 (t, 6H).; ^13^C NMR (126 MHz, DMSO-*d*_6_) δ 158.5, 153.7, 151.2, 144.2, 136.1, 130.2, 121.8, 116.5, 110.8, 105.4, 94.6, 64.4, 61.8, 53.2, 46.3, 44.1, 12.7; ^19^F NMR (376 MHz, DMSO-*d*_6_) δ -70.1 (d, ^1^*J*_FP_ = 711.4 Hz); HRMS (MALDI-TOF): (+ve) *m*/*z* calcd for C_23_H_34_N_3_O^+^: 368.2701 [*M-H*]^+^ found: 368.2725; (-ve) *m*/*z* calcd for PF_6_^−^: 144.9642 [*M*]^−^ found: 144.9642.

### (*E*)-4-(4-(Diethylamino)-2-(3-(trimethylammonio)propoxy)styryl)-1-methylpyridin-1-ium hexafluorophosphate (PY2+(C3))

Red solid, Yield 11%; m.p. = 238–240 °C; ^1^H NMR (500 MHz, DMSO-*d*_6_) δ (ppm): 8.60 (d, *J* = 6.9 Hz, 2H), 7.96 (m, 3H), 7.58 (d, *J* = 9.0 Hz, 1H), 7.13 (d, *J* = 16.1 Hz, 1H), 6.42 (dd, *J* = 9.0, 2.2 Hz, 1H), 6.21 (s, 1H), 4.20–4.12 (m, 5H), 3.58–3.52 (m, 2H), 3.46 (q, 4H), 3.14 (s, 9H), 2.32 (dd, *J* = 10.5, 5.7 Hz, 2H), 1.14 (t, *J* = 7.0 Hz, 6H); ^13^C NMR (126 MHz, DMSO-*d*_6_); δ 159.4, 153.8, 151.2, 144.1, 136.9, 130.8, 121.8, 116.6, 111.1, 105.2, 94.6, 65.2, 63.1, 52.4, 46.2, 44.1, 22.6, 12.6; ^19^F NMR (376 MHz, DMSO-*d*_6_) δ -70.1 (d, ^1^*J*_FP_ = 711.4 Hz); HRMS (MALDI-TOF): (+ve) *m*/*z* calcd for C_24_H_36_N_3_O^+^: 382.2858 [*M-H*]^+^ found: 382.2897; (-ve) *m*/*z* calcd for PF_6_^−^: 144.9642 [*M*]^−^ found: 144.9642.

### (*E*)-4-(4-(Diethylamino)-2-(4-(trimethylammonio)butoxy)styryl)-1-methylpyridin-1-ium hexafluorophosphate (PY2+(C4))

Red solid, Yield 76%; m.p. = 244–246 °C; ^1^H NMR (500 MHz, DMSO-*d*_6_) δ (ppm): 8.60 (d, *J* = 5.9 Hz, 2H), 7.97 (m, 3H), 7.57 (d, *J* = 8.0 Hz, 1H), 7.16 (d, *J* = 16.0 Hz, 1H), 6.40 (d, *J* = 8.9 Hz, 1H), 6.21 (s, 1H), 4.16 (m, 5H), 3.54–3.43 (m, 6H), 3.09 (s, 9H), 1.89 (d, *J* = 6.9 Hz, 4H), 1.13 (t, *J* = 6.6 Hz, 3H); ^13^C NMR (126 MHz, DMSO-d_6_) δ 159.7, 153.8, 151.2, 144.1, 137.1, 131.2, 121.7, 116.5, 111.2, 105.0, 94.5, 67.1, 65.1, 52.2, 46.2, 44.1, 25.6, 19.5, 12.6; ^19^F NMR (376 MHz, DMSO-*d*_6_) δ -70.1 (d, ^1^*J*_FP_ = 711.4 Hz); HRMS (MALDI-TOF): (+ve) *m*/*z* calcd for C_25_H_38_N_3_O^+^: 396.3015 [*M-H*]^+^ found: 396.3040; (−ve) *m*/*z* calcd for PF_6_^−^: 144.9642 [*M*]^−^ found: 144.9642.

### (*E*)-2-(4-(Diethylamino)styryl)-3-methylbenzo[d]thiazol-3-ium hexafluorophosphate (BT+)

Purple solid, Yield 78%; m.p. = 204–206 °C; ^1^H NMR (500 MHz, DMSO-*d*_6_) δ (ppm): 8.27 (d, *J* = 8.9 Hz, 1H), 8.05 (d, *J* = 8.4 Hz, 1H), 8.01 (d, *J* = 15.2 Hz, 1H), 7.86 (d, *J* = 8.9 Hz, 2H), 7.76 (t, 1H), 7.67 (t, 1H), 7.55 (d, *J* = 15.2 Hz, 1H), 6.80 (d, *J* = 9.1 Hz, 2H), 4.19 (s, 3H), 3.53–3.46 (m, 4H), 1.15 (t, *J* = 7.1 Hz, 3H); ^13^C NMR (126 MHz, DMSO-*d*_6_) δ 171.2, 151.5, 150.1, 142.0, 133.3, 128.9, 127.4, 126.7, 123.8, 121.1, 115.9, 111.6, 105.6, 44.3, 35.5, 12.6; ^19^F NMR (376 MHz, DMSO-*d*_6_) δ -70.1 (d, *J* = 711.4 Hz); HRMS (MALDI-TOF): (+ve) *m*/*z* calcd for C_20_H_23_N_2_S^+^: 323.1576 [*M*]^+^ found: 323.1575; (−ve) *m*/*z* calcd for PF_6_^−^: 144.9642 [*M*]^−^ found: 144.9642.

### (*E*)-2-(4-(Diethylamino)-2-(4-(trimethylammonio)butoxy)styryl)-3-methylbenzo[d]thiazol-3-ium hexafluorophosphate (BT2+(NEt_2_))

Purple Solid, Yield 45%; m.p. = 238–240 °C; ^1^H NMR (500 MHz, DMSO-*d*_6_) δ (ppm): 8.18 (d, *J* = 7.9 Hz, 1H), 8.12 (d, *J* = 15.1 Hz, 1H), 8.03 (d, *J* = 8.3 Hz, 1H), 7.90 (d, *J* = 9.1 Hz, 1H), 7.75 (t, *J* = 7.7 Hz, 1H), 7.64 (t, *J* = 7.6 Hz, 1H), 7.50 (d, *J* = 15.2 Hz, 1H), 6.54 (d, *J* = 8.9 Hz, 1H), 6.21 (s, 1H), 4.21 (t, 2H), 4.12 (s, 3H), 3.64–3.47 (m, 6H), 3.09 (s, 9H), 1.92 (q, 4H), 1.18 (t, *J* = 6.9 Hz, 6H); ^13^C NMR (126 MHz, DMSO-*d*_6_) δ 171.1, 161.3, 153.8, 146.3, 144.2, 142.0, 128.8, 127.2, 126.2, 123.6, 115.6, 111.0, 106.3, 104.4, 93.9, 67.3, 65.1, 52.3, 44.5, 35.1, 25.6, 19.2, 12.7; ^19^F NMR (376 MHz, DMSO-*d*_6_) δ -70.1 (d, ^1^*J*_FP_ = 711.3 Hz); HRMS (MALDI-TOF): (+ve) *m*/*z* calcd for C_27_H_38_N_3_OS^+^: 452.2735 [*M-H*]^+^ found: 452.2774; (−ve) *m*/*z* calcd for PF_6_^−^: 144.9642 [*M*]^−^ found: 144.9642; .

### (*E*)-2-(4-Methoxy-2-(4-(trimethylammonio)butoxy)styryl)-3-methylbenzo[d]thiazol-3-ium hexafluorophosphate (BT2+(OMe))

Orange solid, Yield 43%; m.p. 242–245 °C; ^1^H NMR (500 MHz, DMSO-*d*_6_) δ (ppm): 8.33 (d, *J* = 8.0 Hz, 1H), 8.21 (m, 2H), 8.10 (d, *J* = 8.8 Hz, 1H), 7.86 (m, 2H), 7.77 (t, *J* = 7.7 Hz, 1H), 6.78 (dd, *J* = 8.8, 2.2 Hz, 1H), 6.73 (d, *J* = 2.2 Hz, 1H), 4.27 (s, 3H), 4.23 (t, *J* = 5.4 Hz, 2H), 3.90 (s, 3H), 3.42–3.39 (m, 2H), 3.09 (s, 9H), 1.92 (q, 4H); ^13^C NMR (126 MHz, DMSO-*d*_6_) δ 172.4, 165.2, 160.1, 143.3, 142.1, 132.2, 129.4, 128.2, 127.3, 124.0, 116.7, 115.6, 110.7, 107.6, 99.2, 67.8, 65.0, 56.0, 52.3, 36.0, 25.5, 19.1; ^19^F NMR (376 MHz, DMSO-*d*_6_) δ -70.1 (d, ^1^*J*_FP_ = 711.4 Hz); HRMS (MALDI-TOF): (+ve) *m*/*z* calcd for C_24_H_31_N_2_O_2_S^+^: 411.2106 [*M-H*]^+^ found: 411.2129; (−ve) *m*/*z* calcd for PF_6_^−^: 144.9642 [*M*]^−^ found: 144.9642.

### (*E*)-4-(4-(Diethylamino)styryl)-1-methylquinolin-1-ium hexafluorophosphate (4QL+)

Purple solid, Yield 52%; m.p. = 205–208 °C; ^1^H NMR (500 MHz, DMSO-*d*_6_) δ (ppm): 9.05 (d, *J* = 6.5 Hz, 1H), 8.99 (d, *J* = 8.5 Hz, 1H), 8.30 (m, 2H), 8.23–8.09 (m, 2H), 7.94 (m, 2H), 7.83 (d, *J* = 8.5 Hz, 2H), 6.78 (d, *J* = 8.6 Hz, 2H), 4.41 (s, 3H), 3.44 (m, 4H), 1.15 (t, *J* = 6.8 Hz, 6H); ^13^C NMR (126 MHz, DMSO-*d*_6_) δ 153.2, 150.1, 146.7, 144.8, 138.8, 134.6, 131.9, 128.6, 126.3, 125.7, 122.5, 119.1, 113.7, 112.5, 111.5, 44.1, 44.0, 12.6; ^19^F NMR (376 MHz, DMSO-*d*_6_) δ -70.1 (d, ^1^*J*_FP_ = 712.1 Hz); HRMS (MALDI-TOF): (+ve) *m*/*z* calcd for C_22_H_25_N_2_^+^: 317.2012 [*M*]^+^ found: 317.2015; (−ve) *m*/*z* calcd for PF_6_^−^: 144.9642 [*M*]^−^ found: 144.9642.

### (*E*)-4-(4-(Diethylamino)-2-(4-(trimethylammonio)butoxy)styryl)-1-methylquinolin-1-ium hexafluorophosphate (4QL2+)

Purple solid, Yield 19%; m.p. = 248–250 °C; ^1^H NMR (500 MHz, DMSO-*d*_6_) δ (ppm): 8.93 (d, *J* = 6.5 Hz, 1H), 8.84 (d, *J* = 8.5 Hz, 1H), 8.28 (m, 2H), 8.17 (m, 2H), 8.01–7.91 (m, 3H), 6.47 (d, *J* = 9.0 Hz, 1H), 6.24 (s, 1H), 4.40 (s, 3H), 4.21 (t, 2H), 3.54–3.47 (m, 6H), 3.07 (s, 9H), 1.93 (q, 4H), 1.17 (t, *J* = 6.7 Hz, 3H); ^13^C NMR (126 MHz, DMSO-*d*_6_) δ 160.2, 153.4, 152.0, 146.3, 139.3, 138.9, 134.5, 131.7, 128.5, 125.9, 125.6, 119.1, 113.1, 112.1, 112.0, 105.4, 94.3, 67.2, 65.2, 52.3, 44.2, 43.9, 25.8, 19.4, 12.7; ^19^F NMR (376 MHz, DMSO-*d*_6_) δ -70.1 (d, ^1^*J*_FP_ = 711.4 Hz); HRMS (MALDI-TOF): (+ve) *m*/*z* calcd for C_29_H_40_N_3_O^+^: 446.3171 [*M-H*]^+^ found: 446.3202; (−ve) *m*/*z* calcd for PF_6_^−^: 144.9642 [*M*]^−^ found: 144.9642.

### Spectroscopic measurements

Samples for absorption and fluorescence studies were prepared in 10 mM sodium phosphate buffer (PB) pH 7.0. The concentration of the DNA was determined by UV spectrophotometry using the molar extinction coefficient at 260 nm (ε_260_) as calculated from the base sequence. Double-stranded DNA used in this work was prepared by annealing two complementary synthetic DNA sequences (30 nt each, see the sequences in Table S2). The stock solution of the styryl dye was prepared by dissolving the specific amount of the dye as crystalline solid in methanol at 1 mM final concentration. To prepare the sample solution for spectroscopic measurements, both dyes and DNA stock solutions were aliquoted and diluted in 10 mM sodium phosphate buffer (pH 7.0) to the specified concentrations with a final volume of 1 mL. The absorption spectra were measured on a CARY 100 Bio UV-vis spectrophotometer (Varian, Australia) and the fluorescence spectra were collected on a Cary Eclipse Fluorescence Spectrophotometer (Varian/Agilent Technologies) using a quartz cuvette with a path length of 1.0 cm at ambient temperature (25 °C). The size of excitation and emission slits for the spectrofluorimetric measurements are 5 nm.

### Fluorescence quantum yield (*Φ*_F_) determination^73^

The fluorescence quantum yields (*Φ*_F_) of the dyes, both in the free form or bound with dsDNA were calculated using fluorescein (*Φ*_F_ = 0.95, λ_ex_ = 470–490 nm), rhodamine 6G (*Φ*_F_ = 0.95, λ_ex_ = 470–510 nm), cresyl violet (*Φ*_F_ = 0.54, λ_ex_ = 540–590 nm) as the standard^73^. The integrated fluorescence intensities and the absorbance values (at λ_ex_) of the standard and the samples were plotted and the slopes were investigated to give grad_standard_ and grad_sample_, respectively.

The quantum yield can be calculated according to Eq. (1):

1 $$\varPhi_{{{\text{sample}}}} = \varPhi_{{{\text{standard}}}} \left( {{\text{grad}}_{{{\text{sample}}}} /{\text{grad}}_{{{\text{standard}}}} } \right) \, \left( {\upeta _{{{\text{sample}}}}^{2} /\upeta _{{{\text{standard}}}}^{2} } \right)$$

where grad is the slope from the plot of integrated fluorescence intensity as a function of absorbance and η is the refractive index of the solvent used for the fluorescence measurement.

### Study of the solvatochromic properties of the cationic styryl dyes

To investigate the mechanism of the optical change of the cationic styryl dyes in the presence of DNA, **4QL2+** was dissolved in water, acetronitrile, acetone, ethyl acetate, DMSO, methanol, glycerol, ethanol, and dichloromethane to the same concentration at 10 µM with the final volume of 1 mL in all cases. Solvatochromic behavior of the dye in various solvents was studied using Lippert–Mataga Eq. (2)^49^.

2 $$\Delta \overline{\upsilon } = \overline{\upsilon }_{A} {-}\overline{\upsilon }_{f} = \frac{2}{hc} \left( {\frac{{\varepsilon {-} 1}}{2\varepsilon + 1}{-} \frac{{n^{2} {-} 1}}{{2n^{2} + 1}}} \right) \frac{{\left( {\mu_{{\text{E}}} {-}\mu_{{\text{G}}} } \right)^{2} }}{{a^{3} }} + k$$

where *h* = Planck constant, *c* = light speed in vacuum, *a* = radius of the cavity where the dyes is allocated, $\overline{\upsilon }_{{\text{A }}}$ = absorption wavenumber, $\overline{\upsilon }_{{\text{f }}}$ = emission wavenumber, *k* = difference between the absorption and emission wavenumbers in the vacuum.

3 $$\Delta f = \frac{{\varepsilon {-} 1}}{2\varepsilon + 1}{-} \frac{{n^{2} {-} 1}}{{2n^{2} + 1}}$$

where ${\Delta }f$ = orientation polarizability which is the combination of dielectric constant ($\varepsilon$) and refractive index (*n*) parameters of solvents.

### Fluorescent indication displacement assay

In this study, two DNA sequences were employed including d(AT)_10_ and d(GC)_10_ (Table S2) were employed as models for groove binding and intercalation studies, respectively. Ethidium bromide (EtBr) and 4′,6-diamidino-2-phenylindole (DAPI) were used as reference intercalating and groove binding dyes, respectively. The optimal ratios between indicator dyes and dsDNAs prior to the displacement assay, and the values of 2:5 and 1:10 for EtBr:dsDNA(bp) and DAPI:dsDNA(bp) were obtained, respectively. For the dye displacement assay, the reference dyes were mixed and incubated with the dsDNA for 30 minutes at room temperature (25 °C). Next, various amounts of the cationic styryl dyes (0.5–50 µM) were added to the prepared mixtures, at a fixed concentration of dsDNA (bp) of 20 µM. After 15 minutes of incubation, the fluorescence spectra were recorded by Cary Eclipse Fluorescence Spectrophotometer (Varian/Agilent Technologies). Excitation wavelengths used in this study were 341 nm for DAPI, 545 nm for EtBr, and the optimal excitation wavelength of each styryl dyes (450 nm for **BT2+(OMe)**, 480 nm for **PY+** and **PY2+(C4)**, 565 nm for **BT+** and **BT2+(NEt**_**2**_**)**, 548 nm for **4QL+** and **4QL2+**). All spectra were recorded starting with a 10 nm offset from the excitation wavelength to 800 nm.

### Ionic strength dependence studies

The samples containing dsDNA (15 µM bp) and representative dyes, including **BT+**, **BT2+(NEt**_**2**_**)**, **PY2+(C4)** and **4QL2+** (1 µM) in 10 mM sodium phosphate buffer (pH 7.0) were prepared in a 96-well microplate. Next, various concentrations of NaCl (50–200 mM) were added with the final volume adjusted to 200 µL in all cases, followed by fluorescent measurement on a PerkinElmer, EnSight Multimode Microplate Reader. Optimal excitation wavelength of each styryl dyes, referred to the previous session was used. The measured fluorescence intensities at the wavelengths of interest (600 nm for **PY2+(C4)**, **BT+** and **BT2+(NEt**_**2**_**)**, 670 nm for **4QL2+**) were used to calculate the Stern-Volmer quenching constant according to Eq. (4)^58^.

4 $$F_{0} /F = {1} + K_{{{\text{SV}}}} \left[ {\text{Q}} \right]$$

where *F*_0_ and *F* are the fluorescence intensities in the absence and presence of NaCl (50–200 mM), as the quencher [Q] and *K*_SV_ is the Stern-Volmer quenching constant.

### Dyes-DNA binding interaction studies

The sequence of double-stranded DNA (30 bp) used in this study is shown in Table S2. The selected dyes consisted of **PY+**, **PY2+(C4)**, **BT+**, **BT2+(NEt**_**2**_**)**, **4QL+**, and **4QL2+**. The dye-DNA mixtures at various dye-DNA(in bp) ratios were prepared in 10 mM PB (pH 7.0) at a fixed concentration of dyes (2 µM) in 96-well microplate with a final volume of 200 µL. Next, fluorescent intensities at the desired wavelengths were recorded on a PerkinElmer, EnSight Multimode Microplate Reader. The intensities were recorded until they reached constant values which represented excess amounts of DNA in the system. Only the data at optimal ratios for dye-DNA(in bp) which showed linear response were further collected for calculation. The binding constant (*K*_b_) and binding site (*n*) values were calculated from fluorescent titration curves using the modified McGhee and von Hippel equation according to Akbay and co-workers^52^ (Eq. 5):

5 $$Y = F_{\max } {-} \frac{X}{{C_{{{\text{dye}}}} K_{{\text{b}}} }}\frac{{\left( {1{-}\left[ {(n{-}1)\frac{X}{{F_{\max } }}} \right]} \right)^{{n {-} 1}} }}{{\left( {1{-}n\frac{X}{{F_{\max } }}} \right)^{n} }}$$

where Y = fluorescence intensity at maximum emission wavelength (F) and X = F×C_dye_/C_DNA_ (bp). Therefore, *K*_b_, *n* and *F*_max_ values can be calculated as approximation parameters of fitting by the Eq. 5 the experimentally obtained data plotted as the dependence of Y on X.

### Docking simulations of DNA-dye interaction

To calculate free energy of DNA-dye binding (ΔG), **BT+**, **BT2+(NEt**_**2**_**)**, **PY2+(C4)**, and **4QL2+** were selected as model ligands. Firstly, SMILES string of each ligand was generated by PerkinElmer ChemDraw^®^, followed by 3D model building, minimization, torsion adjustment and atomic partial charges calculation by Discovery Studio 2018. DNA structures were obtained from RCSB with removal of water, ligands, and salts molecules by ChimeraX, and addition of polar H atoms and Gasteiger charges by AutodockTools. Drew-Dickerson (PDB code: 4C64) DNA crystal structure was used as a model for a minor groove interaction, while the most-averaged NMR structure of a dsDNA-intercalator complex (PDB code: 108D) was applied as an intercalation model. Docking simulations of DNA-dye interaction were performed by AutoDock 4.2.6^61^. Grid boxes were generated with 60 × 75 × 120 Å and 60 × 110 × 60 Å dimensions to cover the entire 4C64 and 108D DNA models, respectively. Lamarckian Genetic Algorithm (LGA) was chosen as a docking algorithm in which each docking consisted of 20 independent runs, with a maximum number of 5 × 10^6^ energy evaluations, and a maximum number of 27000 generations. The best docking structures were visualized by ChimeraX software.

### *E. coli* and *S. aureus* assays by LAMP-4QL2+

The designed LAMP primer sets for the amplification of *E. coli* and *S. aureus* consisted of FIP, BIP, F3, B3, LF, and LB (Table S3). The LAMP reaction (15 µL) comprised 0.2 µM each of primers F3 and B3, 0.8 µM primers FIP and BIP, 0.4 µM primers LF and LB. The LAMP were performed at 1.4 mM dNTP, 0.3 M betaine, 6 mM MgSO_4_, 8 U *Bst* DNA polymerase and 1 µL of template DNA. The detection conditions (shortest incubation time with highest sensitivity and correct specificity) were optimized using incubation temperature of 61, 63 and 65 °C, and incubation periods of 30, 45 and 60 min. The LAMP products appeared as intercalating bands of various bp sizes by 1.75% agarose gel electrophoresis. Naked-eyes detection with **4QL2+** dye was also used to find the optimal condition for dye color changing by mixing 2 µL LAMP product, 2 µM **4QL2+**, and sodium phosphate buffer (pH 7.0) with 10 µL total volume.

In the specificity assays, various strains of *E. coli* and *S. aureus*, as positive controls and other common food poisoning bacteria were used as negative controls (Table S4), were tested for specificity. For the sensitivity assays, 10-fold serial dilutions of *E. coli* ATCC 25922 (3.39 × 10^3^–0.339 copies of DNA), and *S. aureus* ATCC 25923 (3.3 × 10^2^–0.033 copies of DNA) were used as a template to determine the minimum amounts of template DNA that can be detected by LAMP-**4QL2+** assay. Gel electrophoresis was performed in 1.75% agarose gel at 100 V for 35 min.

### Cellular imaging experiments

HeLa cells (a gift from the Lampson Lab, Department of Biology, the University of Pennsylvania, PA, USA) were cultured in Dulbecco’s Modified Eagle's Medium (DMEM, HyClone™ Thermo Scientific, USA) containing 4.5 g/L D-glucose, 4 mM L-glutamine and supplemented with 10% of heat-inactivated fetal bovine serum (FBS; GibcoTM Invitrogen) and 1% penicillin at 37 °C at 5% CO_2_. The HeLa cells were seeded on a 24-well chamber cell culture plate at 5 × 10^4^ cells per well. The cells were grown in an incubator at 37 °C and 5% CO_2_. For fixed-cell staining, the Hela cells were fixed with paraformaldehyde (4% in PBS) for 10 min and then stained with 20 µM **BT2+(NEt**_**2**_**)** at 37 °C for 45 min and 500 nM SYTO RNA select^®^ Green for 20 min (as individual dyes or co-stained) at 37 °C and 5% CO_2_. After incubation time, the cells were stained with DAPI (0.2 µg/mL) and further incubated for 15 minutes. The fluorescence images of the fixed cells were recorded using fluorescence microscope (ZEISS Axio Observer).

### MTT cytotoxicity assays

HeLa cells (a gift from the Lampson Lab, Department of Biology, the University of Pennsylvania, PA, USA) were seeded in 96-well plates at 1 × 10^4^ cells per well. The cells were incubated at 37 °C in a humidification incubator with 5% CO_2_. After incubation, the cells were treated with different concentrations of 0.1, 1, 10, 25 µM dyes (**BT+** and **BT2+(NEt**_**2**_**)**). They were incubated at the same condition for 24 hours. MTT solution (5 mg/mL of 3-(4,5-dimethylthiazol-2-yl)-2,5-diphenyltetrazolium bromide in PBS) was added to the wells and again incubated at 37 °C for 3 hours. The media was then removed and mixed with DMSO. The absorbance at 570 nm was measured with a PerkinElmer, EnSight Multimode Microplate Reader and the cytotoxicity was calculated.

## Supplementary Information

Supplementary Information.

## Data Availability

The pathogens DNA sequences involved in the study were obtained from the literature and the relevant references were given in the supplementary material. Validated DNA-ligand structures (4C64 and 108D) from RCSB PDB (https://www.rcsb.org)^74^ were used as a primary data source for the docking studies. The docking coordinate files can be obtained from the corresponding author upon request.
