# Supplementary material for: Dicationic styryl dyes for colorimetric and fluorescent detection of nucleic acids
Source: Sci Rep. 2022 Aug 22;12:14250. doi: 10.1038/s41598-022-18460-w (PMC9395382; doi:10.1038/s41598-022-18460-w)
Supplement: Supplementary file 1 — Supplementary Information. [file 41598_2022_18460_MOESM1_ESM.pdf]

Dicationic Styryl Dyes for Colorimetric and Fluorescent Detection of Nucleic Acids

Kotchakorn Supabowornsathit,[1] Kriangsak Faikhruea,[1] Boonsong Ditmangklo,[1] Theeranuch Jaroenchuensiri,[2] Sutthida Wongsuwan,[2] Sirikarn Junpra-ob,[3] Ilada Choopara,[3] Tanapat Palaga,[3] Chanat Aonbangkhen,[2] Naraporn Somboonna,[3,4] Jaru Taechalertpaisarn,[5] and Tirayut Vilaivan[1,*]

[1]Organic Synthesis Research Unit, Department of Chemistry, Faculty of Science, Chulalongkorn University, Phayathai Road, Pathumwan, Bangkok 10330, Thailand
[2]Center of Excellence in Natural Products Chemistry (CENP), Department of Chemistry, Faculty of Science, Chulalongkorn University, Phayathai Road, Pathumwan, Bangkok 10330, Thailand
[3]Department of Microbiology, Faculty of Science, Chulalongkorn University, Phayathai Road, Pathumwan, Bangkok 10330, Thailand
[4]Microbiome Research Unit for Probiotics in Food and Cosmetics, Chulalongkorn University, Bangkok 10330, Thailand
[5]Department of Chemistry and Biochemistry, University of California, Santa Cruz, California 95064, United States
*E-mail: vtirayut@chula.ac.th

Table of Contents

Item	Description	Page
**Figure S1**	Synthesis of the cationic styryl dyes	5
**Figure S2**	Normalized absorption spectra in various solvents, relationship between absorption wavenumber and $E_T(30)$ scale, and absorption spectra in aqueous solution of **4QL2+**	5
**Figure S3**	Fluorescence and absorption spectra of **4QL2+** in the presence and absence of DNA in water and glycerol, fluorescence spectra of **4QL2+** in glycerol (0 - 90 °C), and illustration explained fluorescence change mechanism of the dye	6
**Figure S4**	Fluorescence spectra, normalized fluorescence spectra, relative fluorescence spectra of **4QL2+** in various solvents, and Lippert-Mataga plot of the Stokes shift and orientation polarizability of the solvents surrounding the dye molecules of **4QL2+** dissolved in various solvents	6
**Figure S5**	Illustration explained energy gaps of **4QL2+** in different polarity environment	7
**Figure S6**	Absorption and fluorescence spectra displaying selectivity of **4QL2+** towards target dsDNA, comparing with PSS and BSA at same concentrations	7
**Figure S7**	UV-vis and fluorescence titration of **PY+** (10 µM) with DNA (3 – 450 µM, in bp) in 10 mM sodium phosphate buffer pH 7.0	8
**Figure S8**	UV-vis and fluorescence titration of **PY2+(C2)** (10 µM) with DNA (3 – 450 µM, in bp) in 10 mM sodium phosphate buffer pH 7.0	8
**Figure S9**	UV-vis and fluorescence titration of **PY2+(C3)** (10 µM) with DNA (3 – 450 µM, in bp) in 10 mM sodium phosphate buffer pH 7.0	8
**Figure S10**	UV-vis and fluorescence titration of **PY2+(C4)** (10 µM) with DNA (3 – 450 µM, in bp) in 10 mM sodium phosphate buffer pH 7.0	9
**Figure S11**	UV-vis and fluorescence titration of **BT+** (10, 1 µM) with DNA (3 – 450 µM, 0.3 –45 µM in bp) in 10 mM sodium phosphate buffer pH 7.0	9
**Figure S12**	UV-vis and fluorescence titration of **BT2+(NEt$_2$)** (10, 1 µM) with DNA (3 – 450 µM, 0.3 –45 µM, in bp) in 10 mM sodium phosphate buffer pH 7.0	9
**Figure S13**	UV-vis and fluorescence titration of **BT2+(OMe)** (10 µM) with DNA (3 – 450 µM, in bp) in 10 mM sodium phosphate buffer pH 7.0	10
**Figure S14**	UV-vis and fluorescence titration of **4QL+** (10 µM) with DNA (3 – 450 µM, in bp) in 10 mM sodium phosphate buffer pH 7.0	10
**Figure S15**	UV-vis and fluorescence titration of **4QL2+** (10 µM) with DNA (3 – 450 µM, in bp) in 10 mM sodium phosphate buffer pH 7.0	10
**Figure S16**	Plot of relative change of maximum absorption and maximum fluorescence emission vs. concentration ratio of DNA (bp) and mono-/dicationic dyes	11
**Figure S17**	Plot of fluorescence intensity dependence on F×[Dye]/[DNA](bp) ratio and its approximation by the modified Mcghee and von Hippel equation for **PY+**, **PY2+(C4)**, **BT+**, **BT2+(NEt$_2$)**, **4QL+,** and **4QL2+**	12
**Figure S18**	Plot of relative change of maximum absorption and maximum fluorescence emission vs. concentration ratio of DNA (bp) and dyes with different linker lengths.	12

**Figure S19**	Fluorescent indicator displacement assay with (AT)10 and (GC)10 DNAs, based on EtBr as intercalated indicator and DAPI as groove bound indicator of **PY+**	13
**Figure S20**	Fluorescent indicator displacement assay with (AT)10 and (GC)10 DNAs, based on EtBr as intercalated indicator and DAPI as groove bound indicator of **PY2+(C4)**	14
**Figure S21**	Fluorescent indicator displacement assay with (AT)10 and (GC)10 DNAs, based on EtBr as intercalated indicator and DAPI as groove bound indicator of **BT+**	15
**Figure S22**	Fluorescent indicator displacement assay with (AT)10 and (GC)10 DNAs, based on EtBr as intercalated indicator and DAPI as groove bound indicator of **BT2+(NEt$_2$)**	16
**Figure S23**	Fluorescent indicator displacement assay with (AT)10 and (GC)10 DNAs, based on EtBr as intercalated indicator and DAPI as groove bound indicator of **BT2+(OMe)**	17
**Figure S24**	Fluorescent indicator displacement assay with (AT)10 and (GC)10 DNAs, based on EtBr as intercalated indicator and DAPI as groove bound indicator of **4QL+**	18
**Figure S25**	Fluorescent indicator displacement assay with (AT)10 and (GC)10 DNAs, based on EtBr as intercalated indicator and DAPI as groove bound indicator of **4QL2+**	19
**Figure S26**	Docking simulation for DNA-dye minor groove binding interaction with 4c64 model	20
**Figure S27**	Docking simulation for DNA-dye intercalating interaction with 108D model	21
**Figure S28**	Cell viability of HeLa cells treated with **BT+** and **BT2+(NEt$_2$)** dyes	22
**Figure S29**	Fluorescence image analysis of HeLa cells treated with **BT2+(NEt$_2$)** dye, costained with SYTO RNA select® Green and DAPI nuclear staining dye	22
**Figure S30**	^{1}H (500 MHz, DMSO-d_6) NMR spectrum of 2-(5-(diethylamino)-2-formylphenoxy)-*N*,*N*,*N*-trimethylethan-1-aminium bromide	23
**Figure S31**	^{1}H (500 MHz, DMSO-d_6) NMR spectrum of 3-(5-(diethylamino)-2-formylphenoxy)-*N*,*N*,*N*-trimethylpropan-1-aminium bromide	23
**Figure S32**	^{1}H (500 MHz, DMSO-d_6) NMR spectrum of 4-(5-(diethylamino)-2-formylphenoxy)-*N*,*N*,*N*-trimethylbutan-1-aminium bromide	24
**Figure S33**	^{1}H (500 MHz, DMSO-d_6) NMR spectrum of 4-(2-formyl-5-methoxyphenoxy)-*N*,*N*,*N*-trimethylbutan-1-aminium bromide	24
**Figure S34**	^{1}H (500 MHz, DMSO-d_6) and ^{13}C (126 MHz, DMSO-d_6) NMR spectra of compound **PY+**	25
**Figure S35**	^{1}H (500 MHz, DMSO-d_6) and ^{13}C (126 MHz, DMSO-d_6) NMR spectra of compound **PY2+(C2)**	26
**Figure S36**	^{1}H (500 MHz, DMSO-d_6) and ^{13}C (126 MHz, DMSO-d_6) NMR spectra of compound **PY2+(C3)**	27
**Figure S37**	^{1}H (500 MHz, DMSO-d_6) and ^{13}C (126 MHz, DMSO-d_6) NMR spectra of compound **PY2+(C4)**	28
**Figure S38**	^{1}H (500 MHz, DMSO-d_6) and ^{13}C (126 MHz, DMSO-d_6) NMR spectra of compound **BT+**	29
**Figure S39**	^{1}H (500 MHz, DMSO-d_6) and ^{13}C (126 MHz, DMSO-d_6) NMR spectra of compound **BT2+(NEt$_2$)**	30
**Figure S40**	^{1}H (500 MHz, DMSO-d_6) and ^{13}C (126 MHz, DMSO-d_6) NMR spectra of compound **BT2+(OMe)**	31
**Figure S41**	^{1}H (500 MHz, DMSO-d_6) and ^{13}C (126 MHz, DMSO-d_6) NMR spectra of compound **4QL+**	32

**Figure S42**	^{1}H (500 MHz, DMSO-d_6) and ^{13}C (126 MHz, DMSO-d_6) NMR spectra of compound **4QL2+**	33
**Figure S43**	^{19}F (376 MHz, DMSO-d_6) NMR spectra of compounds **PY+**, **PY2+(C2)**, **PY2+(C3)**, **PY2+(C4)**, **BT+**, **BT2+(NEt$_2$)**, **BT2+(OMe)**, **4QL+**, and **4QL2+**	34
**Figure S44**	HRMS (MALDI-TOF) of **PY+**	35
**Figure S45**	HRMS (MALDI-TOF) of **PY2+(C2)**	36
**Figure S46**	HRMS (MALDI-TOF) of **PY2+(C3)**	37
**Figure S47**	HRMS (MALDI-TOF) of **PY2+(C4)**	38
**Figure S48**	HRMS (MALDI-TOF) of **BT+**	39
**Figure S49**	HRMS (MALDI-TOF) of **BT2+(NEt$_2$)**	40
**Figure S50**	HRMS (MALDI-TOF) of **BT2+(OMe)**	41
**Figure S51**	HRMS (MALDI-TOF) of **4QL+**	42
**Figure S52**	HRMS (MALDI-TOF) of **4QL2+**	43
**Table S1**	Optical properties of **4QL2+** in various solvents: absorption maxima λ_{max}(abs), emission maxima λ_{max}(em), and stroke shifts	44
**Table S2**	DNA List in this study	44
**Table S3**	Primer sequences for LAMP-4QL2+ assay	45
**Table S4**	Positive and negative control bacterial strains for specificity test	45
**References**		46

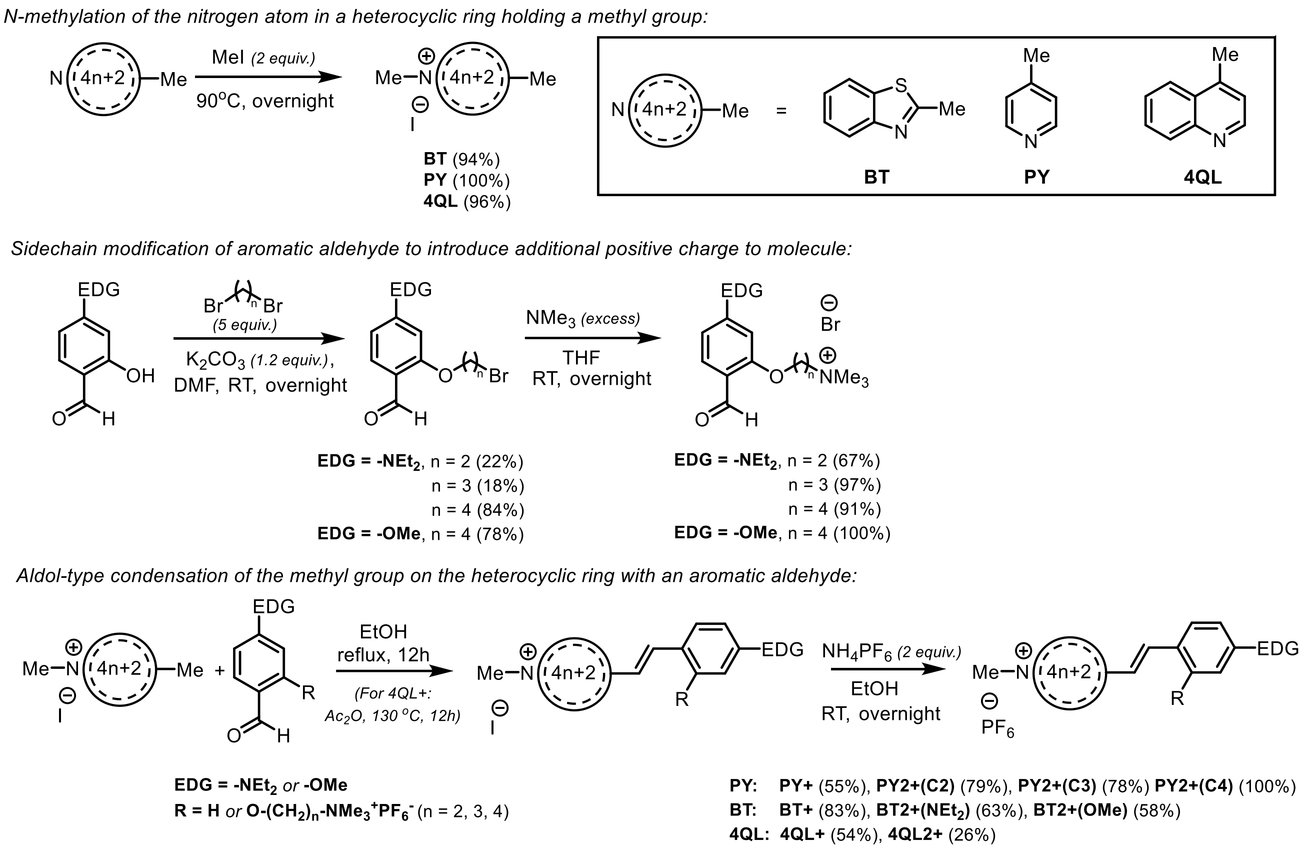

Figure S1. Synthesis of the cationic styryl dyes

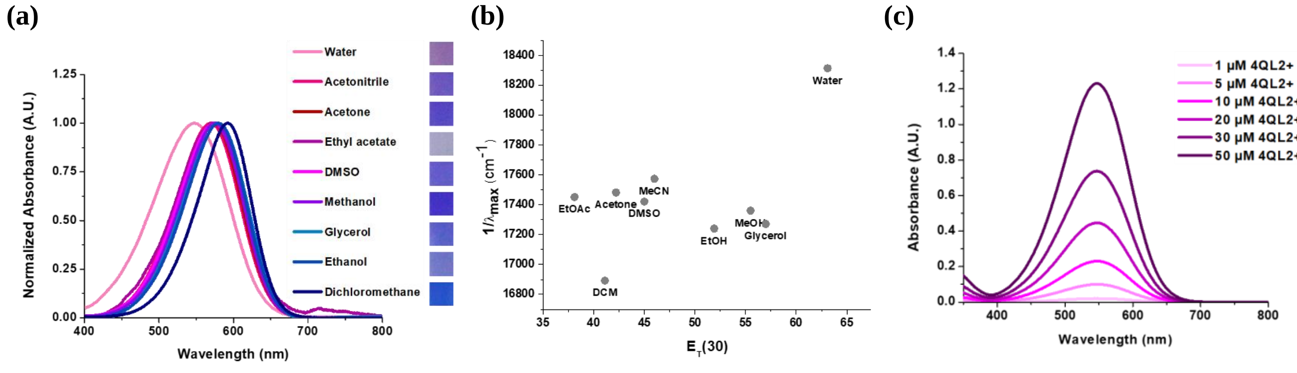

Figure S2. (a) Normalized absorption spectra of **4QL2+** (10 μM) in various solvents (b) Relationship between absorption wavenumber ($1/\lambda_{max}$(abs), cm^{-1}) and $E_T(30)$ scale (c) Absorption spectra of **4QL2+** aqueous solution (1-50 μM)

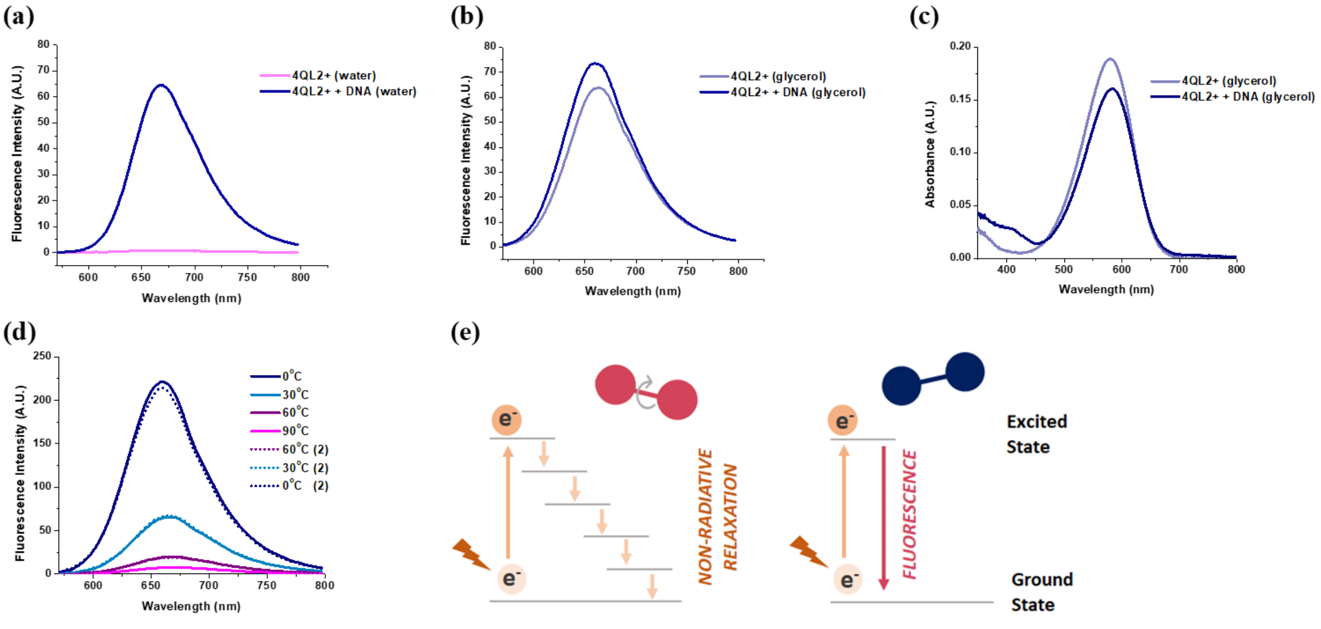

Figure S3. Fluorescence spectra of **4QL2+** (10 µM) in the absence and presence of DNA (450 µM, in bp) in (a) water (b) glycerol (c) Absorption spectra of **4QL2+** (10 µM) in the presence and absence of DNA (450 µM, in bp) in glycerol (d) Fluorescence spectra of **4QL2+** (10 µM) in glycerol (0-90 °C) (e) Illustration explained fluorescence change mechanism of the dye, λ_{ex} = 548 nm.

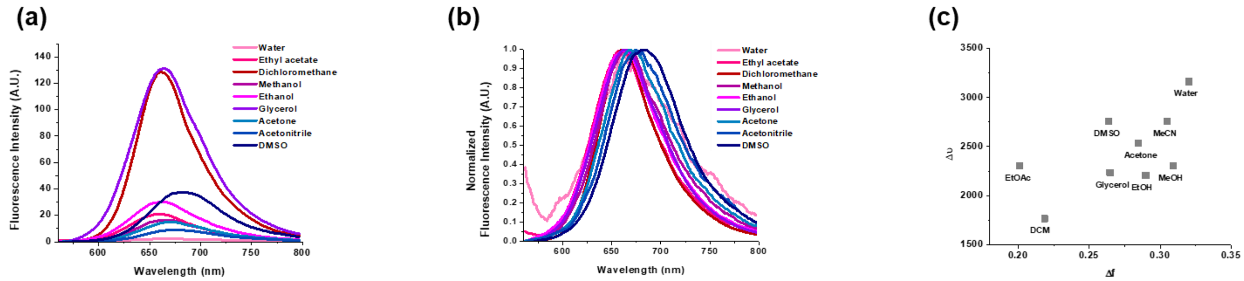

Figure S4. (a) Fluorescence spectra (b) Normalized fluorescence spectra of **4QL2+** (10 µM) in various solvents, λ_{ex} = 548 nm (c) Lippert-Mataga plot of the Stokes shift and orientation polarizability of the solvents surrounding the dye molecules of **4QL2+** dissolved in various solvents.

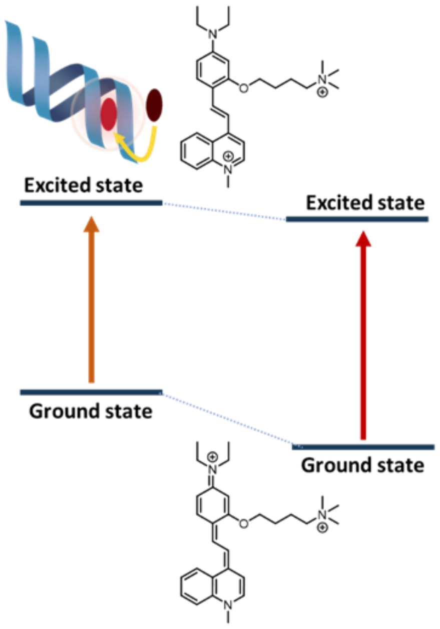

Figure S5. Illustration explained energy gaps of **4QL2+** in different polarity environment

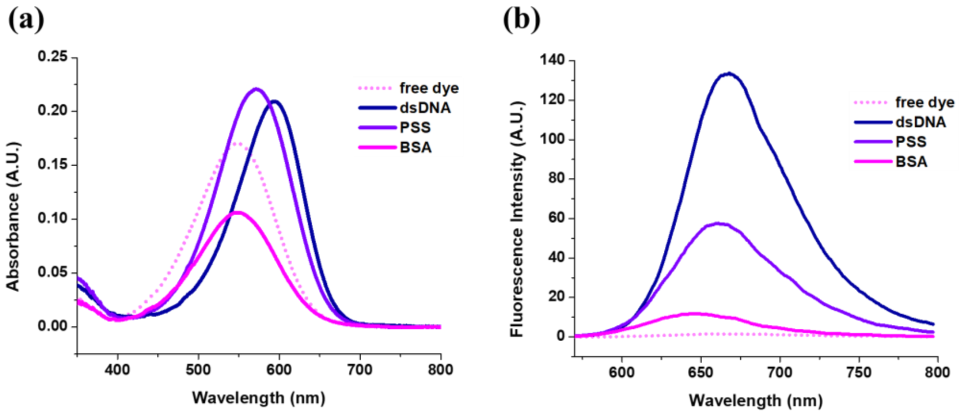

Figure S6. (a) Absorption and (b) fluorescence spectra of **4QL2+** (10 μM) in the presence of dsDNA, PSS, and BSA at the same concentration (100 μg/mL)

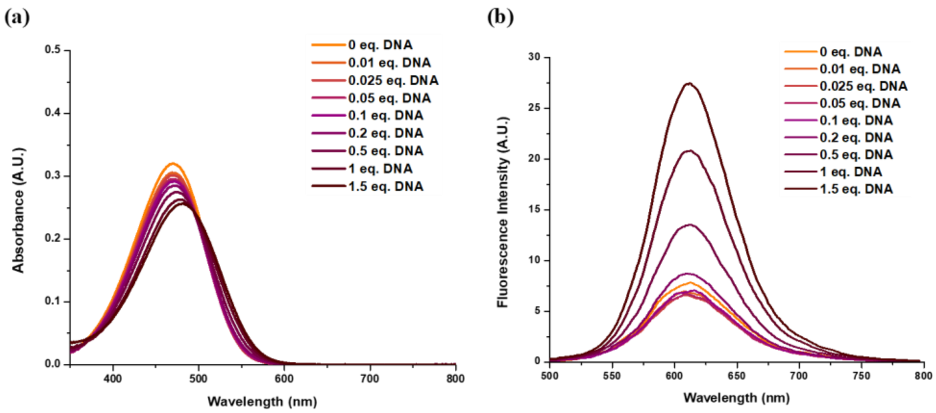

Figure S7. (a) UV-vis and (b) fluorescence titration of **PY+** (10 µM) with DNA (3 – 450 µM, in bp) in 10 mM sodium phosphate buffer pH 7.0; λ_{ex} = 480 nm.

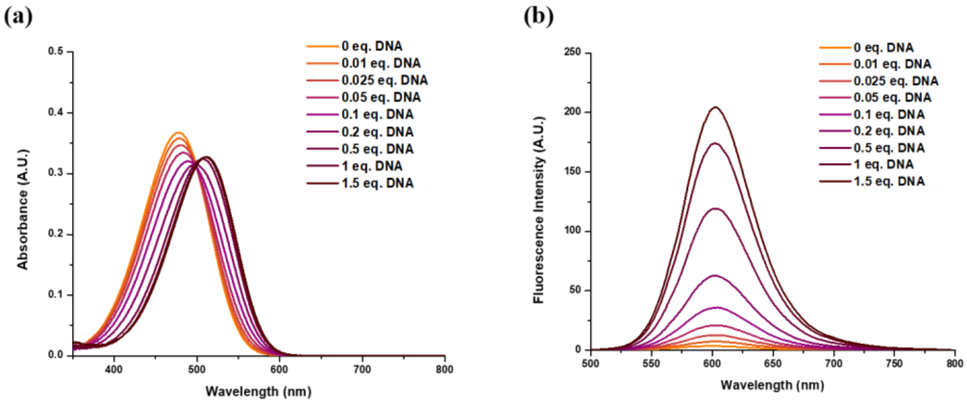

Figure S8. (a) UV-vis and (b) fluorescence titration of **PY2+(C2)** (10 µM) with DNA (3 – 450 µM, in bp) in 10 mM sodium phosphate buffer pH 7.0; λ_{ex} = 480 nm.

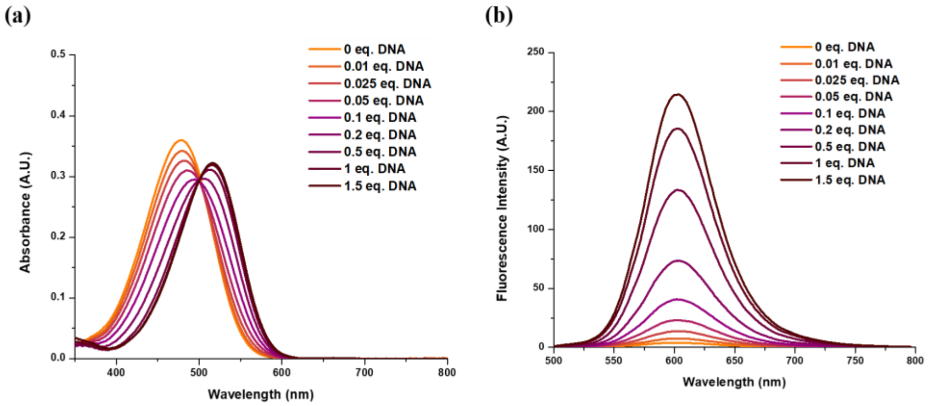

Figure S9. (a) UV-vis and (b) fluorescence titration of **PY2+(C3)** (10 µM) with DNA (3 – 450 µM, in bp) in 10 mM sodium phosphate buffer pH 7.0; λ_{ex} = 480 nm.

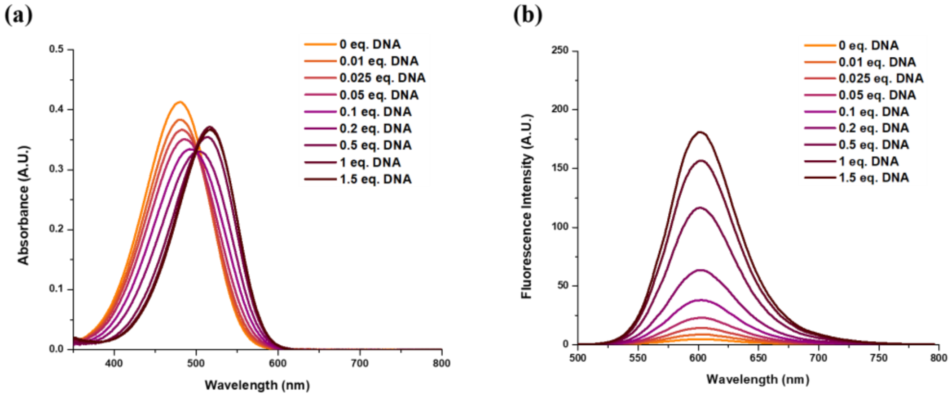

Figure S10. (a) UV-vis and (b) fluorescence titration of **PY2+(C4)** (10 µM) with DNA (3 – 450 µM, in bp) in 10 mM sodium phosphate buffer pH 7.0; λ_{ex} = 480 nm.

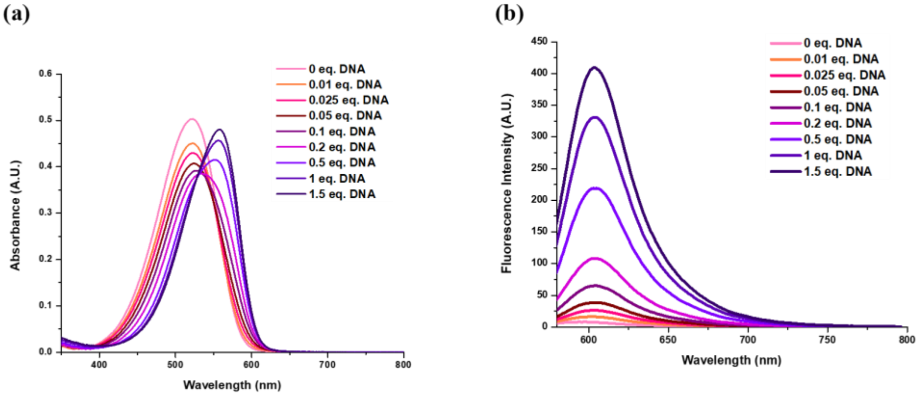

Figure S11. (a) UV-vis and (b) fluorescence titration of **BT+** ((a) 10 µM; (b) 1 µM) with DNA ((a) 3–450 µM; (b) 0.3–45 µM, in bp) in 10 mM sodium phosphate buffer pH 7.0; λ_{ex} = 565 nm.

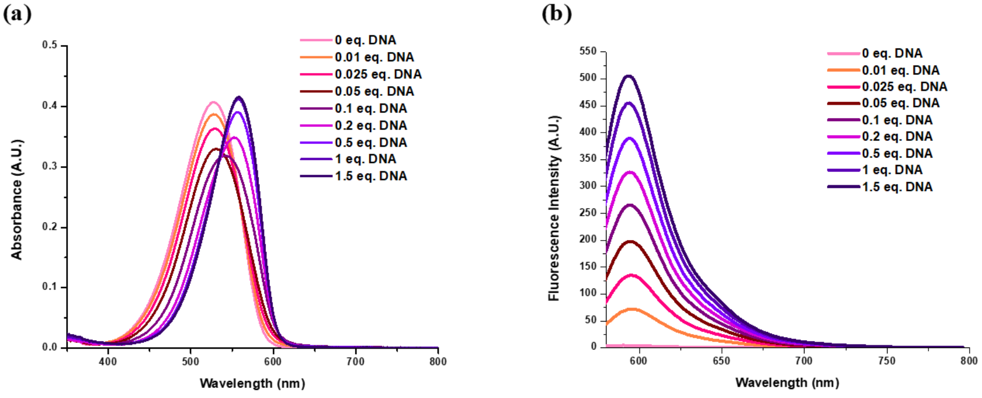

Figure S12. (a) UV-vis and (b) fluorescence titration of **BT2+(NEt$_2$)** ((a) 10 µM; (b) 1 µM) with DNA ((a) 3–450 µM; (b) 0.3–45 µM, in bp) in 10 mM sodium phosphate buffer pH 7.0; λ_{ex} = 565 nm.

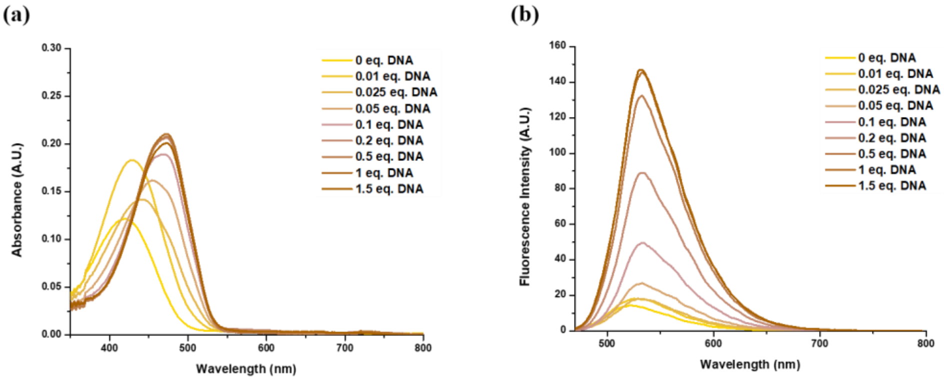

Figure S13. (a) UV-vis and (b) fluorescence titration of **BY2+(OMe)** (10 µM) with DNA (3 – 450 µM, in bp) in 10 mM sodium phosphate buffer pH 7.0; λ_{ex} = 450 nm.

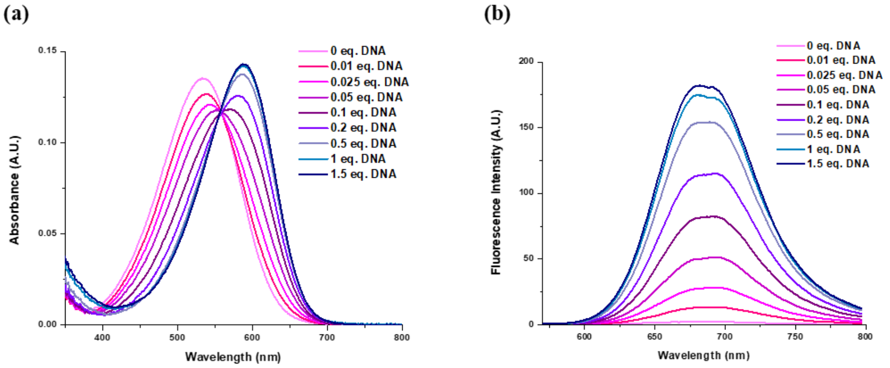

Figure S14. (a) UV-vis and (b) fluorescence titration of **4QL+** (10 µM) with DNA (3 – 450 µM, in bp) in 10 mM sodium phosphate buffer pH 7.0; λ_{ex} = 548 nm.

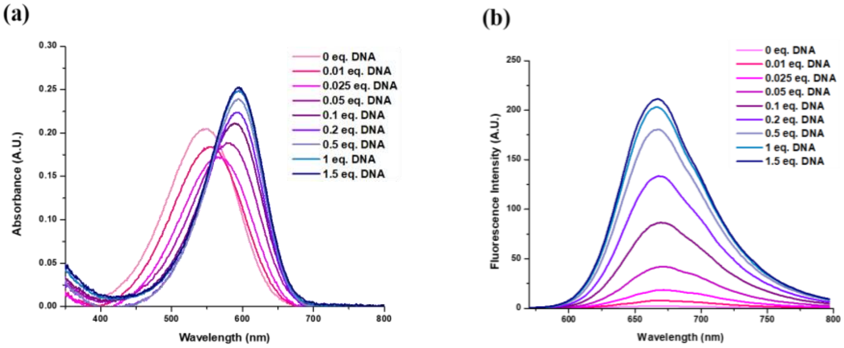

Figure S15. (a) UV-vis and (b) fluorescence titration of **4QL2+** (10 µM) with DNA (3 – 450 µM, in bp) in 10 mM sodium phosphate buffer pH 7.0; λ_{ex} = 548 nm.

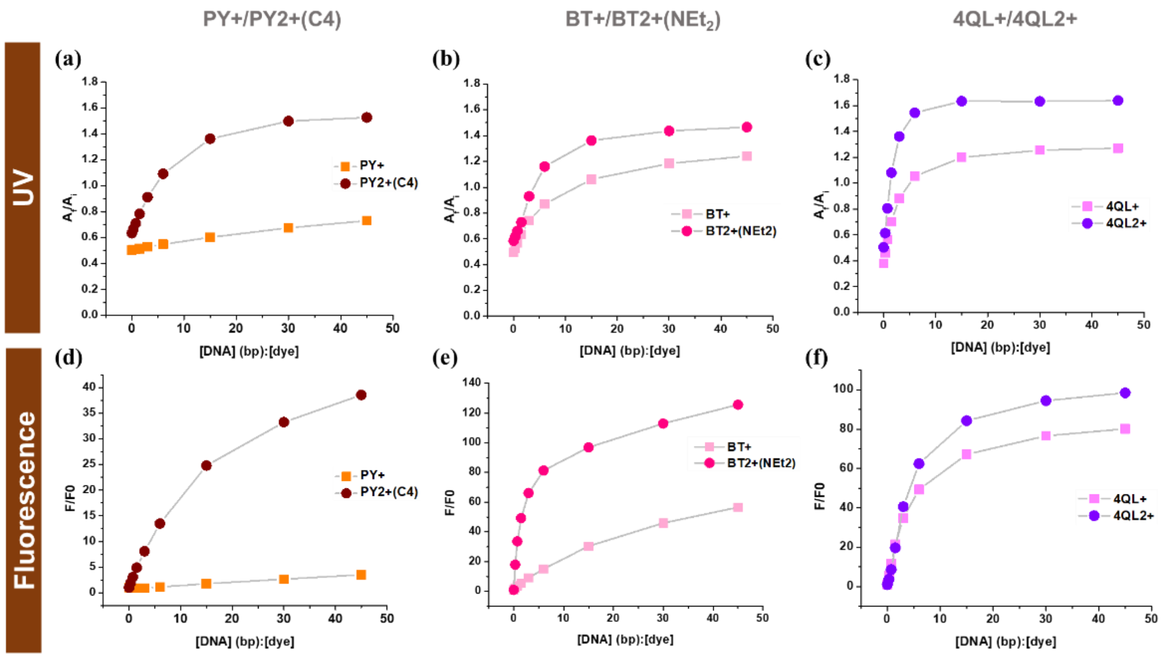

Figure S16. Plot of relative change of maximum absorption (expressed as A_f/A_i, which referred to the ratios between the absorption maxima of the free dye (A_i) and of the DNA-bound dye (A_f)) (a), (b), (c) and maximum fluorescence emission (expressed as F/F_0) (d), (c), (f) vs. concentration ratio of DNA (bp) and dyes. **Conditions:** [Dye] = 10 µM, [DNA (in bp)] = 3–450 µM, except for [**BT+**] = [**BT2+(NEt2)**] = 1 µM, [DNA (in bp)] = 0.3–45 µM in fluorescence measurement, **BT2+(OMe)** λ_{ex} = 450 nm, **PY+**, **PY2+(C4)** λ_{ex} = 480 nm, **BT+**, **BT2+(NEt$_2$)** λ_{ex} = 565 nm, **4QL+**, **4QL2+** λ_{ex} = 548 nm. All experiments were performed in 10 mM sodium phosphate buffer pH 7.0.

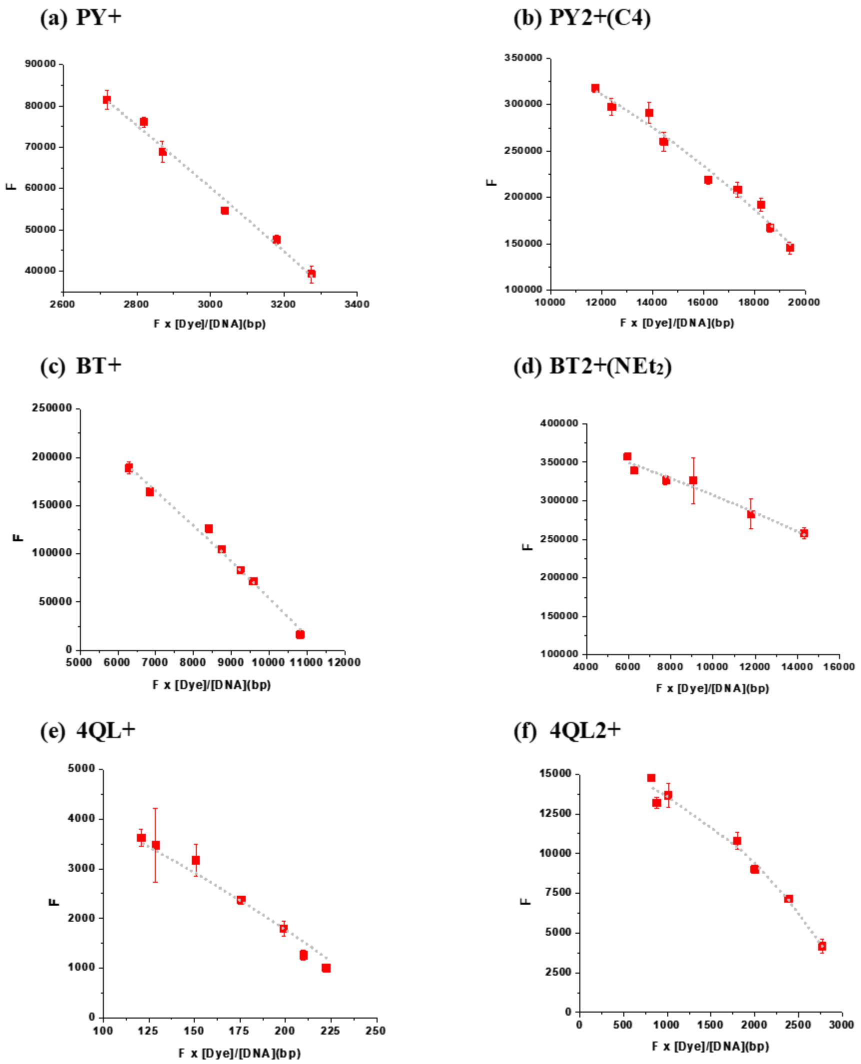

Figure S17. Plots of fluorescence intensity dependence on F×[Dye]/[DNA](bp) ratio (square) and its approximation by the modified Mcghee and von Hippel equation (equation 5) (dotted line) for (a) **PY+** (b) **PY2+(C4)** (c) **BT+** (d) **BT2+(NEt$_2$)** (e) **4QL+** (f) **4QL2+**

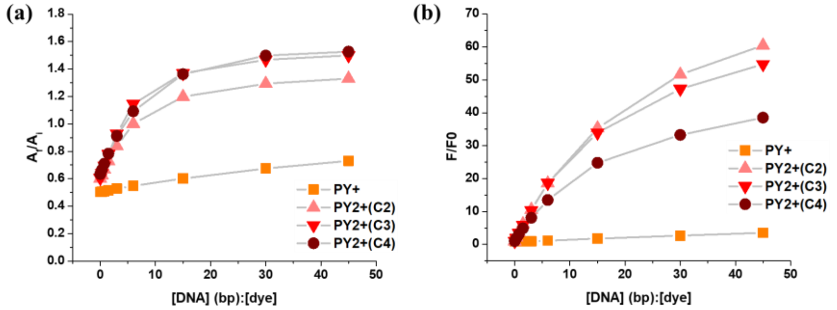

Figure S18. Plot of relative change of maximum absorption (expressed as A_f/A_i, which referred to the ratios between the absorption maxima of the free dye (A_i) and of the DNA-bound dye (A_f)) (a) and maximum fluorescence emission (expressed as F/F_0) (b) vs. concentration ratio of DNA (bp) and dyes with different linker lengths.

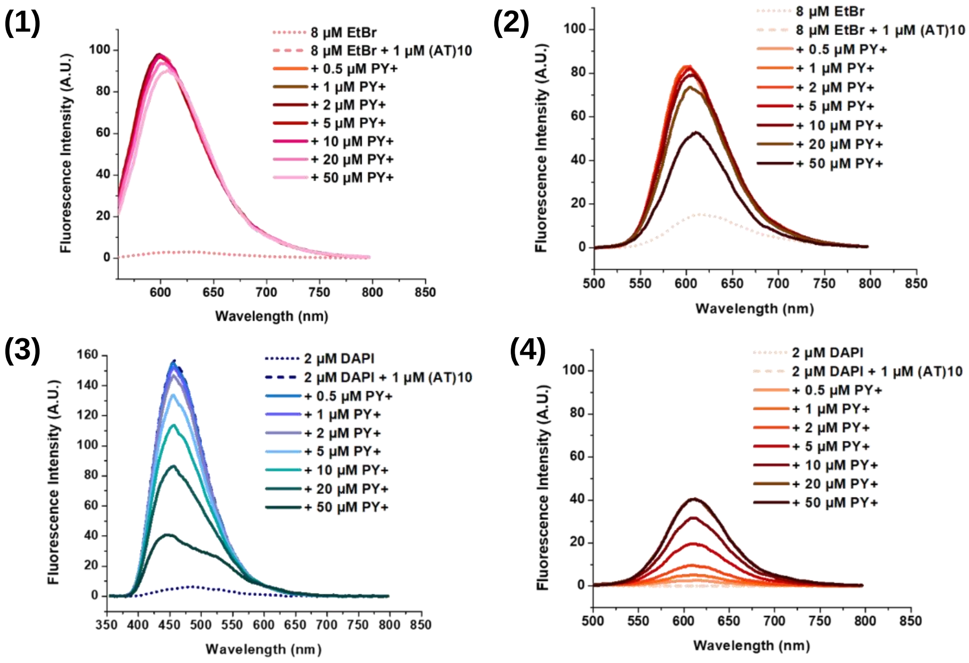

(a)

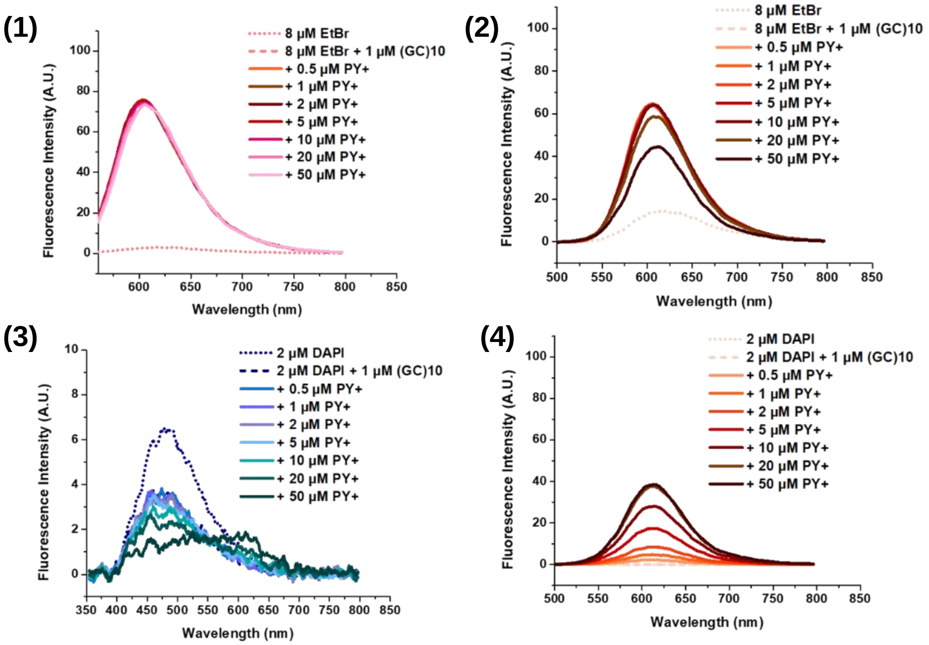

(b)

Figure S19. Fluorescent indicator displacement assay of EtBr (1,2) and DAPI (3,4) bound to DNA by of **PY+**. The excitation wavelengths of the indicators (λ_{ex} = 545 nm for EtBr, 341 nm for DAPI) were used for spectra 1 and 3, while the styryl dye excitation wavelength (λ_{ex} = 480 nm) was used for spectra 2 and 4. The DNA sequences used were (a) $d(AT)_{10}$ and (b) $d(GC)_{10}$. The assays were performed in 10 mM sodium phosphate buffer pH 7.0.

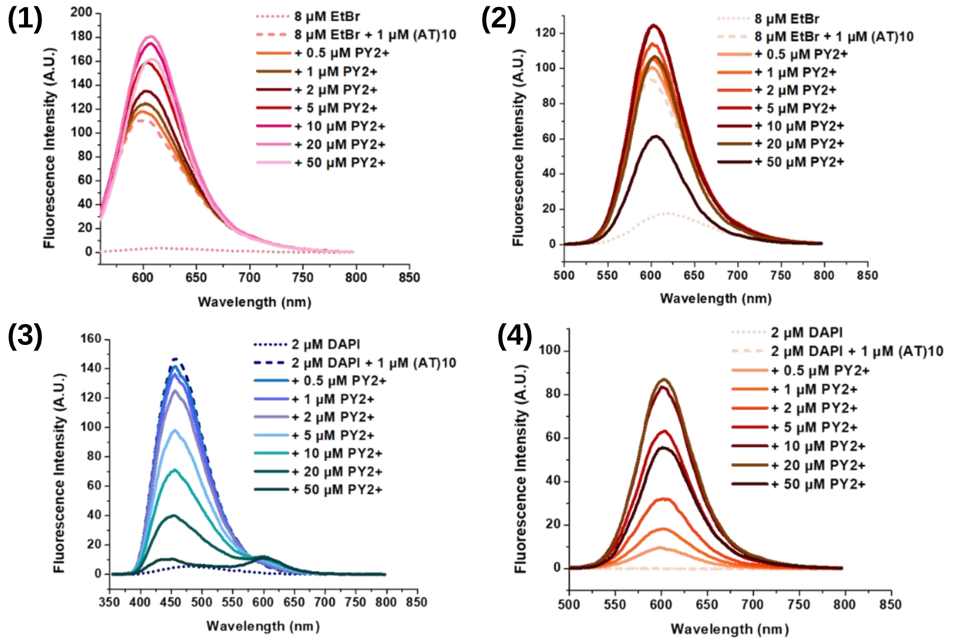

(a)

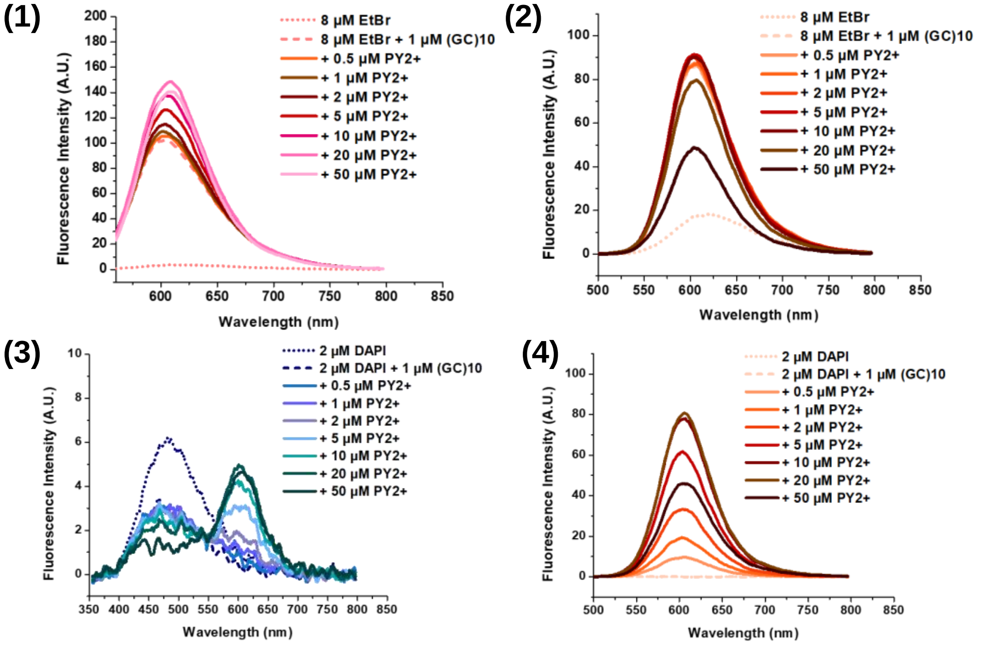

(b)

Figure S20. Fluorescent indicator displacement assay of EtBr (1,2) and DAPI (3,4) bound to DNA by of **PY2+(C4)**. The excitation wavelengths of the indicators (λ_{ex} = 545 nm for EtBr, 341 nm for DAPI) were used for spectra 1 and 3, while the styryl dye excitation wavelength (λ_{ex} = 480 nm) was used for spectra 2 and 4. The DNA sequences used were (a) d(AT)$_{10}$ and (b) d(GC)$_{10}$. The assays were performed in 10 mM sodium phosphate buffer pH 7.0.

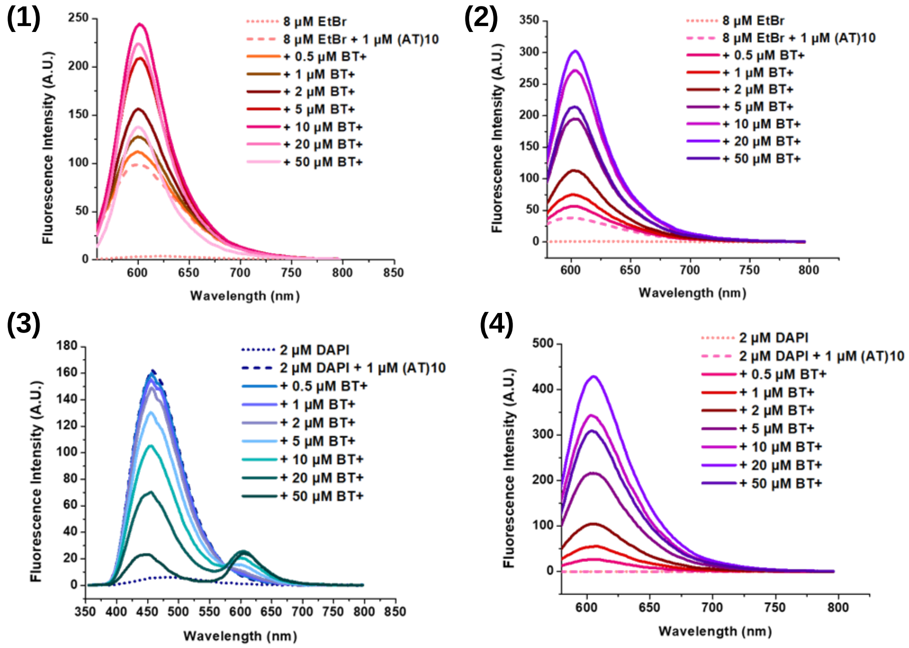

(a)

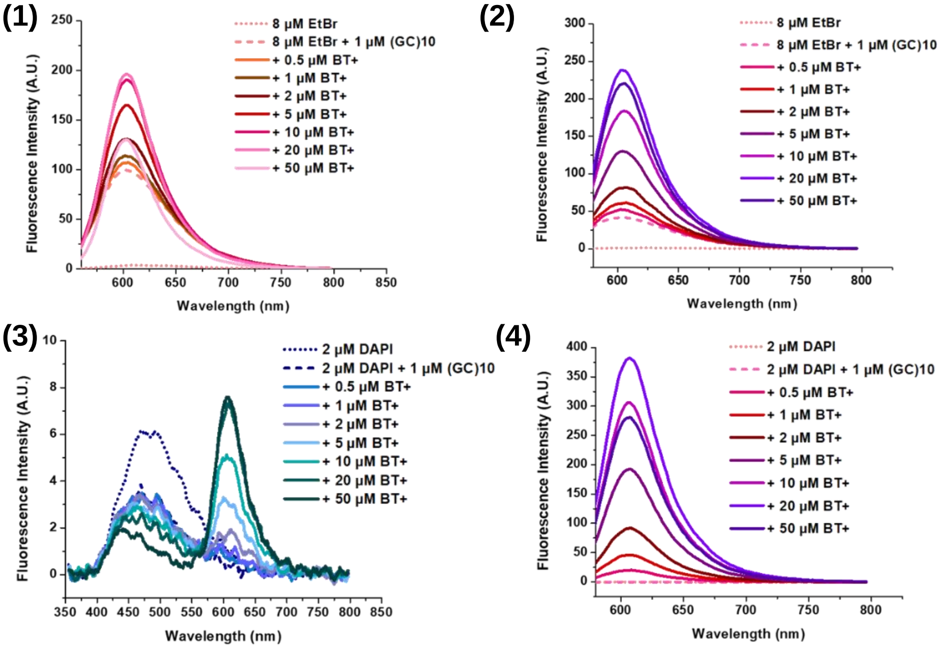

(b)

Figure S21. Fluorescent indicator displacement assay of EtBr (1,2) and DAPI (3,4) bound to DNA by of **BT+**. The excitation wavelengths of the indicators (λ_{ex} = 545 nm for EtBr, 341 nm for DAPI) were used for spectra 1 and 3, while the styryl dye excitation wavelength (λ_{ex} = 565 nm) was used for spectra 2 and 4. The DNA sequences used were (a) d(AT)$_{10}$ and (b) d(GC)$_{10}$. The assays were performed in 10 mM sodium phosphate buffer pH 7.0.

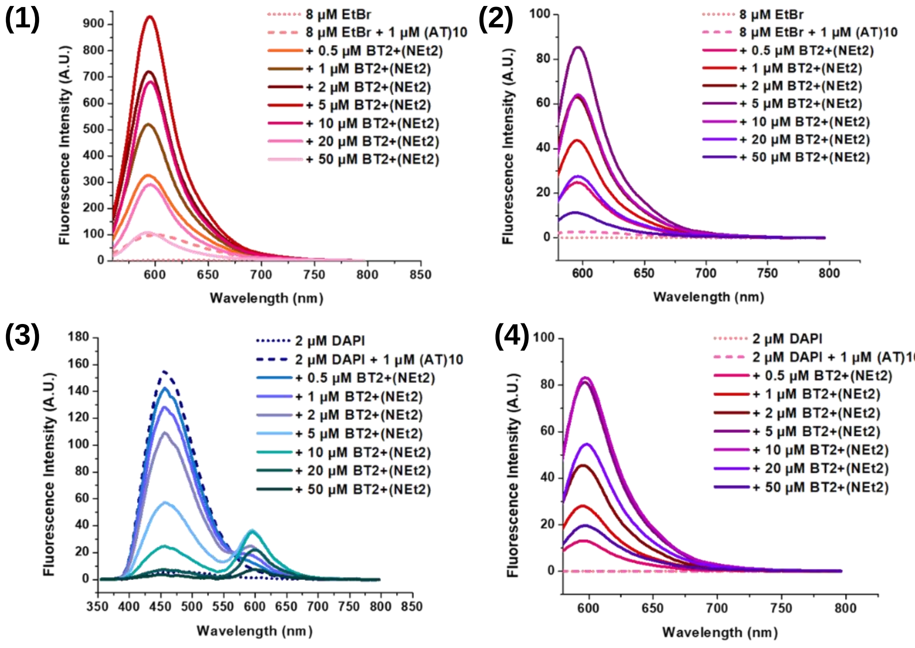

(a)

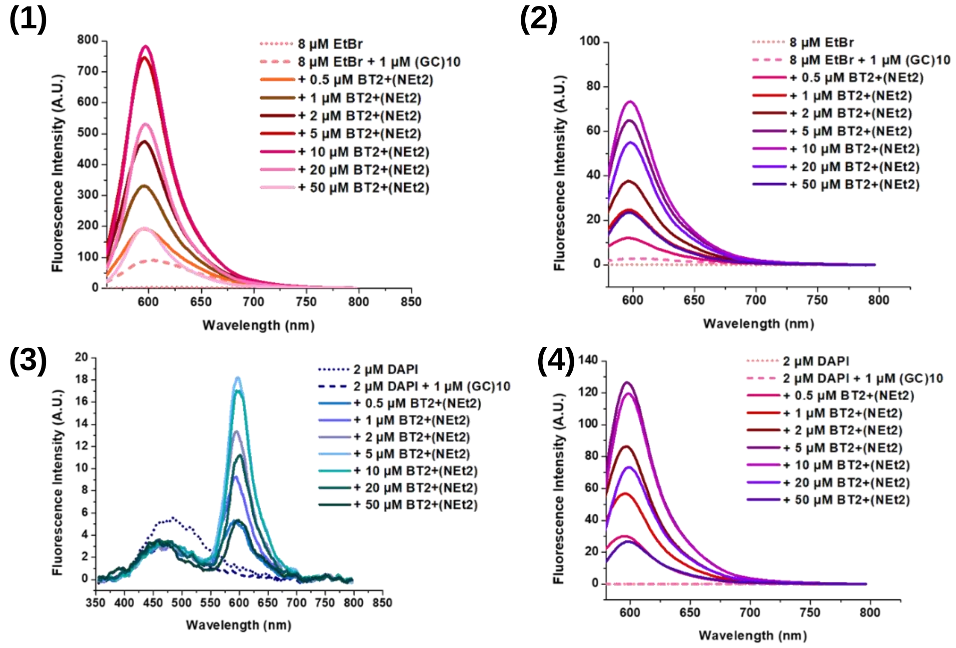

(b)

Figure S22. Fluorescent indicator displacement assay of EtBr (1,2) and DAPI (3,4) bound to DNA by of **BT2+(NEt$_2$)**. The excitation wavelengths of the indicators (λ_{ex} = 545 nm for EtBr, 341 nm for DAPI) were used for spectra 1 and 3, while the styryl dye excitation wavelength (λ_{ex} = 565 nm) was used for spectra 2 and 4. The DNA sequences used were (a) d(AT)$_{10}$ and (b) d(GC)$_{10}$. The assays were performed in 10 mM sodium phosphate buffer pH 7.0.

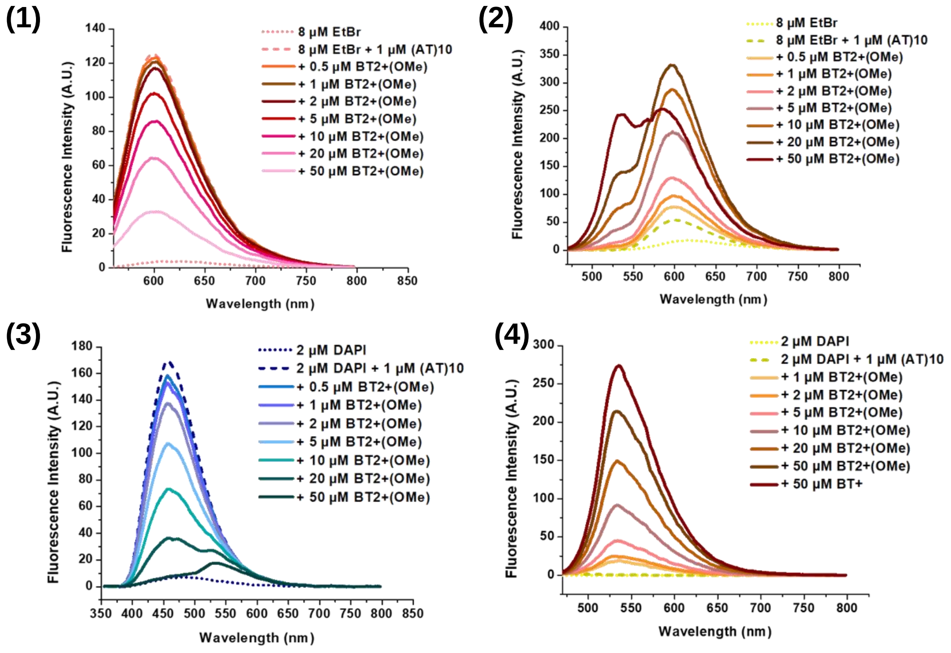

(a)

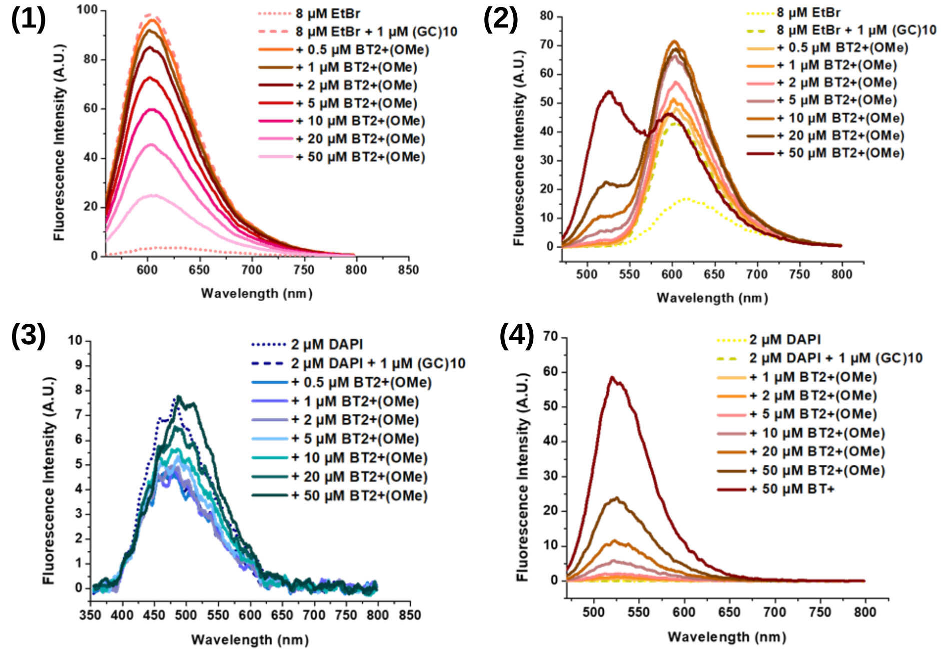

(b)

Figure S23. Fluorescent indicator displacement assay of EtBr (1,2) and DAPI (3,4) bound to DNA by of **BT2+(OMe)**. The excitation wavelengths of the indicators (λ_{ex} = 545 nm for EtBr, 341 nm for DAPI) were used for spectra 1 and 3, while the styryl dye excitation wavelength (λ_{ex} = 450 nm) was used for spectra 2 and 4. The DNA sequences used were (a) $d(AT)_{10}$ and (b) $d(GC)_{10}$. The assays were performed in 10 mM sodium phosphate buffer pH 7.0.

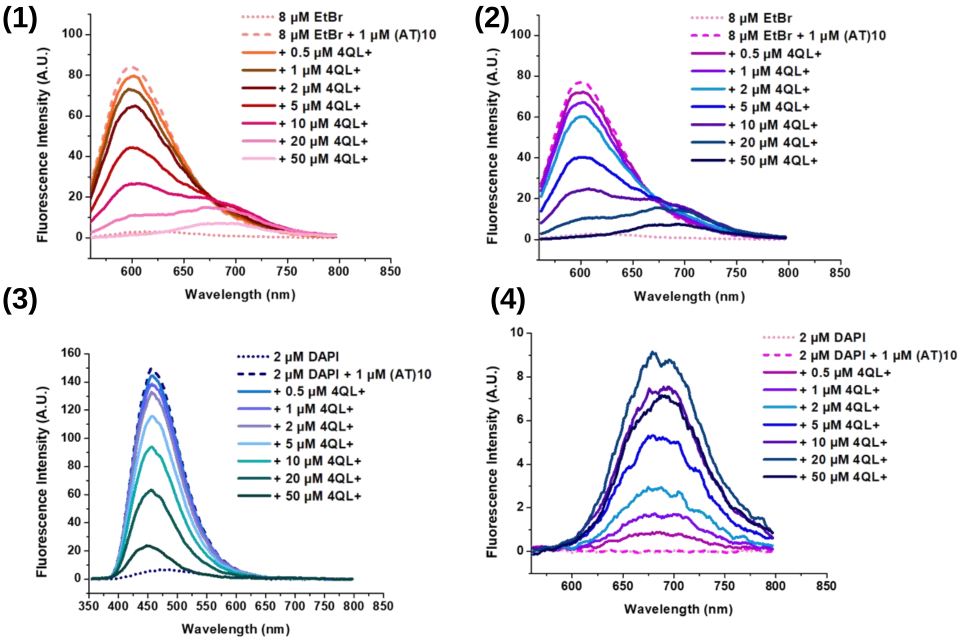

(a)

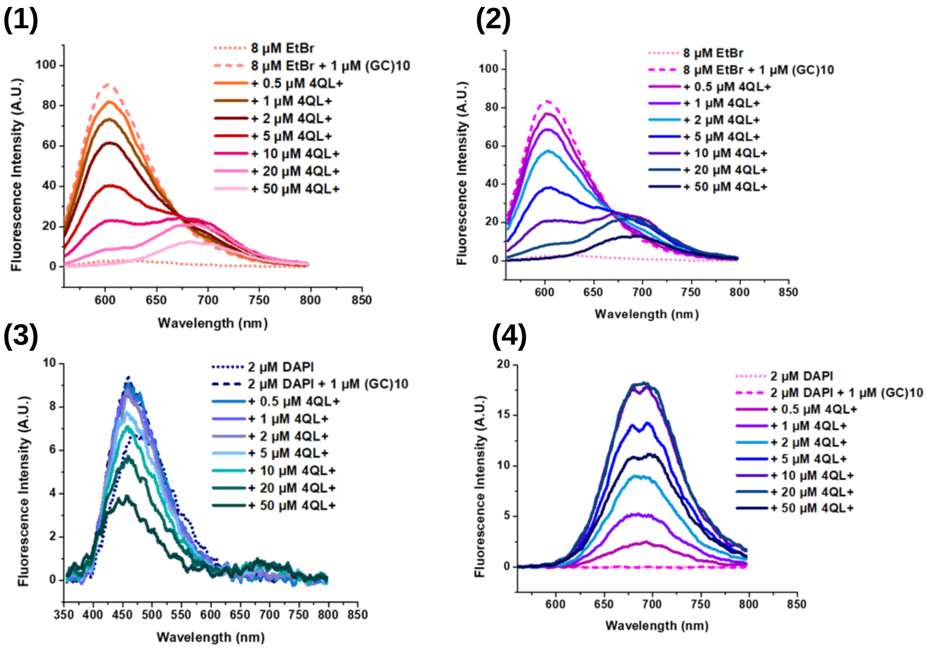

(b)

Figure S24. Fluorescent indicator displacement assay of EtBr (1,2) and DAPI (3,4) bound to DNA by of **4QL+**. The excitation wavelengths of the indicators (λ_{ex} = 545 nm for EtBr, 341 nm for DAPI) were used for spectra 1 and 3, while the styryl dye excitation wavelength (λ_{ex} = 548 nm) was used for spectra 2 and 4. The DNA sequences used were (a) d(AT)$_{10}$ and (b) d(GC)$_{10}$. The assays were performed in 10 mM sodium phosphate buffer pH 7.0.

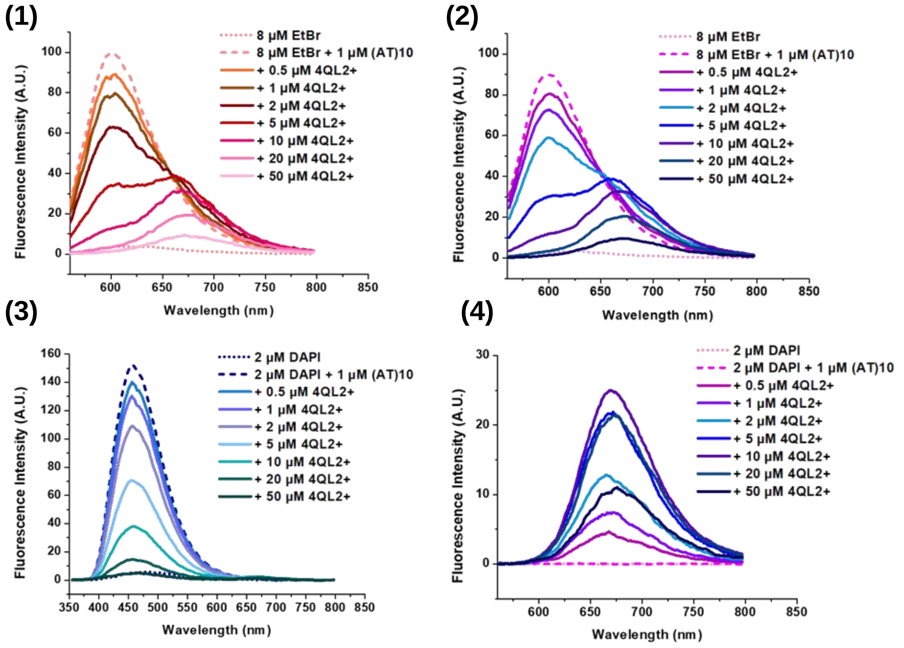

(a)

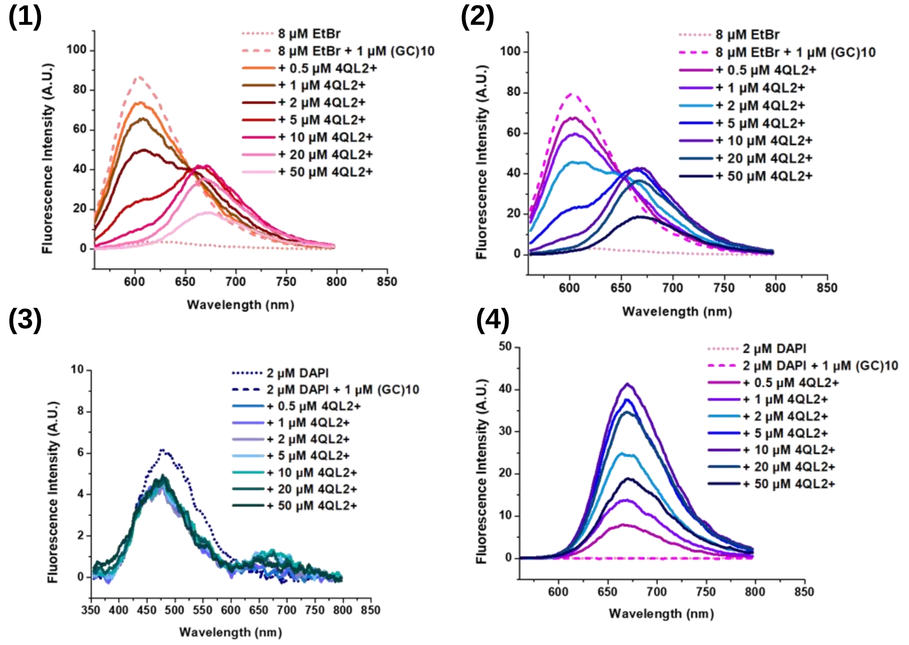

(b)

Figure S25. Fluorescent indicator displacement assay of EtBr (1,2) and DAPI (3,4) bound to DNA by of **4QL2+**. The excitation wavelengths of the indicators (λ_{ex} = 545 nm for EtBr, 341 nm for DAPI) were used for spectra 1 and 3, while the styryl dye excitation wavelength (λ_{ex} = 548 nm) was used for spectra 2 and 4. The DNA sequences used were (a) d(AT)$_{10}$ and (b) d(GC)$_{10}$. The assays were performed in 10 mM sodium phosphate buffer pH 7.0.

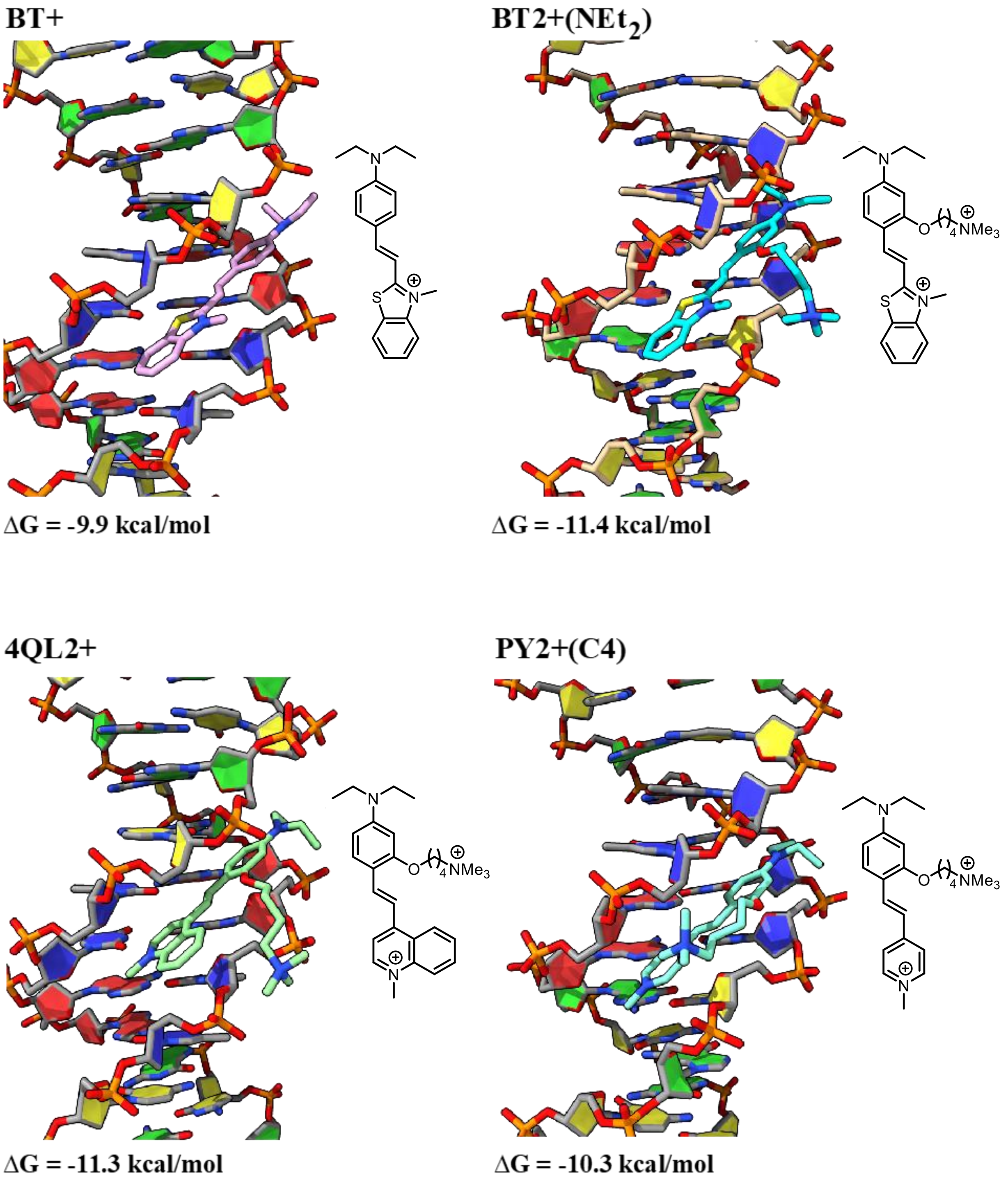

Figure S26. Molecular docking of styryl dyes on a minor groove DNA (4C64) model

BT+

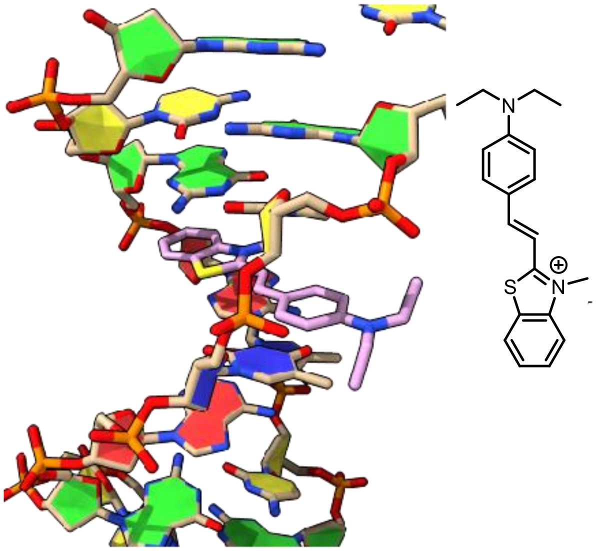

ΔG = -7.4 kcal/mol

BT2+(NEt$_2$)

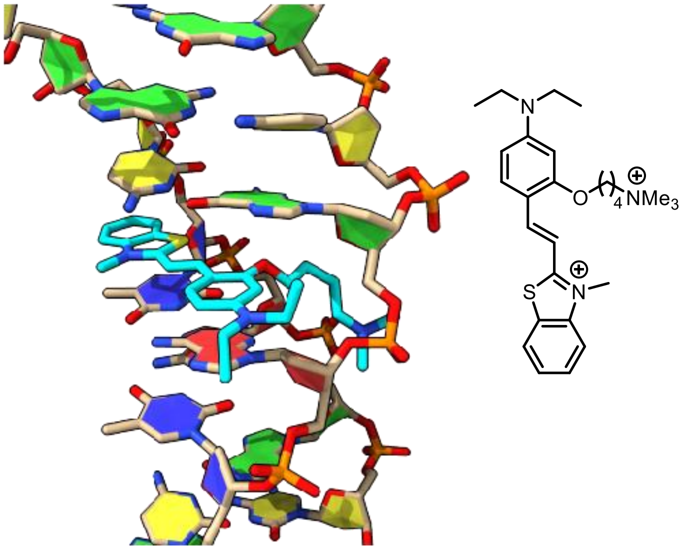

ΔG = -9.0 kcal/mol

4QL2+

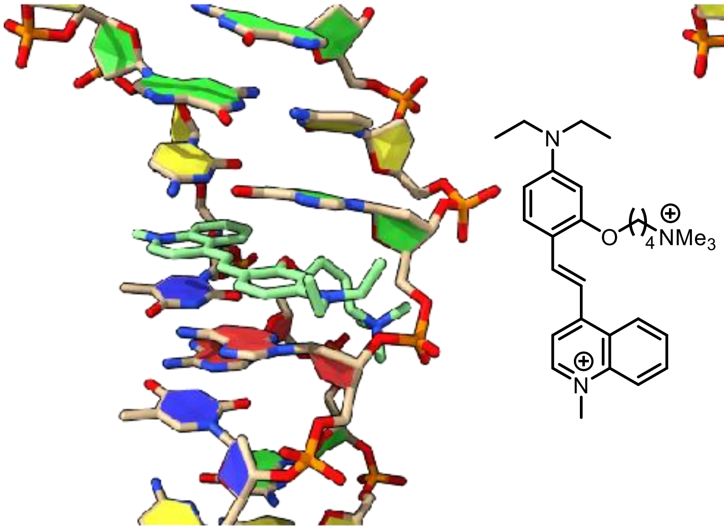

ΔG = -9.5 kcal/mol

PY2+(C4)

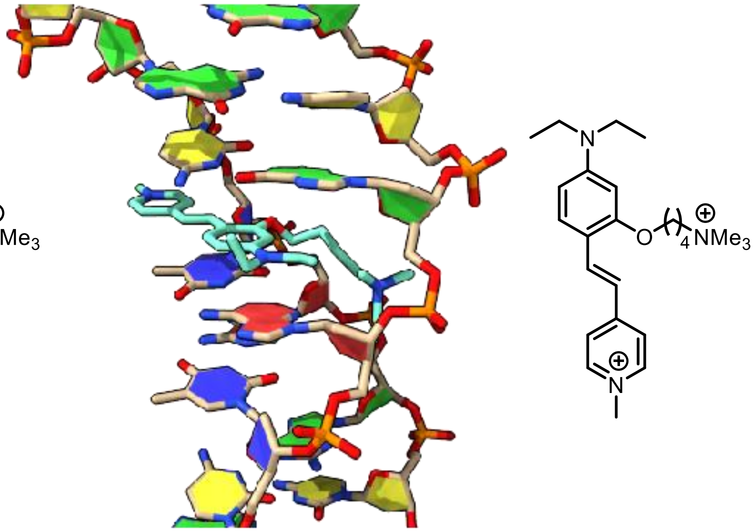

ΔG = -8.4 kcal/mol

Figure S27. Molecular docking of styryl dyes at an intercalative site of DNA (108D) model

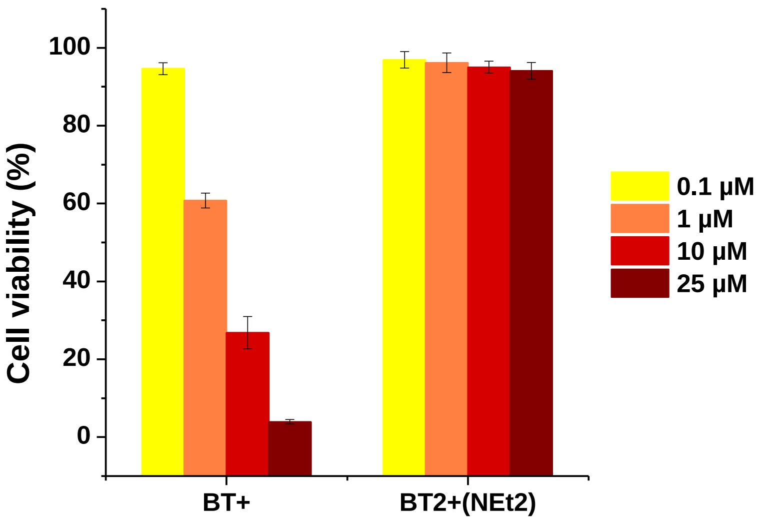

Figure S28. Cell cytotoxicity study of **BT+** and **BT2+(NEt$_2$)** in HeLa cells using MTT assay. HeLa cells were treated with the dyes at the specified concentrations for 24 h prior to determination of cell viability by MTT assay.

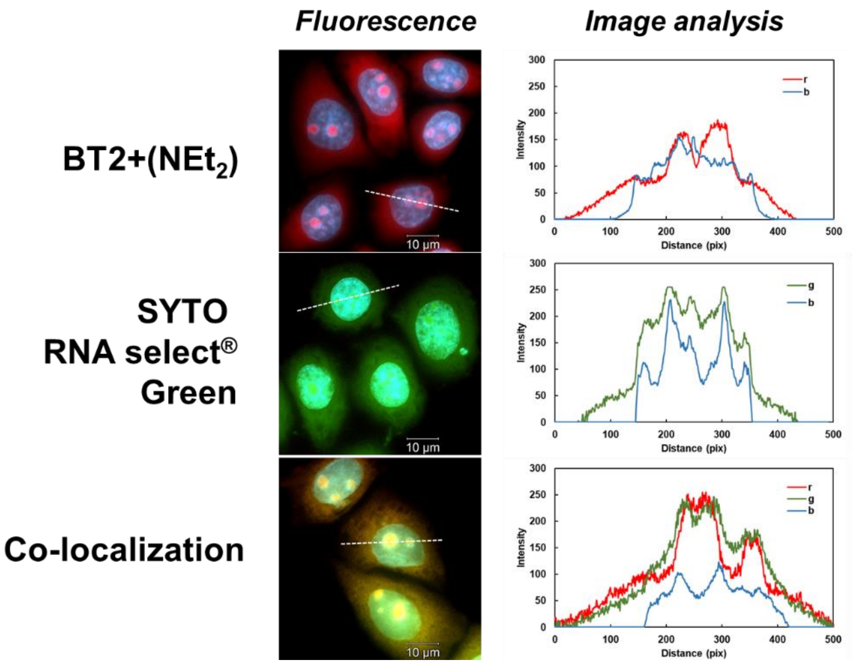

Figure S29. Fluorescence image analysis of HeLa cells treated with the **BT2+(NEt$_2$)** dye at 20 µM, colocalized with SYTO RNA select® Green, and co-stained with DAPI. Scale bars = 10 µm. Image analysis was performed by ImageJ to confirm the colocalization of the staining dyes (Pearson's coefficient = 0.638).

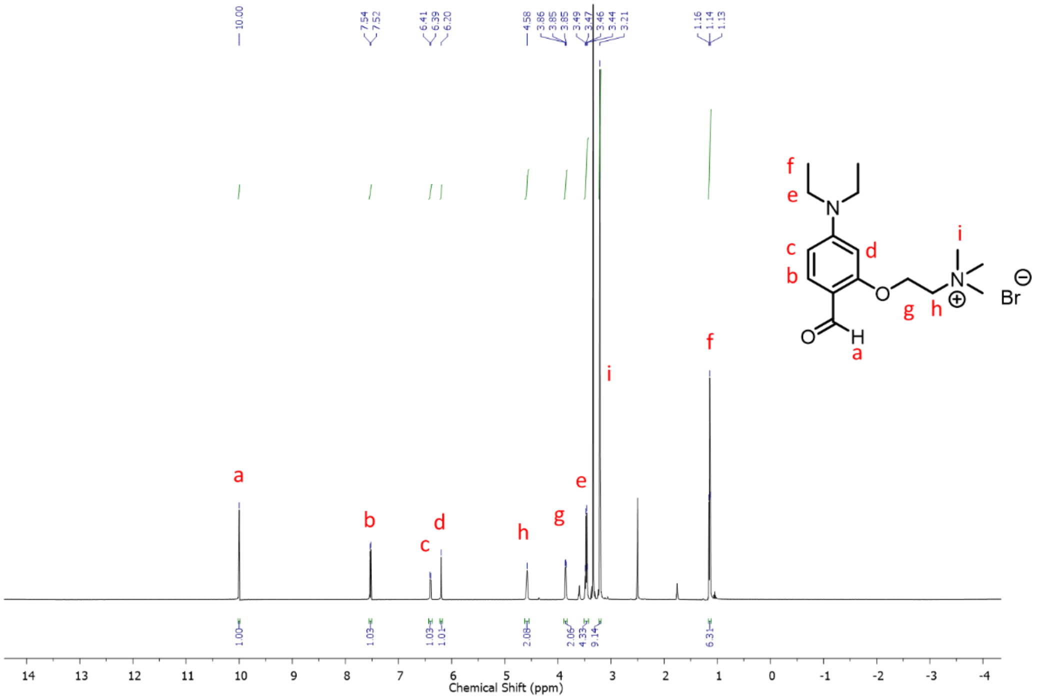

Figure S30. ^{1}H (500 MHz, DMSO-d_6) NMR spectrum of 2-(5-(diethylamino)-2-formylphenoxy)-*N*,*N*,*N*-trimethylethan-1-aminium bromide

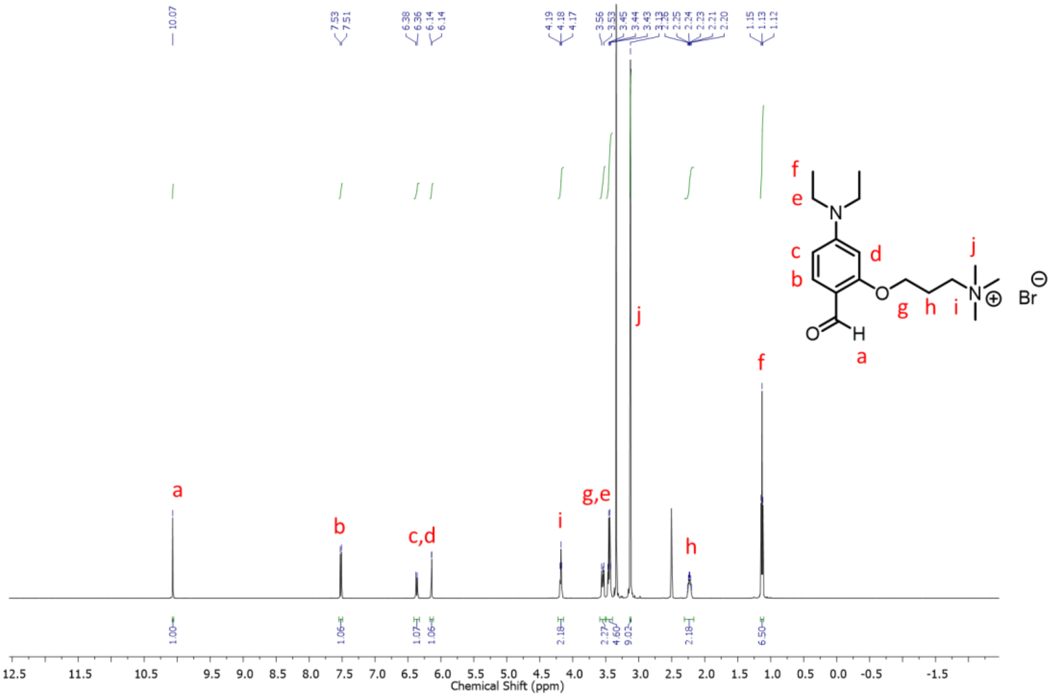

Figure S31. ^{1}H (500 MHz, DMSO-d_6) NMR spectrum of 3-(5-(diethylamino)-2-formylphenoxy)-*N*,*N*,*N*-trimethylpropan-1-aminium bromide

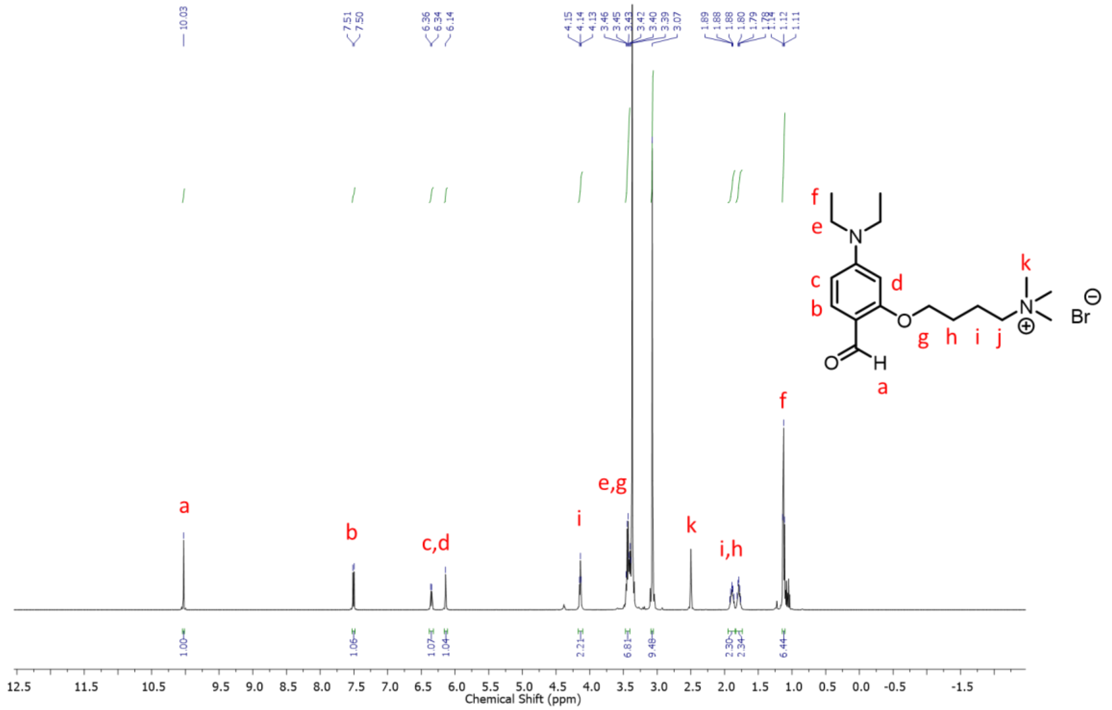

Figure S32. ^{1}H (500 MHz, DMSO-d_6) NMR spectrum of 4-(5-(diethylamino)-2-formylphenoxy)-*N*,*N*,*N*-trimethylbutan-1-aminium bromide

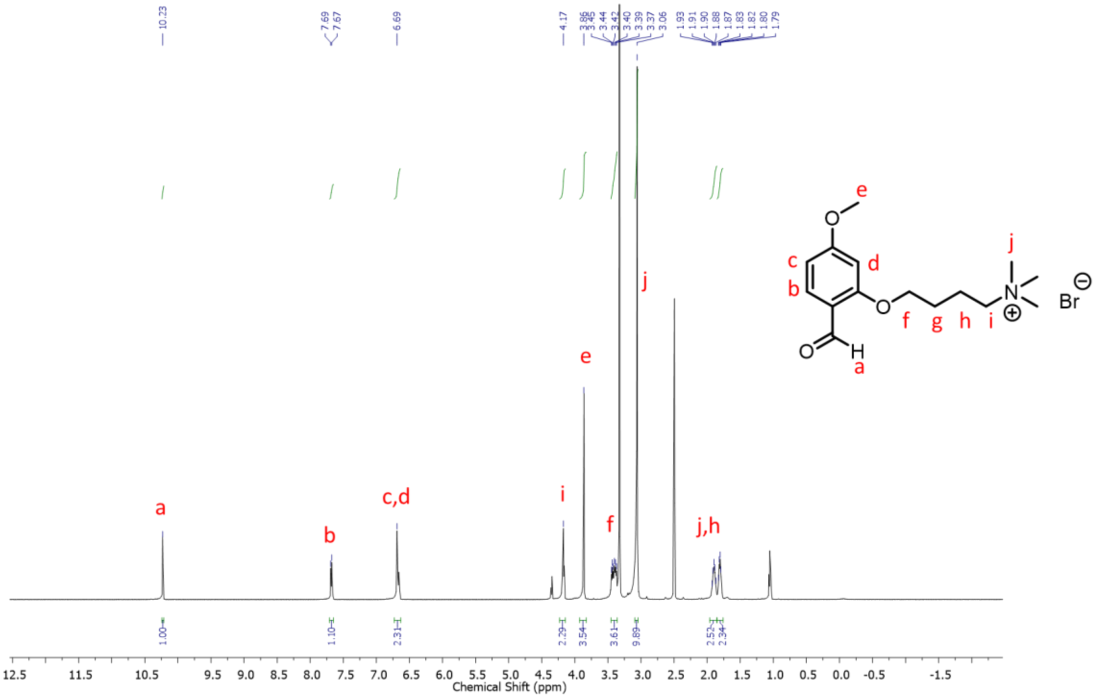

Figure S33. ^{1}H (500 MHz, DMSO-d_6) NMR spectrum of 4-(2-formyl-5-methoxyphenoxy)-*N*,*N*,*N*-trimethylbutan-1-aminium bromide

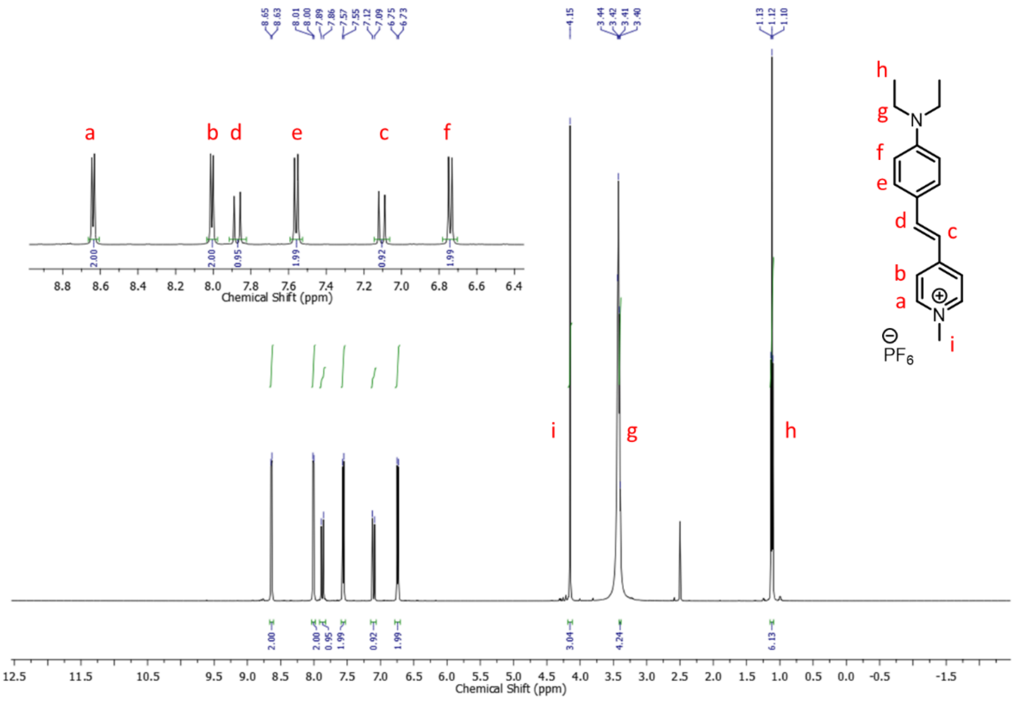

(a)

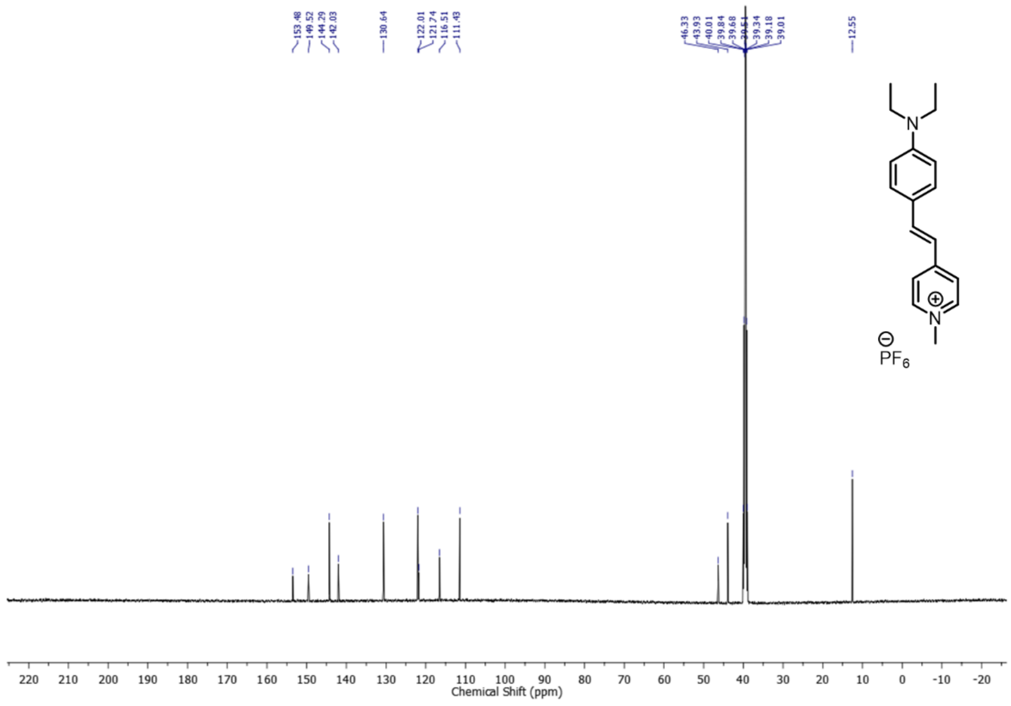

(b)

Figure S34. ^{1}H (500 MHz, DMSO-d_6) (a) and ^{13}C (126 MHz, DMSO-d_6) (b) NMR spectra of compound **PY+**

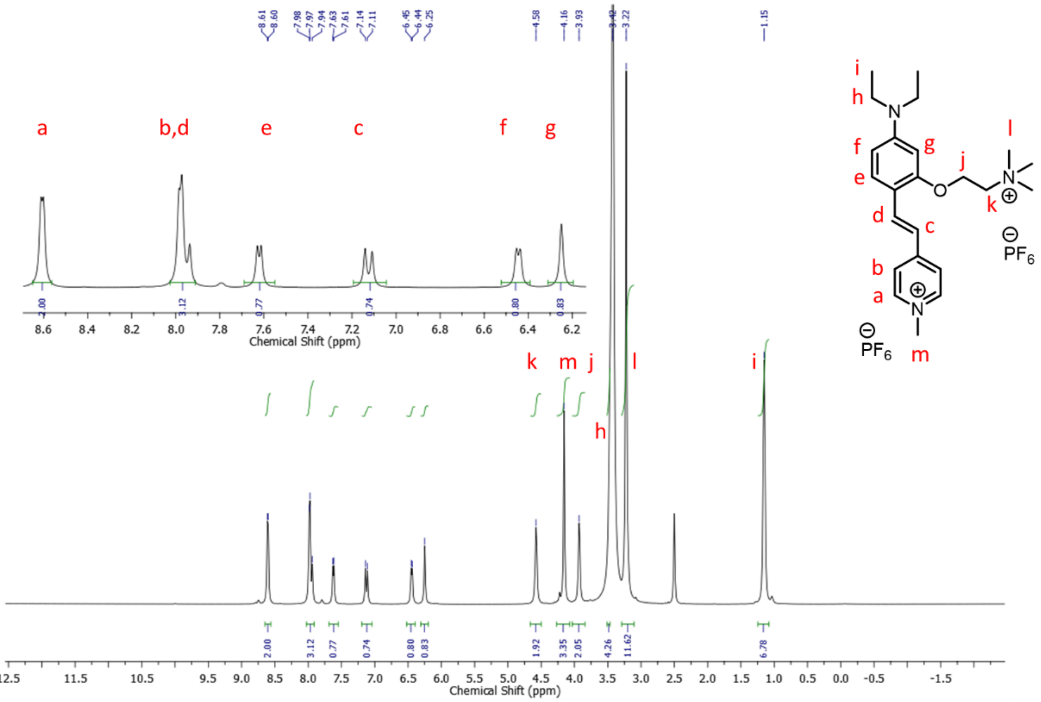

(a)

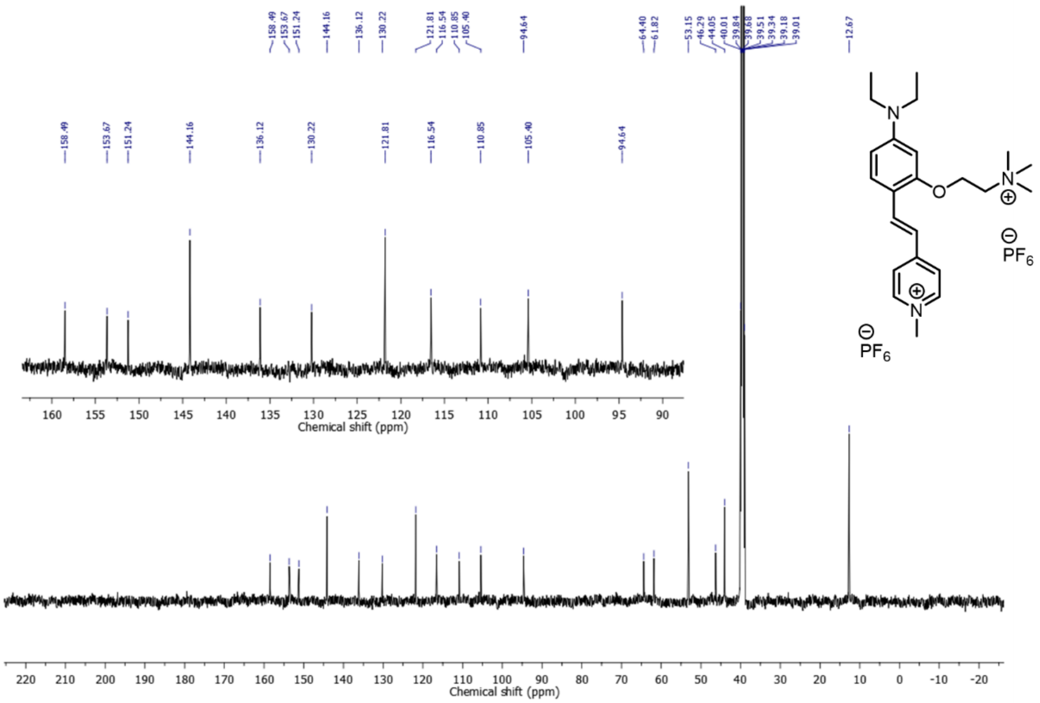

(b)

Figure S35. ^{1}H (500 MHz, DMSO-d_6) (a) and ^{13}C (126 MHz, DMSO-d_6) (b) NMR spectra of compound **PY2+(C2)**

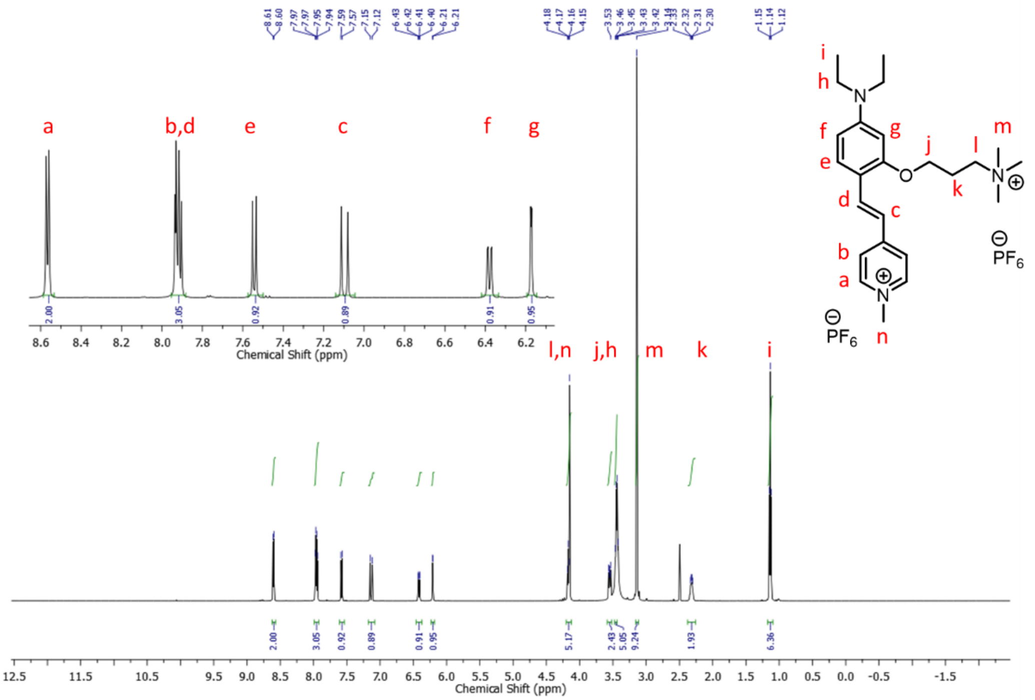

(a)

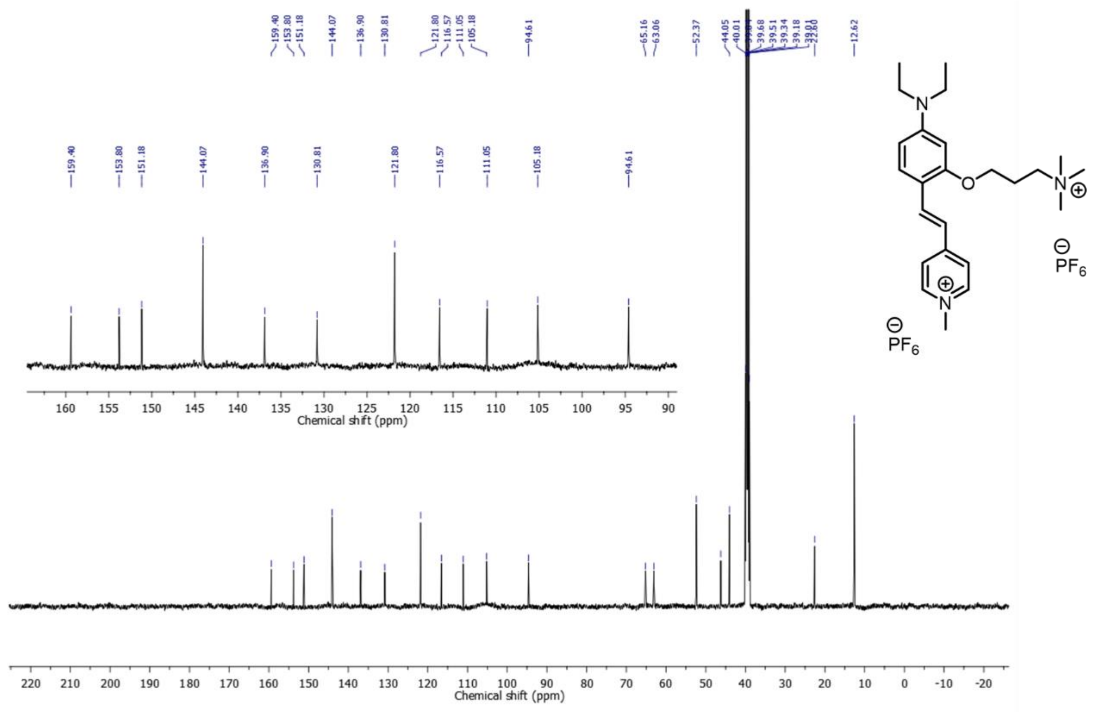

(b)

Figure S36. ^{1}H (500 MHz, DMSO-d_6) (a) and ^{13}C (126 MHz, DMSO-d_6) (b) NMR spectra of compound **PY2+(C3)**

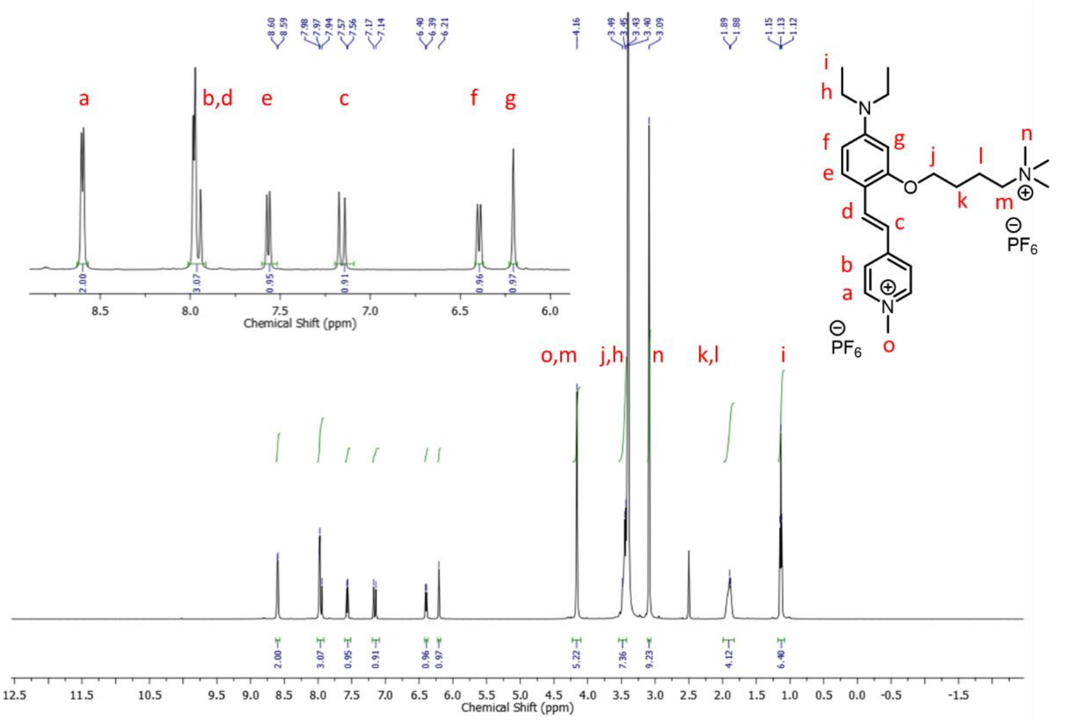

(a)

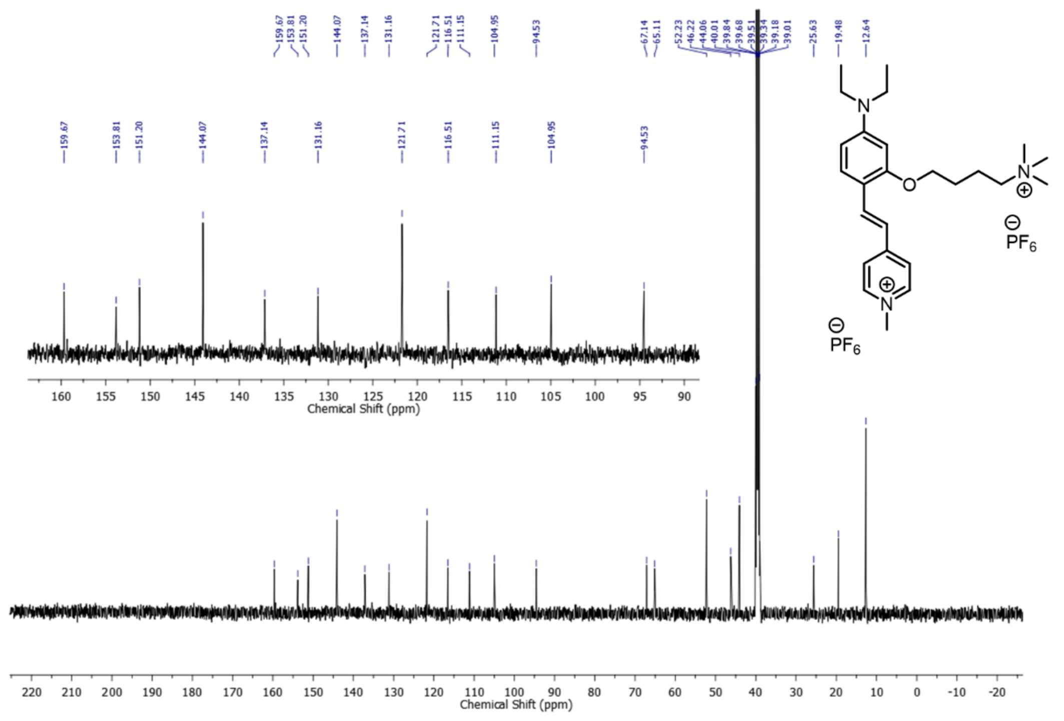

(b)

Figure S37. ^{1}H (500 MHz, DMSO-d_6) (a) and ^{13}C (126 MHz, DMSO-d_6) (b) NMR spectra of compound **PY2+(C4)**

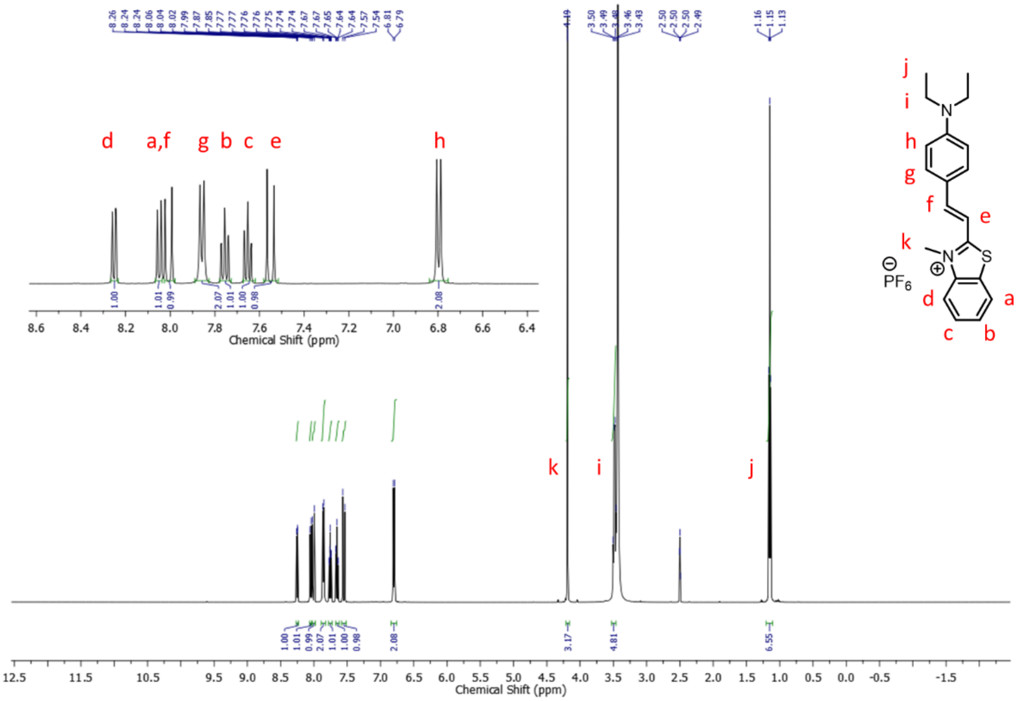

(a)

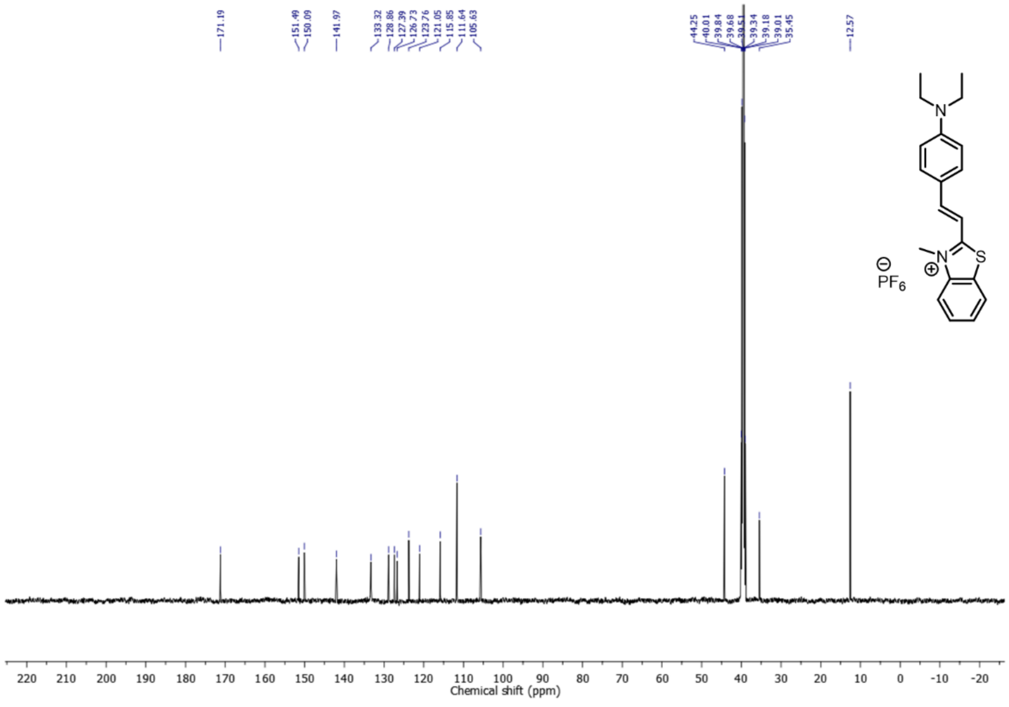

(b)

Figure S38. ^{1}H (500 MHz, DMSO-d_6) (a) and ^{13}C (126 MHz, DMSO-d_6) (b) NMR spectra of compound **BT+**

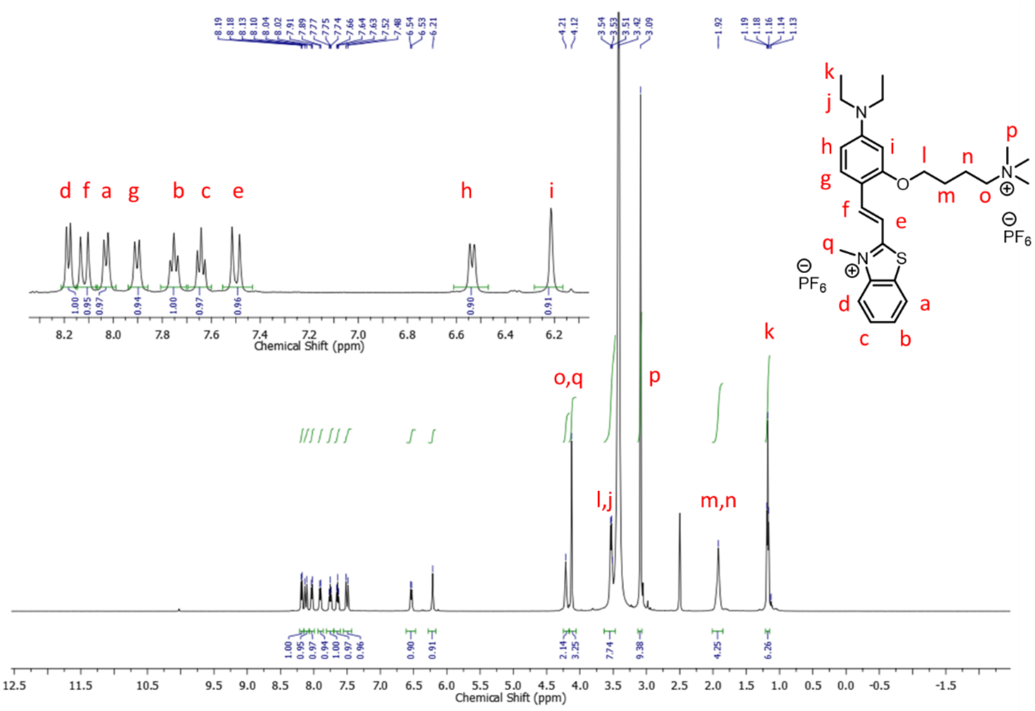

(a)

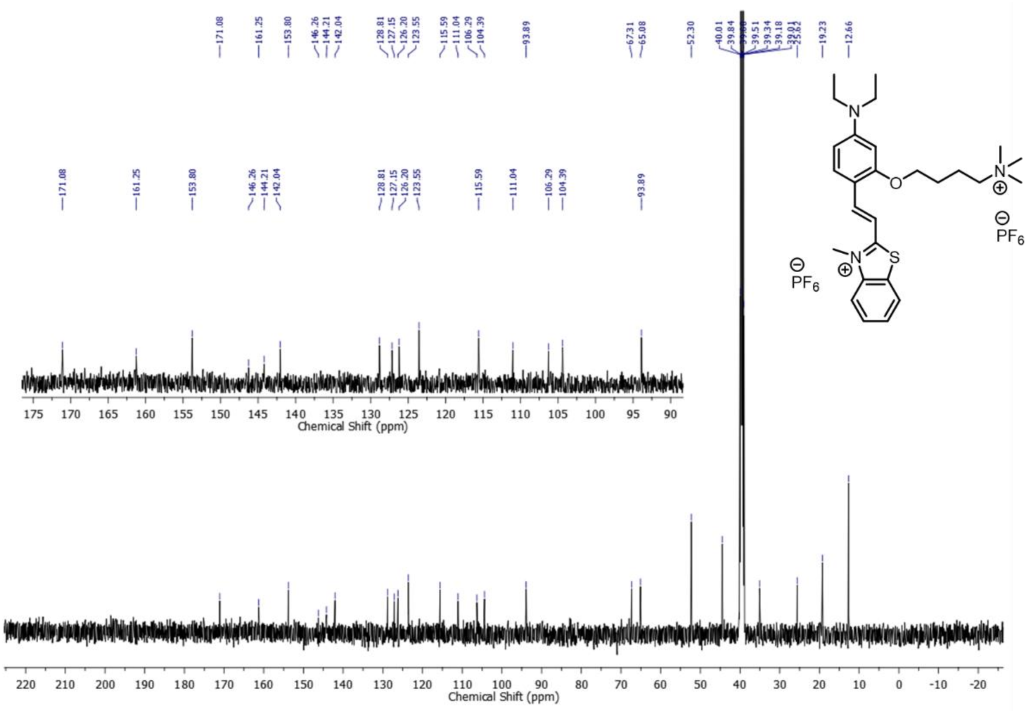

(b)

Figure S39. ^{1}H (500 MHz, DMSO-d_6) (a) and ^{13}C (126 MHz, DMSO-d_6) (b) NMR spectra of compound **BT2+(NEt$_2$)**

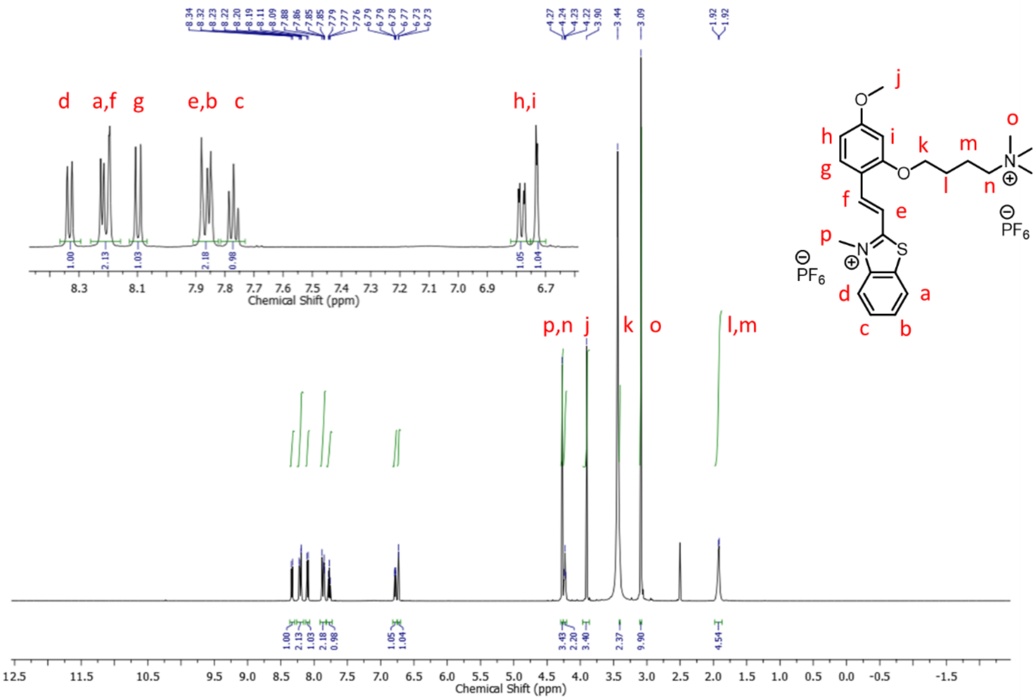

(a)

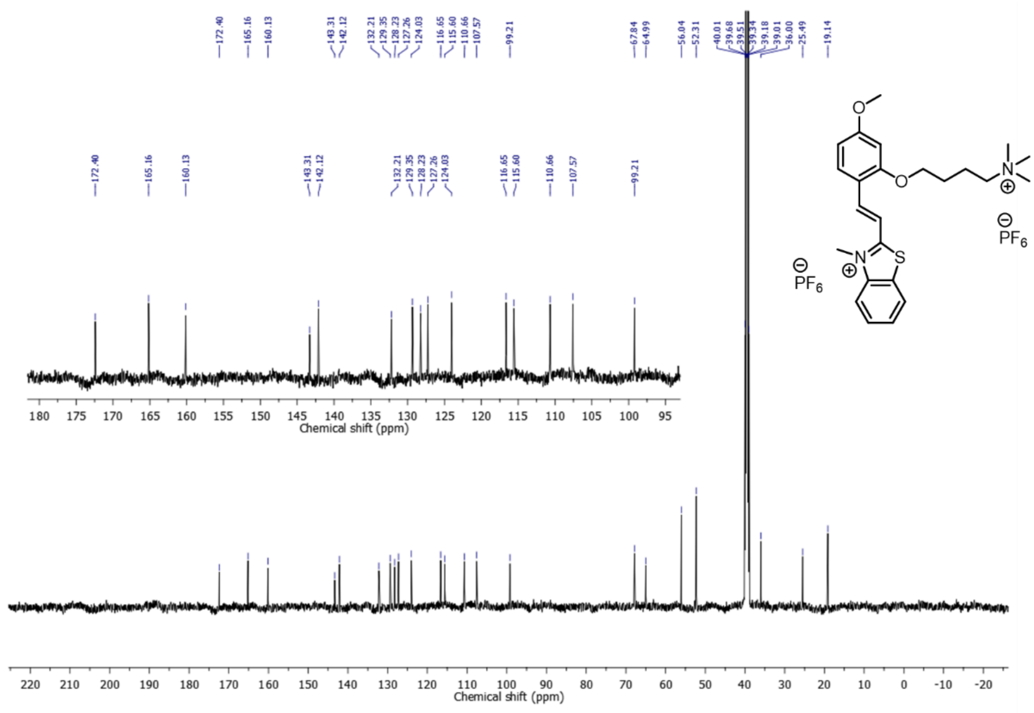

(b)

Figure S40. ^{1}H (500 MHz, DMSO-d_6) (a) and ^{13}C (126 MHz, DMSO-d_6) (b) NMR spectra of compound **BT2+(OMe)**

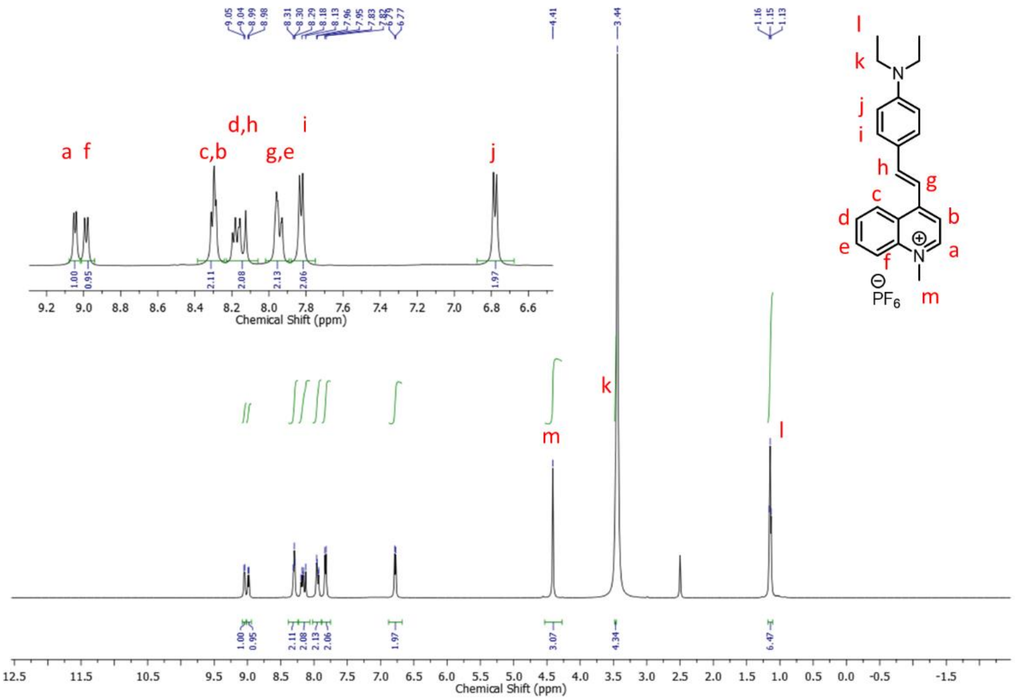

(a)

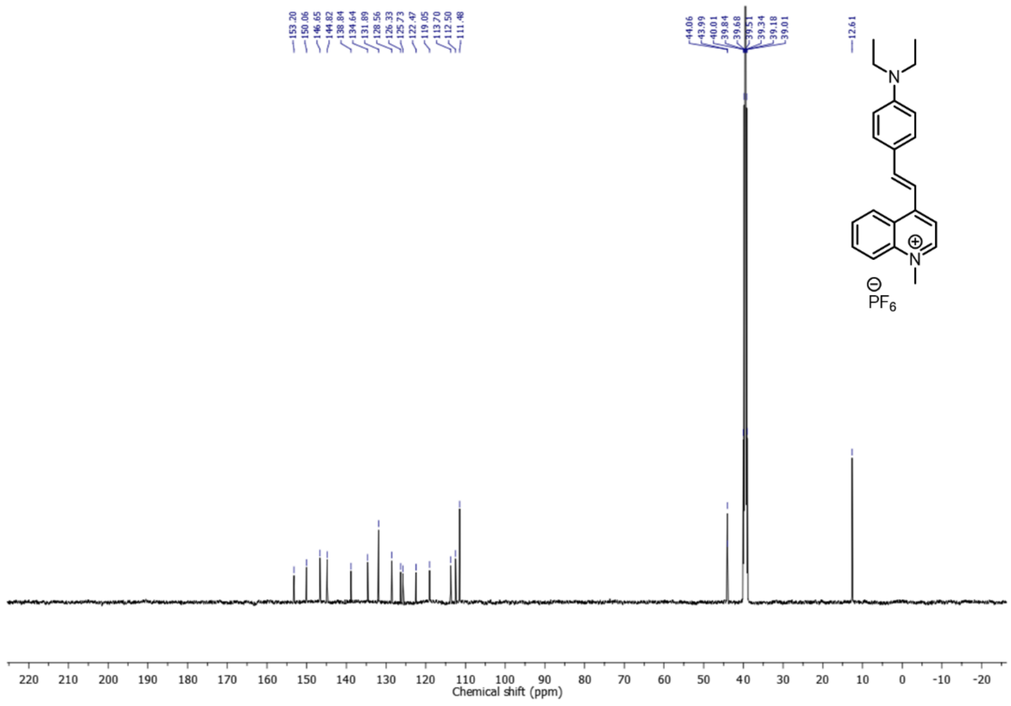

(b)

Figure S41. ^{1}H (500 MHz, DMSO-d_6) (a) and ^{13}C (126 MHz, DMSO-d_6) (b) NMR spectra of compound **4QL+**

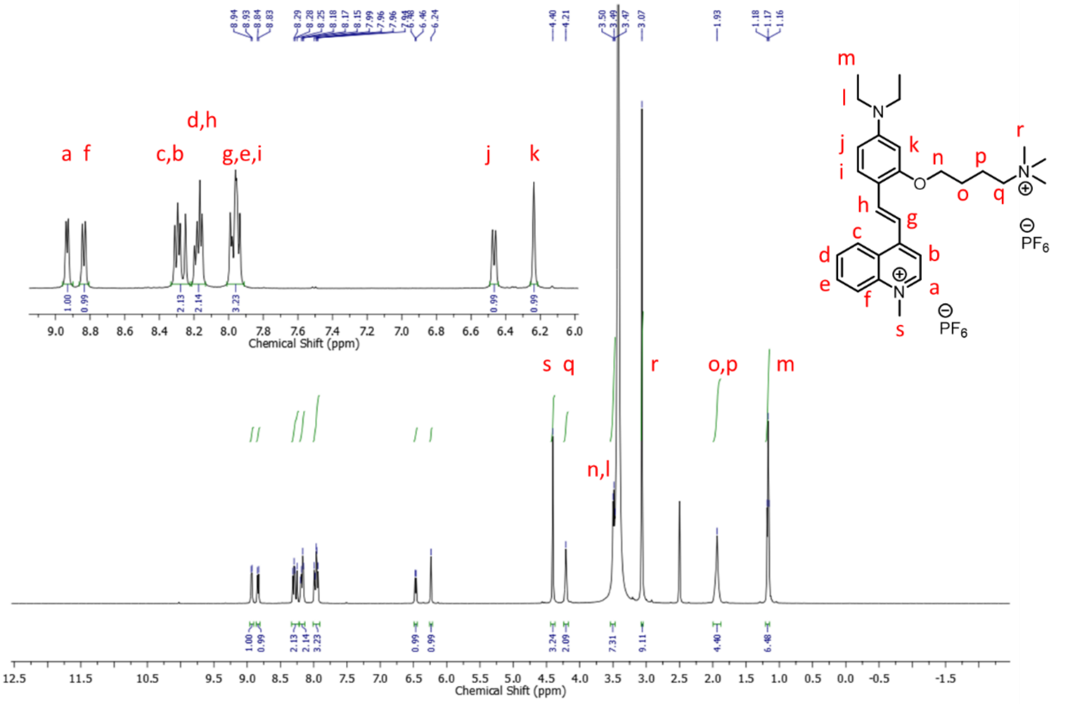

(a)

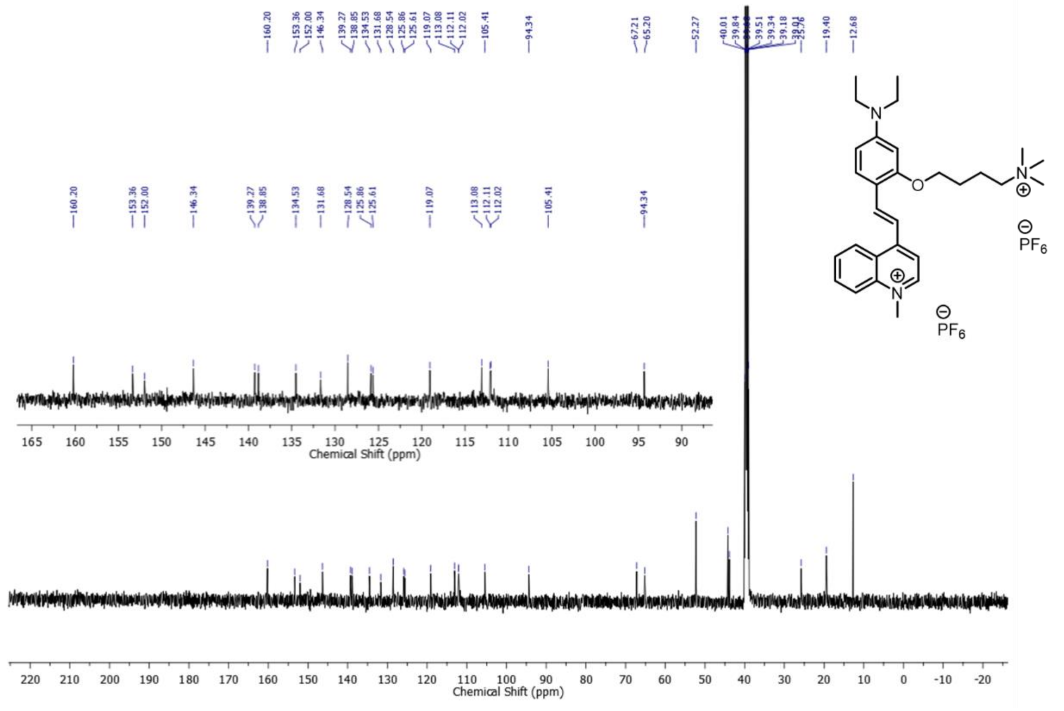

(b)

Figure S42. ^{1}H (500 MHz, DMSO-d_6) (a) and ^{13}C (126 MHz, DMSO-d_6) (b) NMR spectra of compound **4QL2+**

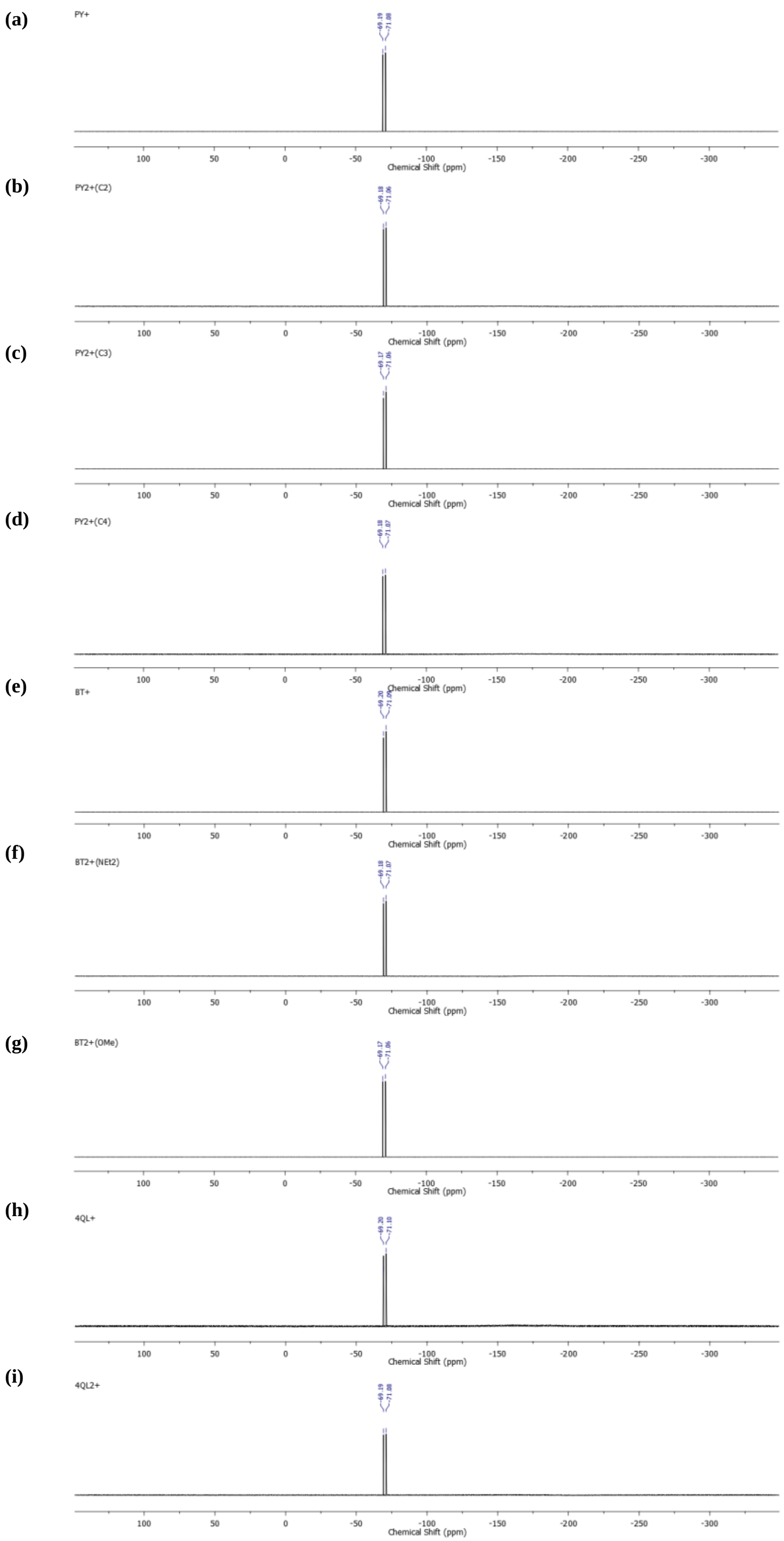

Figure S43. ^{19}F (376 MHz, DMSO-d_6) NMR spectra of compounds **PY+** (a), **PY2+(C2)** (b), **PY2+(C3)** (c), **PY2+(C4)** (d), **BT+** (e), **BT2+(NEt$_2$)** (f), **BT2+(OMe)** (g), **4QL+** (h), and **4QL2+** (i)

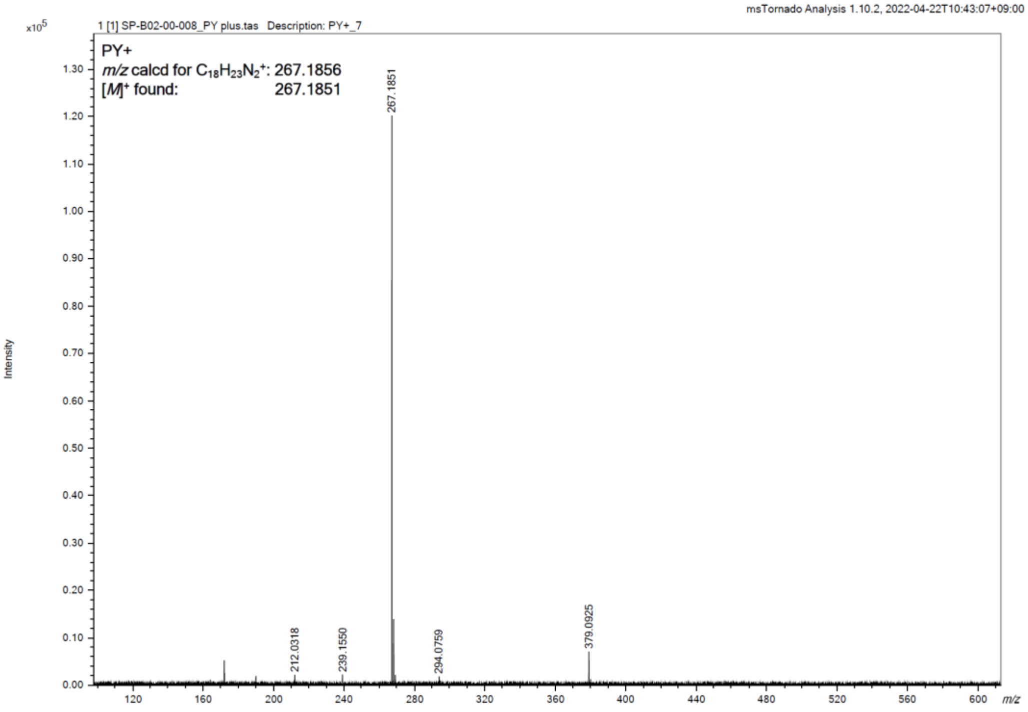

(a)

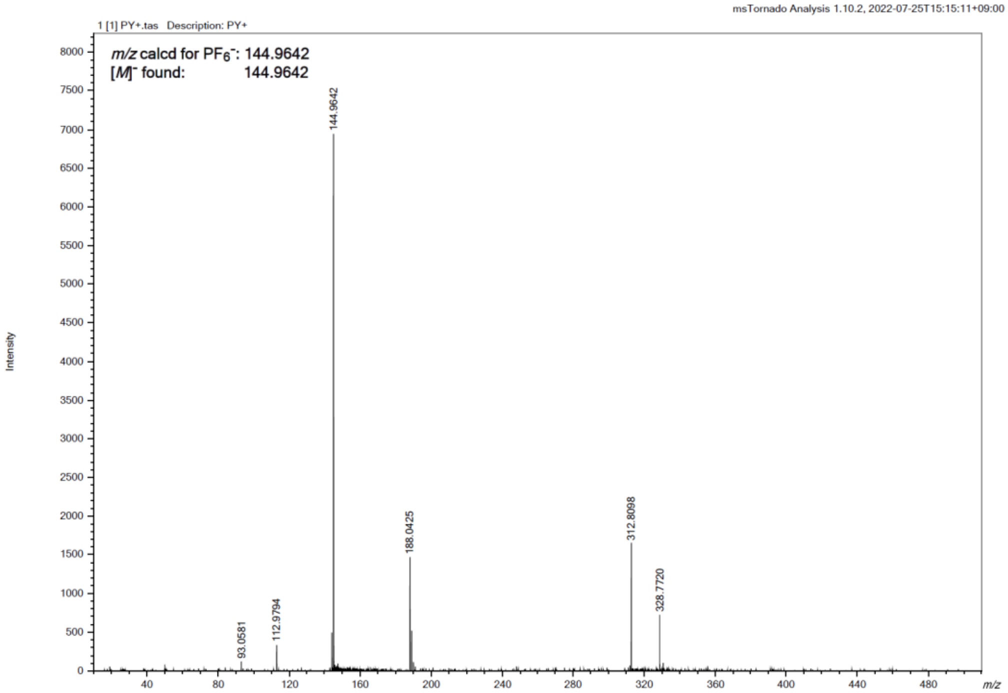

(b)

Figure S44. HRMS (MALDI-TOF) of **PY+** in positive (a) and negative (b) modes

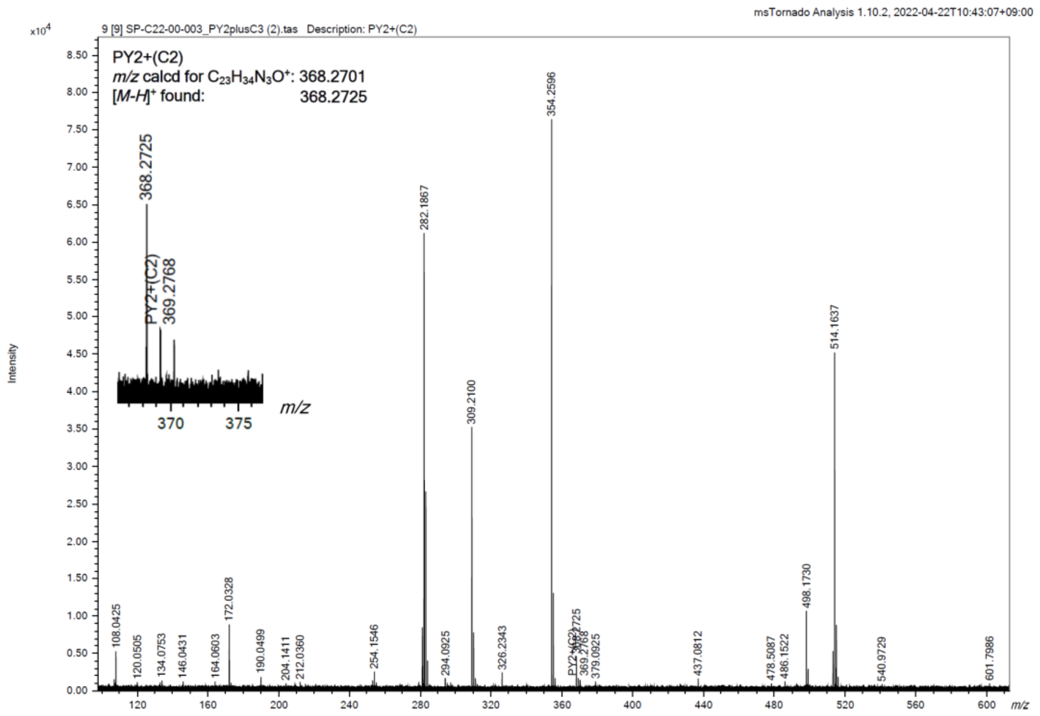

(a)

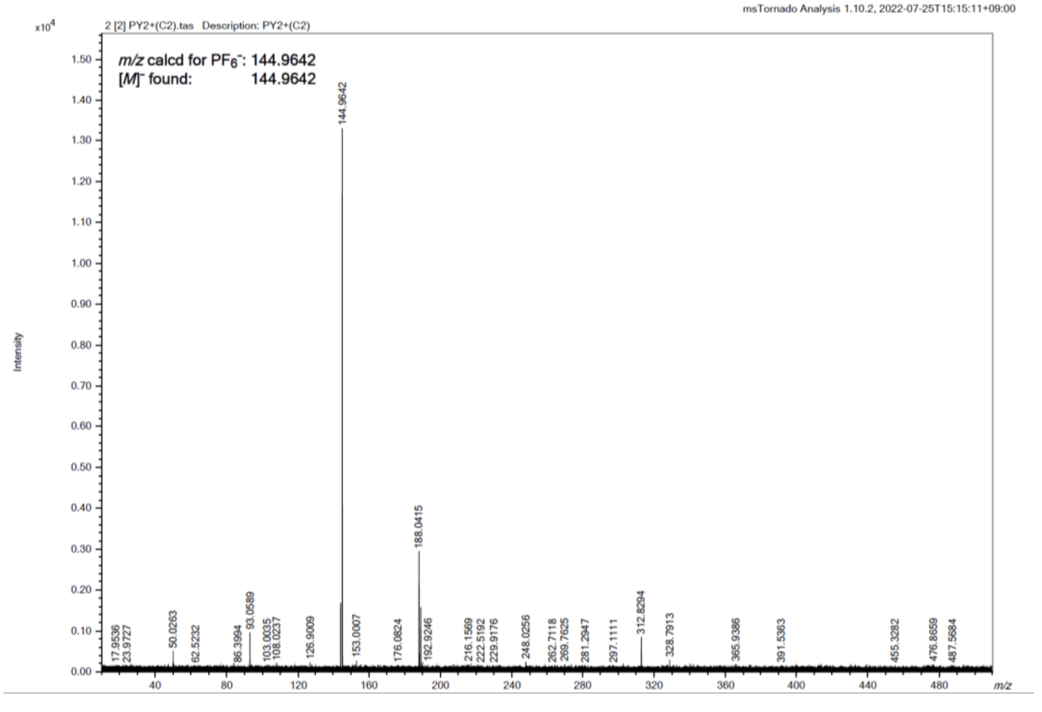

(b)

Figure S45. HRMS (MALDI-TOF) of **PY2+(C2)** in positive (a) and negative (b) modes

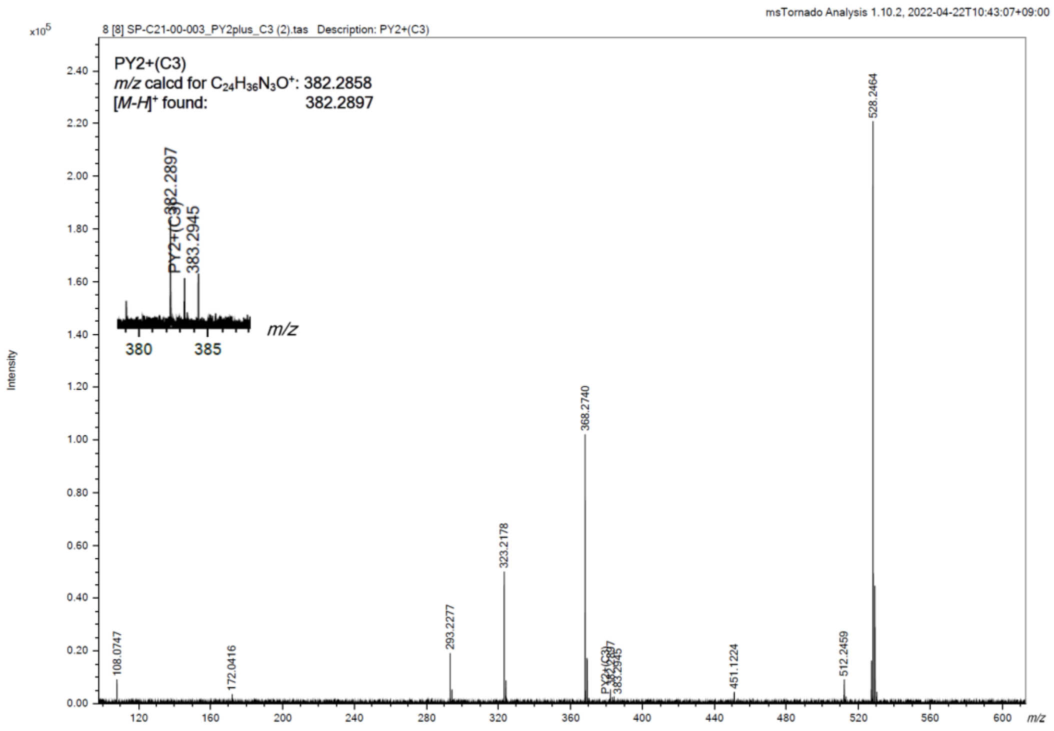

(a)

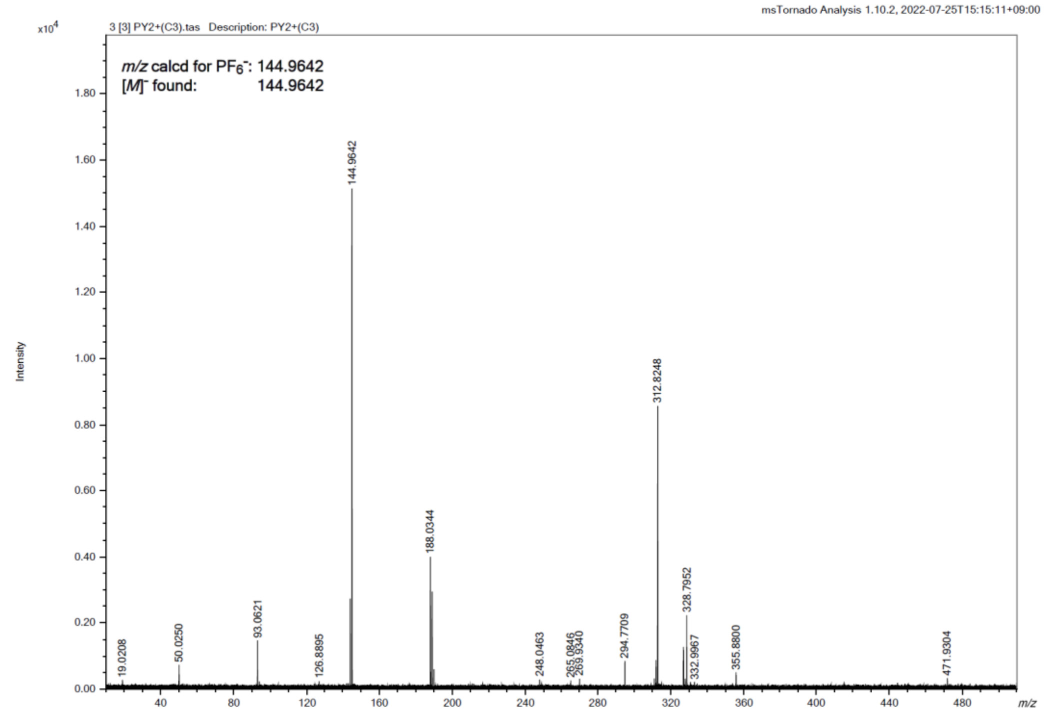

(b)

Figure S46. HRMS (MALDI-TOF) of **PY2+(C3)** in positive (a) and negative (b) modes

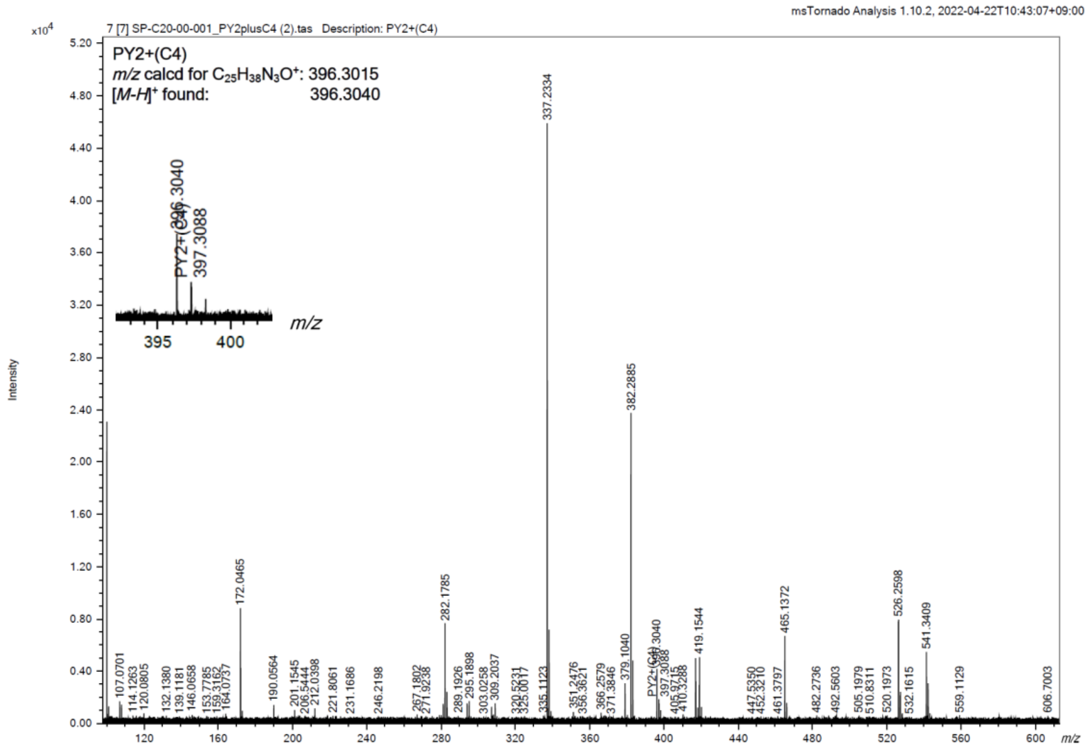

(a)

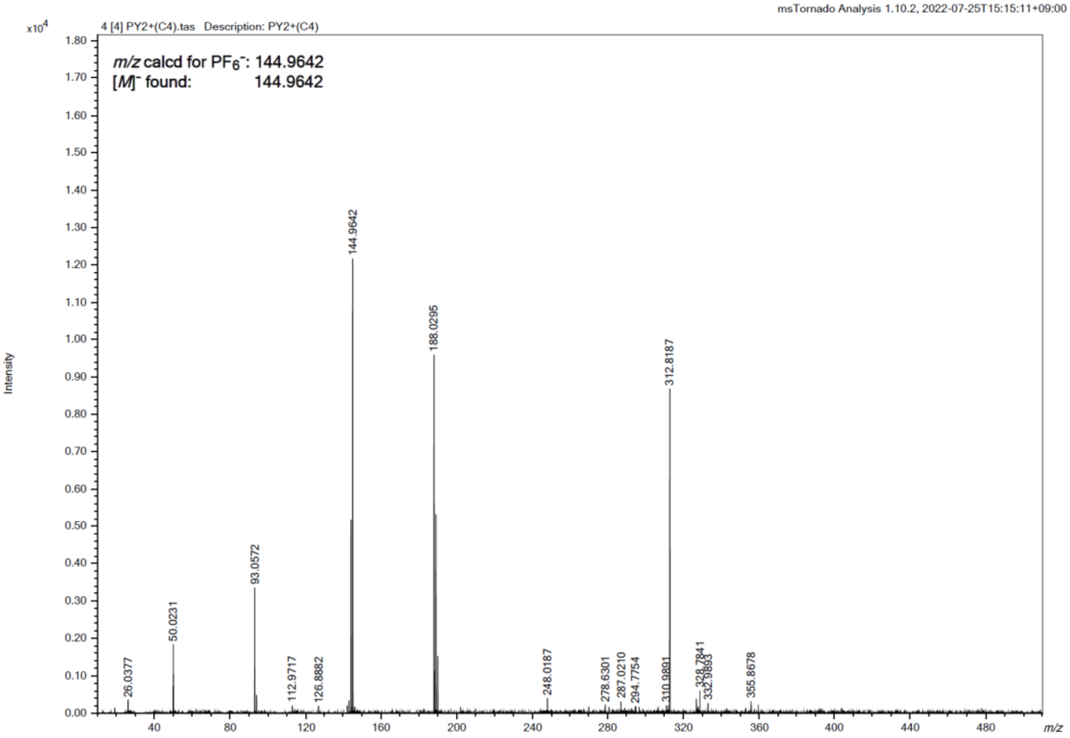

(b)

Figure S47. HRMS (MALDI-TOF) of **PY2+(C4)** in positive (a) and negative (b) modes

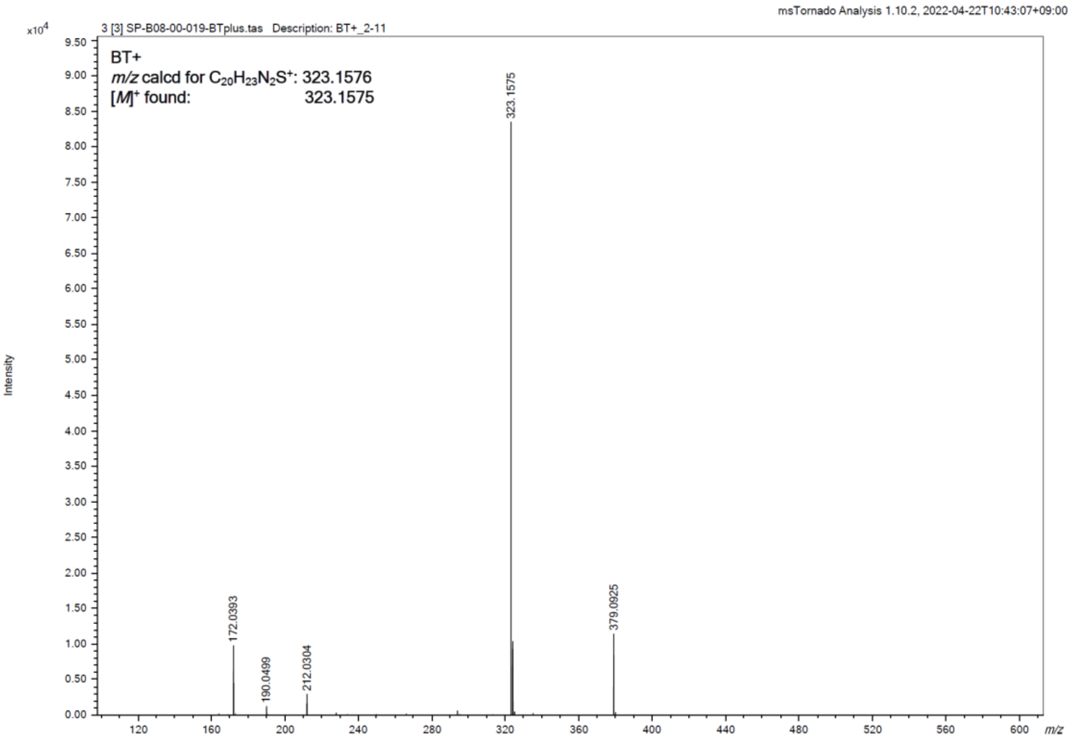

(a)

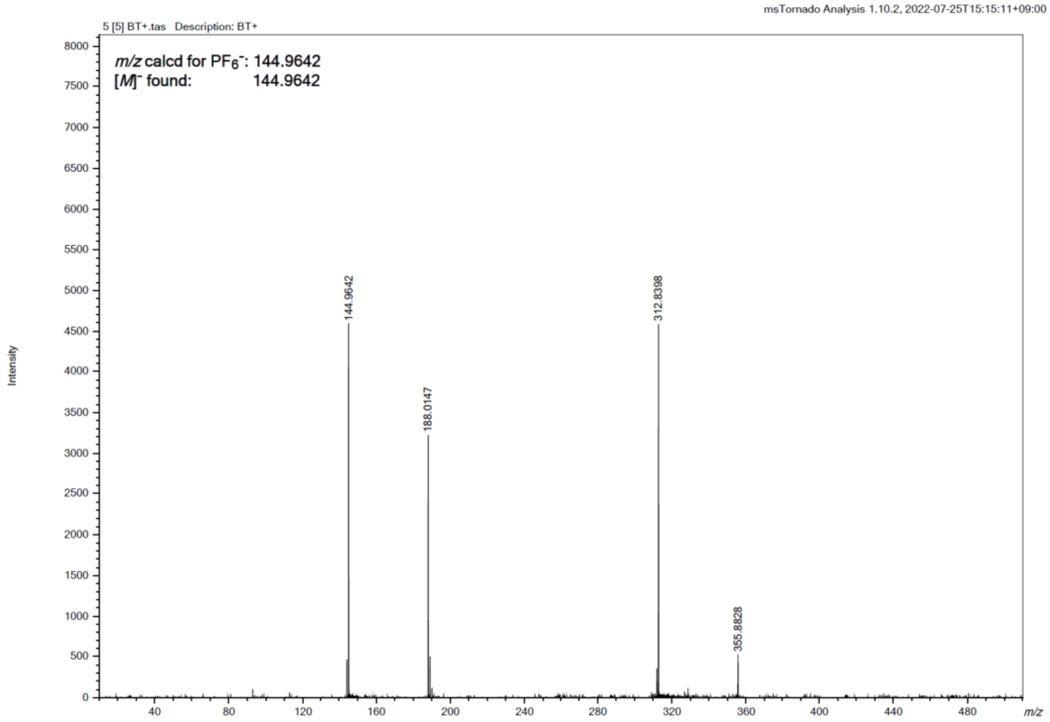

(b)

Figure S48. HRMS (MALDI-TOF) of **BT+** in positive (a) and negative (b) modes

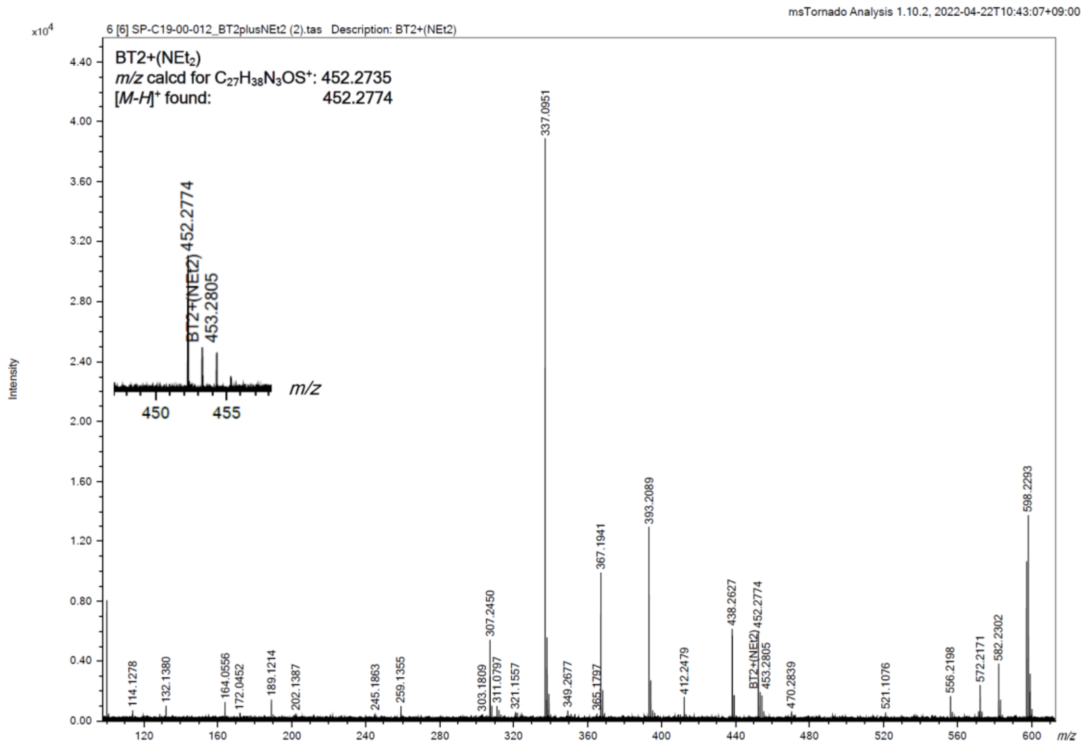

(a)

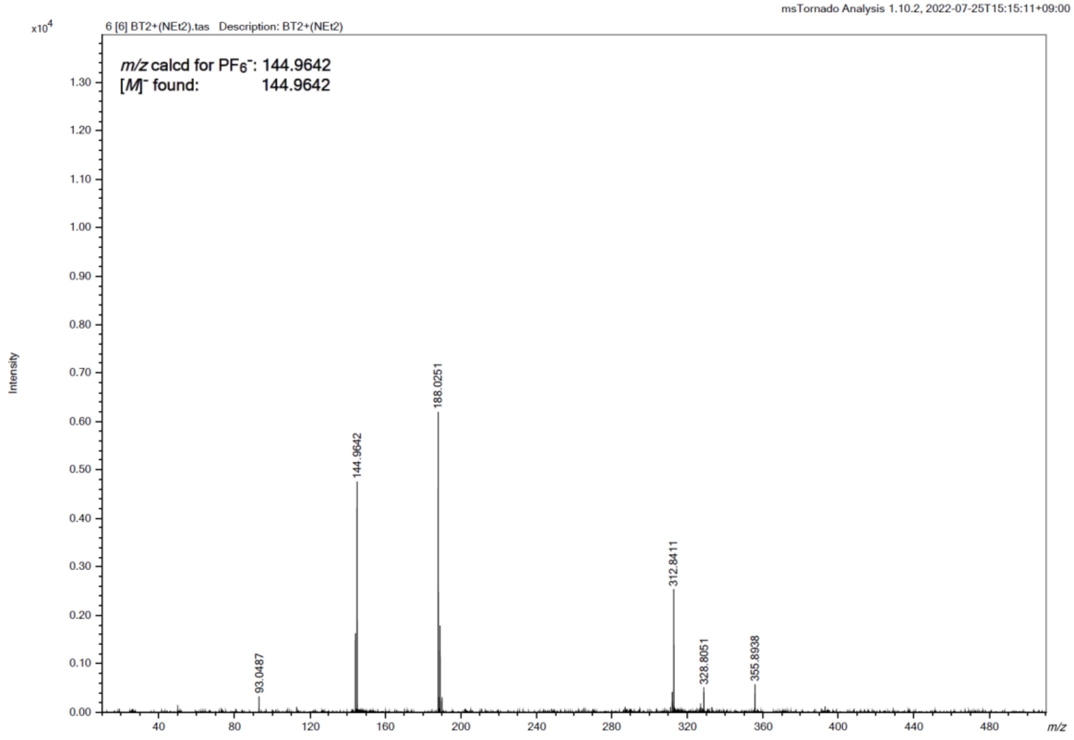

(b)

Figure S49. HRMS (MALDI-TOF) of **BT2+(NEt$_2$)** in positive (a) and negative (b) modes

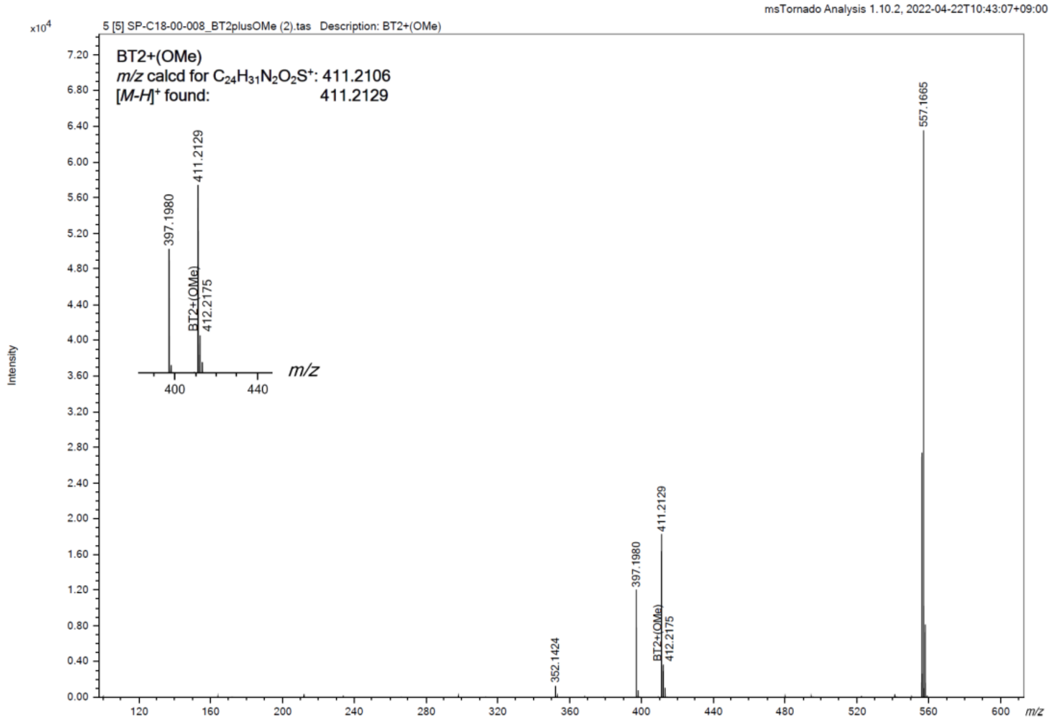

(a)

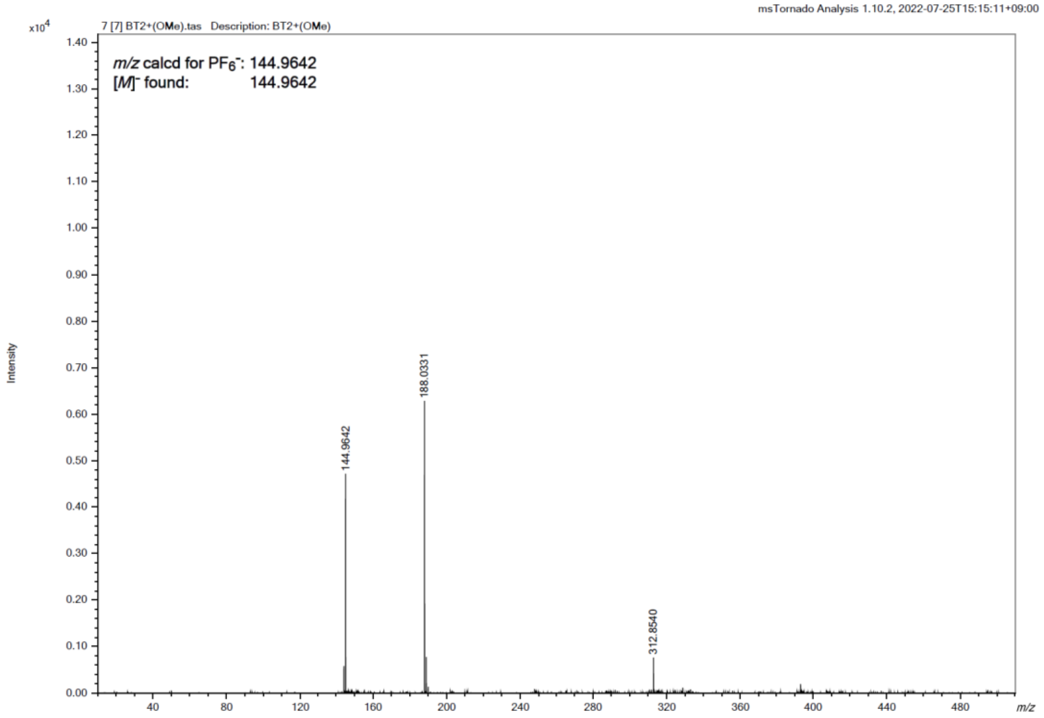

(b)

Figure S50. HRMS (MALDI-TOF) of **BT2+(OMe)** in positive (a) and negative (b) modes

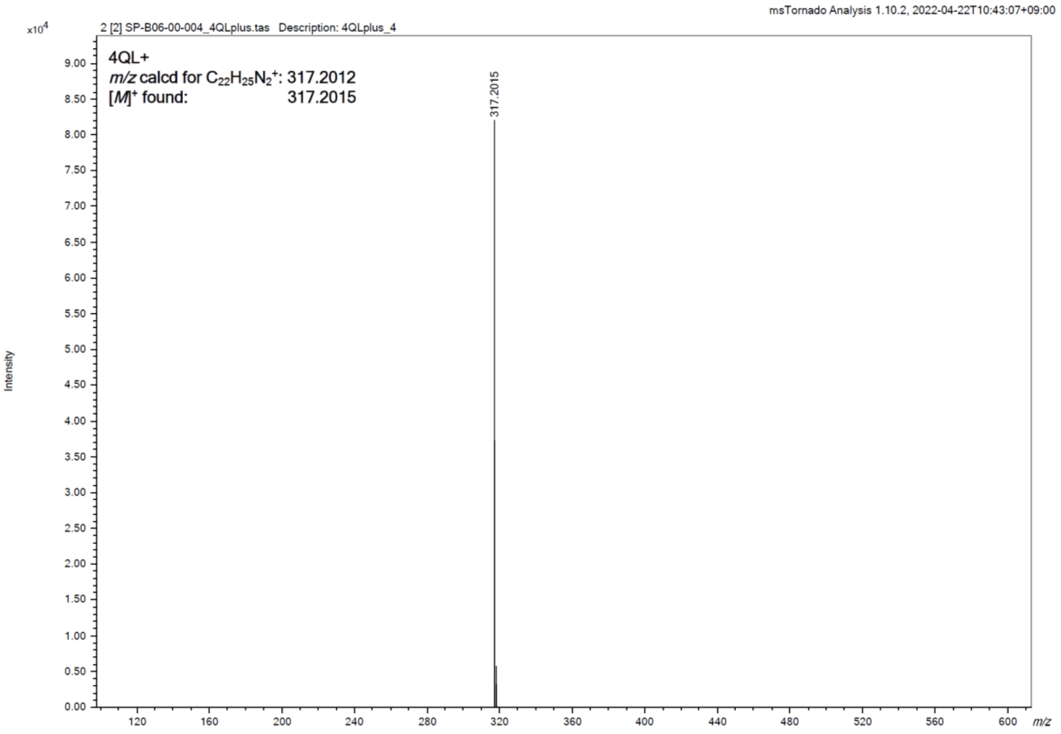

(a)

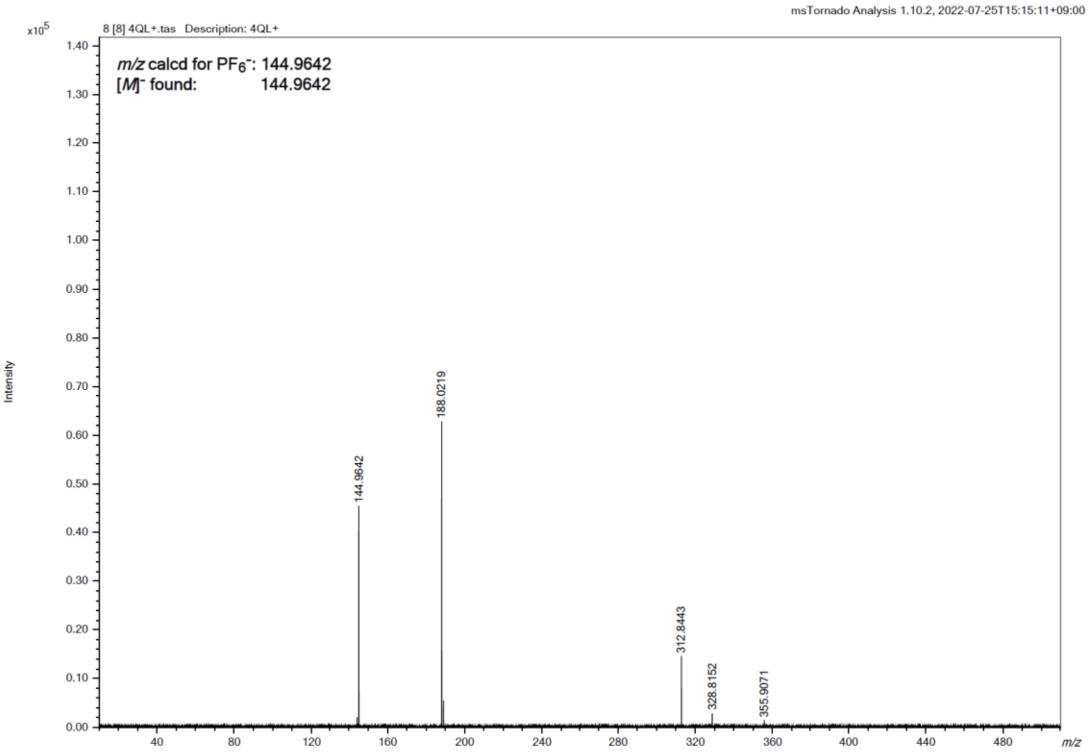

(b)

Figure S51. HRMS (MALDI-TOF) of **4QL+** in positive (a) and negative (b) modes

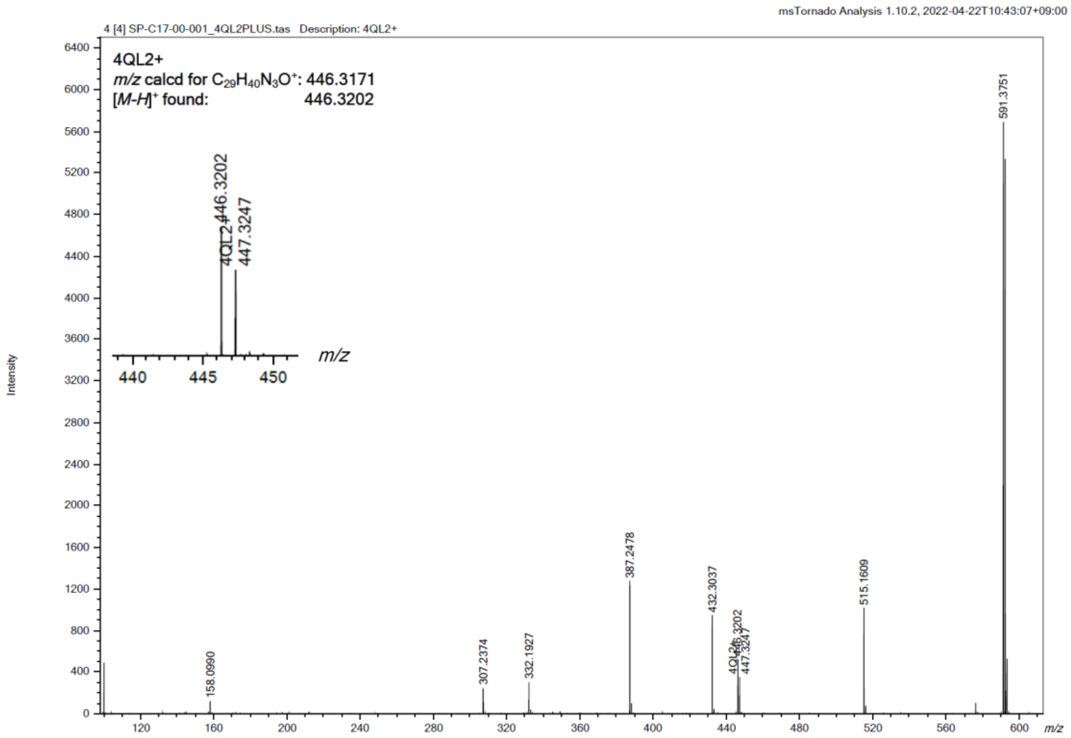

(a)

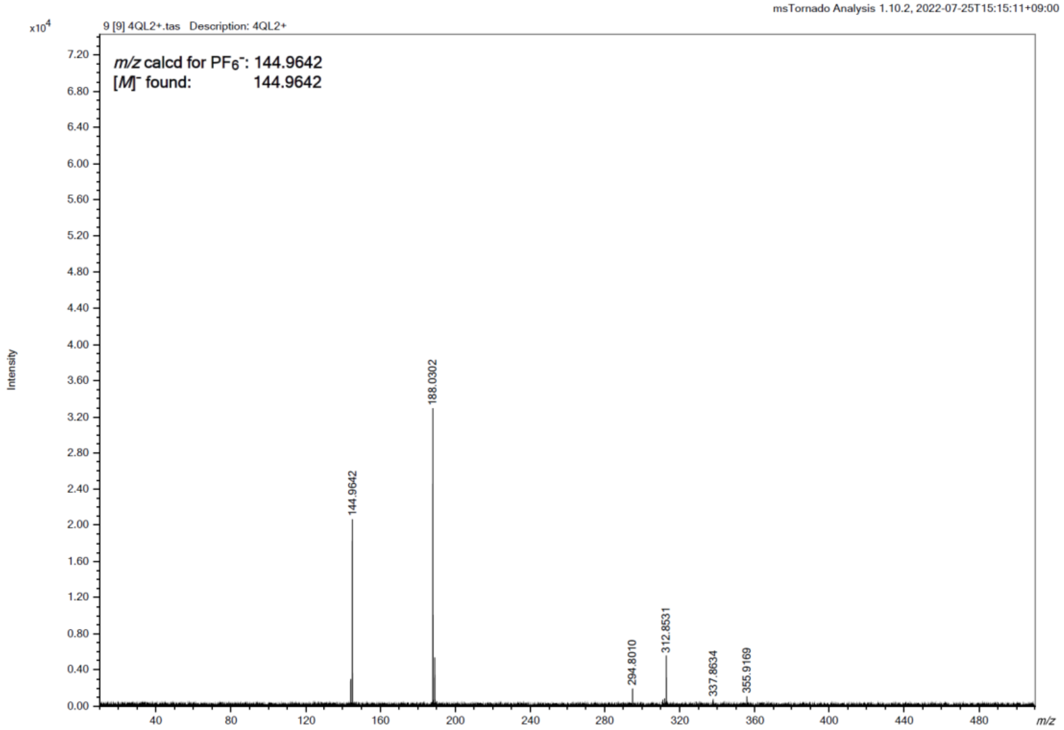

(b)

Figure S52. HRMS (MALDI-TOF) of **4QL2+** in positive (a) and negative (b) modes

Table S1. Optical properties of **4QL2+** in various solvents: absorption maxima λ_{max}(abs), emission maxima λ_{max}(em), and stroke shifts

Solvent	Dielectric Constant	Refractive Index	Δf	λ_{max}(abs)	λ_{max}(em)	Stokes Shift (cm^{-1})	Relative Fluorescence
**Water**	80.2	1.333	0.3201	546	660	3164	1
**DMSO**	47.24	1.478	0.2637	574	682	2759	15.8
**EtOH**	25.3	1.361	0.2898	580	665	2204	12.7
**MeOH**	33	1.328	0.3089	576	664	2301	6.90
**DCM**	9.14	1.425	0.2186	592	661	1763	54.0
**MeCN**	36.64	1.344	0.3050	569	675	2760	3.84
**EtOAc**	6.081	1.372	0.2008	573	660	2300	8.83
**Glycerol**	46.5	1.474	0.2647	579	665	2234	55.3
**Acetone**	21	1.359	0.2847	572	669	2535	6.25

Table S2. DNA sequences used in this study

Name	Sequence	ε_{260}[a]
dsDNA	5'-CCAGGGCATGGTAGATCACTGTACGCCGCG-3'	285.2
	3'-GGTCCCGTACCATCTAGTGACATGCGGCGC-5'	279.0
$d(AT)_{10}$	5'-ATATATATATATATATATAT-3'	221.7
$d(GC)_{10}$	5'-GCGCGCGCGCGCGCGCGCGC-3'	167.9

[a] http://www.chemistry.sc.chula.ac.th/dna/, calculated according to nearest-neighbor method, see http://www.owczarzy.net/extinctionDNA.htm

Table S3. Primer sequences for LAMP-**4QL2+** assay

Bacteria (gene)	Primer names	Primer sequences (5'-3')	Reference
E. coli (*phoA*)	Ec phoA F3	AAGTTGAAGGTGCGTCAAT	Stratakos *et al*., 2017[1]
	Ec phoA B3	CTTGTGAATCCTCTTCGGAG	
	Ec phoA FIP	GTGATCAGCGGTGACTATGACCTCTCGATGAAGCCGTAC A	
	Ec phoA BIP	ATTGTCGCGCCGGATACCCTCATCACCATCACTGCG	
	Ec phoA LF	AGCGTGTTGCCATCCTTT	
	Ec phoA LB	CAGGCGCTAAATACCAAAGATG	
S. aureus (sea)	SEA F3	TCAATTTATGGCTAGACGGT	Somboonna *et al.*, 2019[2]
	SEA B3	CTTGAGCACCAAATAAATCG	
	SEA FIP	GATCCAACTCCTGAACAGTTACAATACAGTACCTTTGGAA ACG	
	SEA BIP	CTGATGTTTTTGATGGGAAGGTTCCCGAAGG TTCTGTAGAAGT	
	SEA LF	CGTCAGTTAAAAATGTCGTATGAT	
	SEA LB	AGAGGGGATTAATCGTGTT TCA	

Table S4. Positive and negative control bacterial strains for specificity test

Bacteria	Positive controls	Negative controls
E. coli	*Escherichia coli* ATCC 25922<br>*Escherichia coli* MCCU 0349	*Enterobacter cloacae* N/A<br>*Staphylococcus aureus* ATCC 25923
S. aureus	*Staphylococcus aureus* ATCC 25923<br>*Staphylococcus aureus* MCCU0357<br>*Staphylococcus aureus* MCCU0370	*Staphylococcus epidermidis* ATCC 12228<br>*Staphylococcus saprophyticus* ATCC 15305

Reference

1 Stratakos, A. C., Linton, M., Millington, S. & Grant, I. R. A loop-mediated isothermal amplification method for rapid direct detection and differentiation of nonpathogenic and verocytotoxigenic Escherichia coli in beef and bovine faeces. *J Appl Microbiol* **122**, 817-828, doi: https://doi.org/10.1111/jam.13381 (2017).

2 Somboonna, N., Vilaivan, T., Junpra-ob, S., Supabowornsathit, K. & Ditmangklo, B. A method for the detection of pathogens in foods and drinks by loop-mediated isothermal amplification technique in combination with double stranded DNA staining dyes. *Thai Petty Patent Application No. 11926300006 (20 Sep 2019)*.
